# Designing multi-target-directed flavonoids: a strategic approach to Alzheimer's disease†

**DOI:** 10.1039/d3sc00752a

**Published:** 2023-08-03

**Authors:** Seongmin Park, Mingeun Kim, Yuxi Lin, Mannkyu Hong, Geewoo Nam, Adam Mieczkowski, József Kardos, Young-Ho Lee, Mi Hee Lim

**Affiliations:** a Department of Chemistry, Korea Advanced Institute of Science and Technology (KAIST) Daejeon 34141 Republic of Korea miheelim@kaist.ac.kr; b Research Center for Bioconvergence Analysis, Korea Basic Science Institute (KBSI) Ochang Chungbuk 28119 Republic of Korea mr0505@kbsi.re.kr; c Center for Catalytic Hydrocarbon Functionalizations, Institute for Basic Science (IBS) Daejeon 34141 Republic of Korea; d Institute of Biochemistry and Biophysics, Polish Academy of Sciences Pawińskiego 5a 02-106 Warsaw Poland; e ELTE NAP Neuroimmunology Research Group, Department of Biochemistry, Institute of Biology, ELTE Eötvös Loránd University Budapest 1117 Hungary; f Bio-Analytical Science, University of Science and Technology (UST) Daejeon 34113 Republic of Korea; g Graduate School of Analytical Science and Technology, Chungnam National University Daejeon 34134 Republic of Korea; h Department of Systems Biotechnology, Chung-Ang University (CAU) Gyeonggi 17546 Republic of Korea; i Frontier Research Institute for Interdisciplinary Sciences (FRIS), Tohoku University Sendai Miyagi 980-8578 Japan

## Abstract

The underlying causes of Alzheimer's disease (AD) remain a mystery, with multiple pathological components, including oxidative stress, acetylcholinesterase, amyloid-β, and metal ions, all playing a role. Here we report a strategic approach to designing flavonoids that can effectively tackle multiple pathological elements involved in AD. Our systematic investigations revealed key structural features for flavonoids to simultaneously target and regulate pathogenic targets. Our findings led to the development of a highly promising flavonoid that exhibits a range of functions, based on a complete structure–activity relationship analysis. Furthermore, our mechanistic studies confirmed that this flavonoid's versatile reactivities are driven by its redox potential and direct interactions with pathogenic factors. This work highlights the potential of multi-target-directed flavonoids as a novel solution in the fight against AD.

## Abstract 2

Rational structure–mechanism-based strategies for identifying small molecules that are able to control multiple pathological targets in Alzheimer's disease are established, with an example of developing a promising multi-target-directed flavonoid.

## Graphical abstract

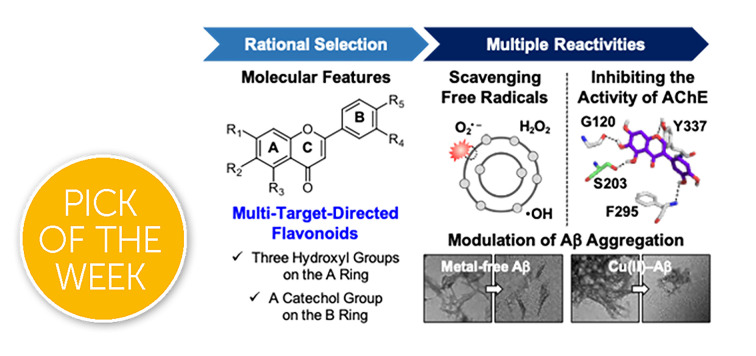

## Introduction

**Fig. 1:**
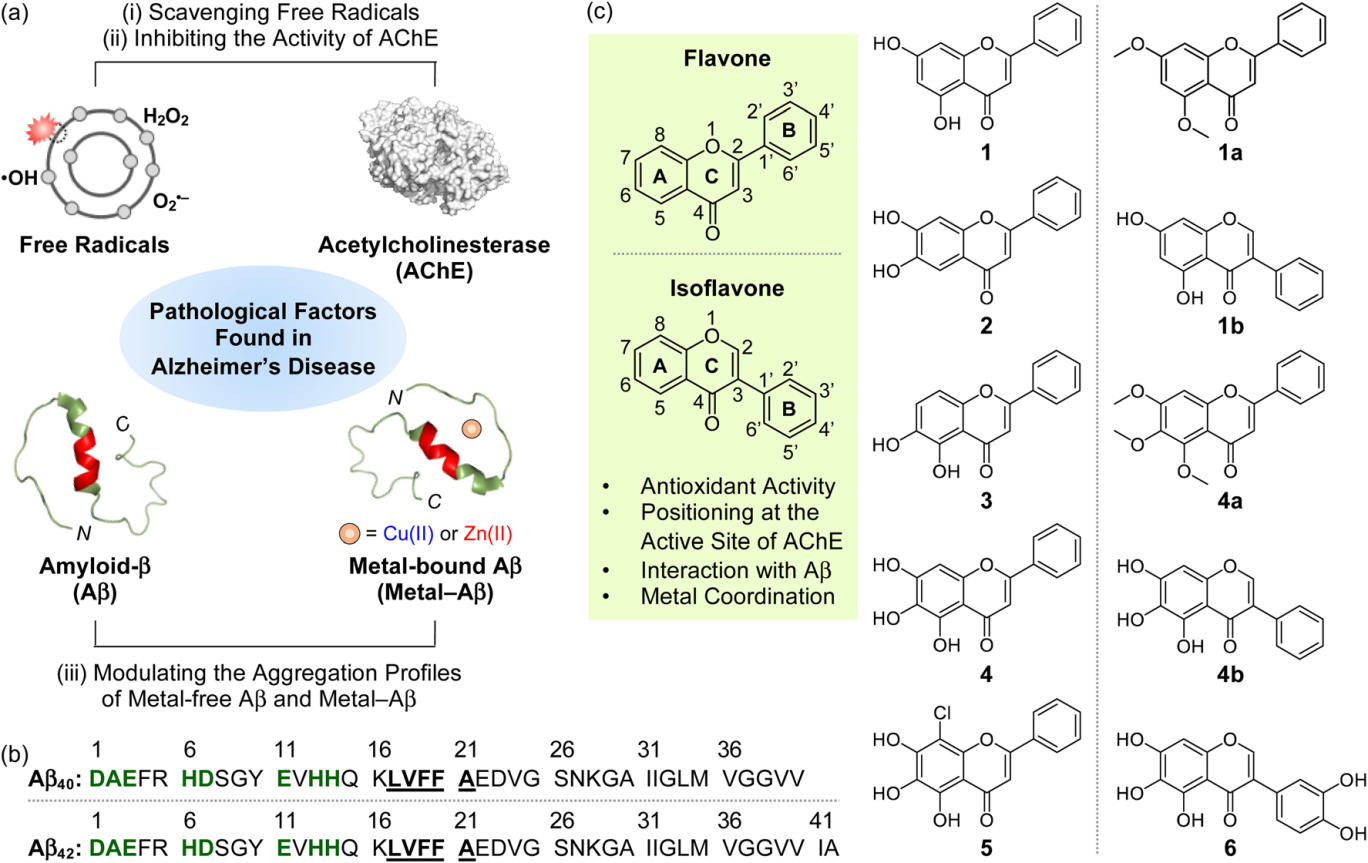
Multiple pathogenic factors and flavonoids studied in this work. (a) Free radicals, AChE (PDB 1C2B^61^), metal-free Aβ (PDB 2LFM^16^), and metal–Aβ as pathological elements to be controlled in this work. (b) Sequences of Aβ peptides. Amino acid residues involved in the metal-binding and self-recognition sites are highlighted in bold/green and bold/underline, respectively. (c) Chemical structures of flavonoids 1–5, 1a, 1b, 4a, 4b, and 6. 1, chrysin; 2, 6,7-dihydroxyflavone; 3, 5,6-dihydroxyflavone; 4, baicalein; 5, 8-chlorobaicalein; 1a, 5,7-dimethoxyflavone; 1b, 5,7-dihydroxyisoflavone; 4a, 5,6,7-trimethoxyflavone; 4b, 5,6,7-trihydroxyisoflavone; 6, 3-(3,4-dihydroxyphenyl)-5,6,7-trihydroxy-4*H*-chromen-4-one.

The intertwined network among multiple pathological factors, such as free radicals, acetylcholinesterase (AChE), metal-free amyloid-β (Aβ), and metal-bound Aβ (metal–Aβ) (Fig. 1a and b), has given the difficulty in combating Alzheimer's disease (AD).^1–5^ Oxidative stress characterized by dysregulated free radicals can damage lipids, nucleic acids, and proteins, which leads to organelle dysfunction and cell death.^6–8^ As illustrated in the cholinergic hypothesis, AChE is associated with the development of AD.^3,9^ The synaptic concentration of the neurotransmitter acetylcholine (ACh) is adjusted by the catalytic activity of AChE;^10–12^ however, the abnormally reduced level of ACh can be driven by the increased amount of AChE.^3,10,13^ In addition, highly concentrated AChE within senile plaques (SPs) can co-localize and interact with Aβ species, resulting in the AChE–Aβ complexation and the acceleration of amyloid aggregation.^13^ Aβ peptides tend to aggregate into toxic self-assembled aggregates, such as oligomers and fibrils.^14–19^ In particular, metastable and structured Aβ oligomers are known to trigger membrane disruption, abnormal cellular signaling, and organelle dysfunction.^1,9,15^ High concentrations of metal ions [*e.g.*, Cu(i/ii) and Zn(ii)] are also found in the SPs of AD patients, and they can affect the conformation and aggregation behaviors of Aβ by their coordination to Aβ.^20–24^ Moreover, redox-active Cu(i/ii)-bound Aβ species can produce reactive oxygen species (ROS) *via* Fenton-like reactions and, consequently, aggravate oxidative stress.^25–29^

Several pharmacological trials employing antioxidants (*e.g.*, vitamin E),^30,31^ AChE inhibitors (*e.g.*, donepezil, galantamine, rivastigmine),^3,25^ and anti-Aβ drugs (*e.g.*, aducanumab and lecanemab)^32–34^ have been attempted to prevent the progression of AD. Such single target-based therapeutics can temporarily relieve symptoms,^3,25^ and their efficacies are still questionable, however.^9,35^ Thus, significant endeavors in designing chemical reagents capable of targeting and modulating two or more pathogenic elements have been made to elucidate the complex pathology of AD and discover effective therapeutic candidates.^3,9,24,29,36^ Herein, we illustrate a strategic approach to designing flavonoids that can target multiple pathogenic factors, including free radicals, AChE, metal-free Aβ, and metal–Aβ, as depicted in Fig. 1a and b, and modulate their reactivities (*vide infra*). We identified key structural features shown in flavonoids that are responsible for versatile reactivities against desired pathological targets through systematic investigations. Moreover, a highly promising flavonoid with multiple functions was developed based on our structure–activity relationship analysis, with the determination of molecular-level mechanistic details. Our overall studies demonstrate the potential of flavonoids as multifunctional chemical reagents for AD and serve as a roadmap for the future development of multi-target-directed small molecules in the fight against neurodegenerative disorders, such as AD.

## Results and discussion

### Selection and preparation of flavonoids

Given that flavonoids as a family of naturally occurring polyphenols have antioxidant,^37,38^ antiviral,^39^ and antimicrobial activities,^40^ we chose the flavonoid framework for our molecular design of small molecules with reactivities of multiple pathological targets. As presented in Fig. 1c, some structural moieties of flavonoids are reported to be important for modulating free radicals, AChE, metal-free Aβ, and metal–Aβ: (i) The unsaturated bond between C2 and C3 on the C ring of flavonoids could interact with the hydrophobic self-recognition (Leu17–Ala21) and C-terminal regions of Aβ that are essential for its aggregation process;^41–44^ (ii) the 4-oxo functionality on the C ring as an oxygen (O) donor atom can participate in metal coordination;^45^ (iii) the chromone moiety (A and C rings) is responsible for the reactivity of flavonoids against AChE through the interactions with amino acid residues containing an aromatic ring at their side chains (*e.g.*, Trp86, Phe295, and Tyr341) within the hydrophobic binding pocket of AChE;^46^ (iv) both the number and position of hydroxyl groups are associated with chemical properties, such as redox potentials, hydrogen bonding, and aqueous solubility.^47^

In addition to the abovementioned structural features of flavonoids, the incorporation of hydroxyl groups on the B and C rings and the isoflavone variation to regulate pathological factors have been recently identified;^45,46,48^ however, the information on structural and chemical properties on the A ring for multiple reactivities towards our desired targets, except for AChE,^49^ is very limited. Thus, to gain a full spectrum of the structure–activity relationship with respect to all the A, B, and C rings, we rationally selected a series of nine flavonoids (1–5, 1a, 1b, 4a, and 4b; Fig. 1c) through the structural variation of functional groups on the A ring and translocation of the B ring from C2 to C3 that can alter their electronic and AChE-/Aβ-interacting properties. Flavonoids 1–3 containing two hydroxyl groups located at distinct positions on the A ring were first chosen. Our series also included 4 possessing three hydroxyl groups on the A ring. Flavonoid 5 that embodies three hydroxyl groups with an electron-withdrawing chloro group at the C8 position on the A ring was additionally selected. Moreover, 1a/4a (substituted with methoxy groups instead of hydroxyl groups) and 1b/4b (isoflavone derivative), which originate from 1 and 4, respectively, were also included in our series. Flavonoids were obtained from commercially available sources (1–4, 1a, 1b, and 4a) or prepared following previously reported synthetic routes (5 ^50^ and 4b ^51^). The characterization of 5 and 4b was summarized in Fig. S1 and S2,† respectively. The investigations of how the structural variations of the flavonoids shown in Fig. 1c on the A ring alter their reactivities against multiple pathological factors, with previously reported structure–reactivity studies using a series of flavonoids chosen based on distinct structural modifications on the B and C rings,^45,46,48^ led to the development of a promising multi-target-directed flavonoid (6; Fig. 1c) (*vide infra*).

### Redox potentials and scavenging free radicals

**Fig. 2:**
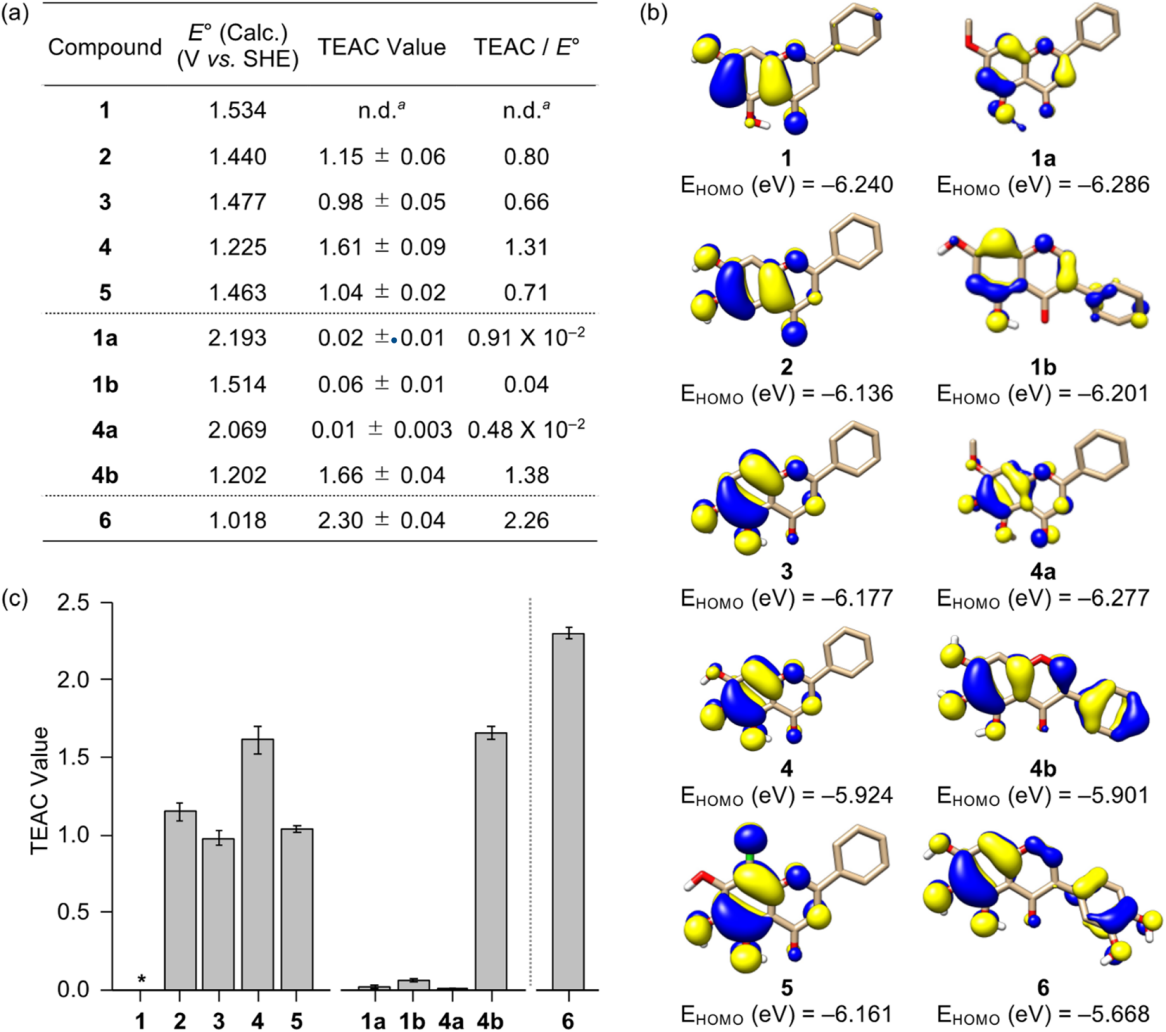
Redox properties of flavonoids and their ability to quench free organic radicals. The (a) redox potentials (*E*° *vs.* SHE) of the flavonoids were calculated with the (b) isosurface plots (isodensity value = 0.03 a.u.) of their HOMOs. (a and c) TEAC values of the flavonoids determined by the TEAC assay. Conditions: EtOH; 25 °C; *λ*_abs_ = 734 nm. ^*a*^n.d., not determined. *Note that the TEAC value of 1 could not be obtained due to its limited solubility in EtOH.

The redox activity of small molecules is critical for controlling multiple pathological features of AD, including free radicals, metal-free Aβ, and metal–Aβ.^47,52–55^ In particular, redox properties of small molecules are connected with their antioxidant ability.^56^ Thus, the redox potential of flavonoids were first computed following the previously reported density functional theory (DFT) calculation.^57^ It should be noted that we could not experimentally obtain the flavonoids' redox potentials due to their instability and limited solubility in aqueous or organic media used for their electrochemical measurements. As illustrated in Fig. 2a, the calculated redox potentials were varied with the number and position of electron-donating hydroxyl groups on the A ring. Based on the position of hydroxyl groups on the A ring shown in 1–3, their *E*° values *vs.* standard hydrogen electrode (SHE) were ranged from 1.440–1.534 V. In detail, the incorporation of two hydroxyl substituents *ortho*-positioned at either C6/C7 (2) or C5/C6 (3) decreased the redox potentials, relative to that at the *meta*-position (C5/C7, 1). Furthermore, 4 with an additional hydroxyl group on the A ring revealed a lower redox potential; however, incorporating an electron-withdrawing chloro substituent at the C8 position of 4, designated as 5, yielded an similar level of *E*° value comparable to that of 2 and 3. The substitution of hydroxyl groups with methoxy moieties on the A ring, as presented in 1a/4a, and the alteration of the position of the B ring, as shown in 1b/4b, resulted in relatively higher and lower calculated *E*° values, respectively, when compared to their respective parent structures (1 and 4).

To obtain a greater understanding of computed redox potentials, the highest occupied molecular orbitals (HOMOs) of 1–5, 1a, 1b, 4a, and 4b were analyzed. As displayed in Fig. 2b, 1 exhibited the relatively low HOMO level among 1–5, 1a, 1b, 4a, and 4b, which is correlated with the order of calculated *E*° value. The orbital lobes localized at the C5–C7 positions in the HOMOs of 1–3 showed that electron-donating groups at those positions may effectively elevate the HOMO level. In good agreement with this expectation, the HOMO level of 4 carrying three hydroxyl groups at all the C5, C6, and C7 positions was reached at −5.924 eV, which became lower (for 5) when an electron-withdrawing moiety was located at the C8 position. In addition, the HOMO levels of 1a and 4a were be relatively stabilized, compared to those of 1 and 4, by an inductive effect from the *meta*-positioned methoxy substituents.^58^ Moreover, the translocation of an electron-rich phenyl ring (B ring) from *δ*^+^-charged β-carbon (C2) to *δ*^−^-charged α-carbon (C3) offered the higher HOMO levels of 1b and 4b due to the unfavorable π–conjugation on the C ring enone, relative to 1 and 4, respectively.

To determine the ability of 1–5, 1a, 1b, 4a, and 4b to quench free organic radicals, we performed the Trolox equivalent antioxidant capacity (TEAC) assay employing the cationic radical form of 2,2′-azino-bis(3-ethylbenzthiazoline-6-sulphonic acid).^59^ As presented in Fig. 2a and c, flavonoids were tested, except for 1 that has limited solubility in ethanol (EtOH) under our experimental conditions. Flavonoids 2–5 and 4b showed the TEAC values close or over 1.0, relative to a vitamin E analog Trolox, suggesting that their radical scavenging ability is similar or greater than that of Trolox. As expected from the calculated redox potentials shown in Fig. 2a, 1a, 1b, and 4a presented no significant radical scavenging activity, relative to that of Trolox. As anticipated from the calculated *E*° values, relatively low TEAC values were observed for 2 and 3 over 4 and 4b. This investigation manifests the significance of the number of hydroxyl groups on the A ring towards redox properties and antioxidant activity. Despite three hydroxyl groups on the A ring, 5 with an electron-withdrawing chloro group displayed a similar TEAC value with 2 and 3 containing two hydroxyl groups. Our computational and experimental studies confirm that the isoflavone 4b embodying three hydroxyl groups without an electron-withdrawing substituent on the A ring can has the lowest redox potential among 1–5, 1a, 1b, 4a, and 4b, with the consequent noticeable radical scavenging ability. Moreover, the prooxidative activity of 2–5 and 4b that exhibit a notable radical scavenging ability were further analyzed by a cell-based fluorescent ROS detection assay employing human neuroblastoma SH-SY5Y (5Y) cells. As presented in Fig. S3,† no elevation of the cellular ROS concentration upon incubation with 0.01 and 0.1 mM of 2–5 and 4b was observed; however, as the increase of their concentrations, the prooxidative activity was observed at 0.5 and 1 mM. Such alteration in the antioxidant or prooxidant activity of flavonoids depending on their concentrations was reported in previous studies.^37,38^ Collectively, these results suggest that flavonoids 2–5 and 4b effectively scavenge free radicals, but they have the prooxidant activity under certain conditions, *e.g.*, high concentrations.

### Inhibition against AChE

**Fig. 3:**
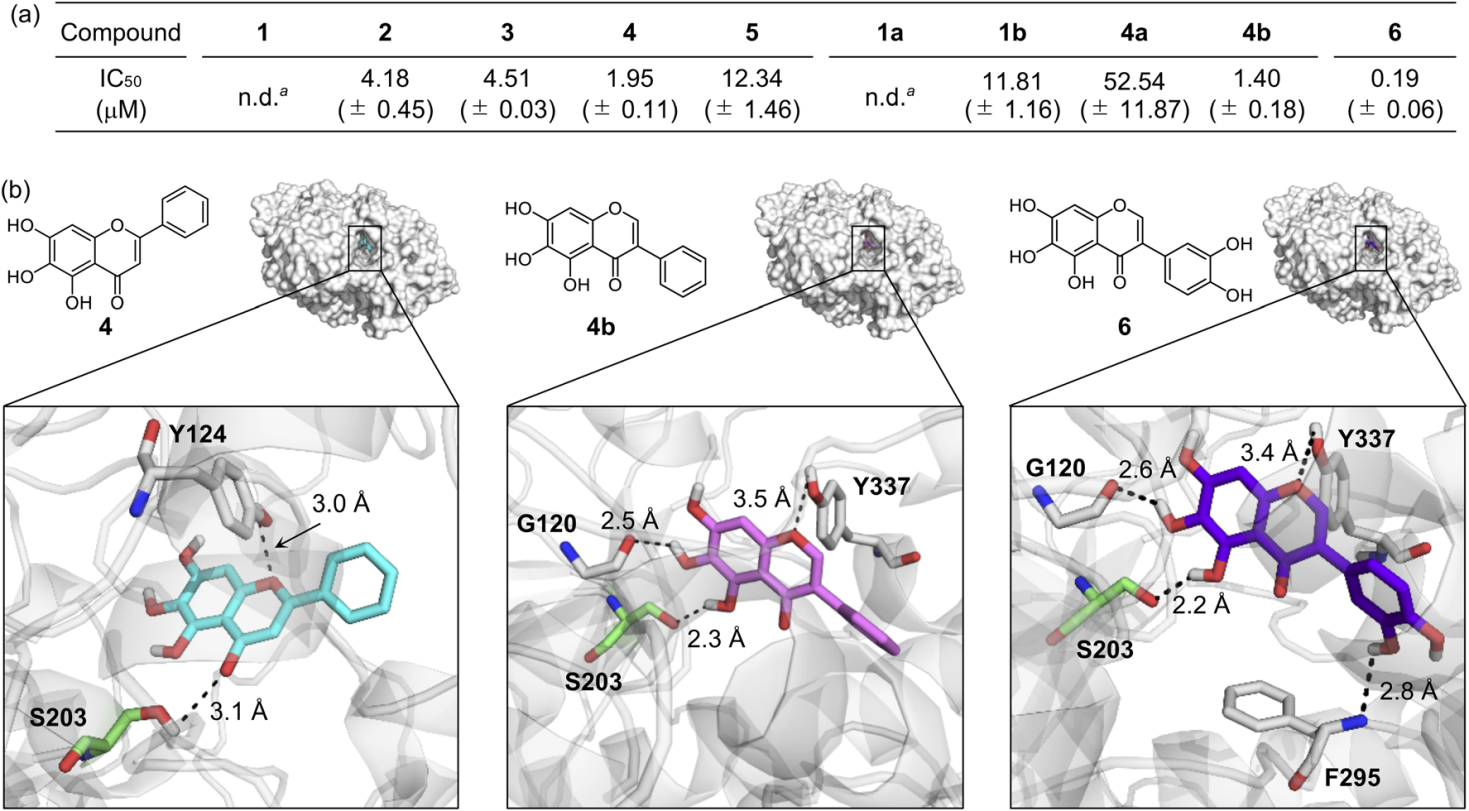
Inhibitory ability of flavonoids against AChE. (a) IC_50_ values of 1–5, 1a, 1b, 4a, 4b, and 6 for AChE inhibition determined by a fluorometric assay. ^*a*^n.d., not determined. Note that the IC_50_ values of 1 and 1a could not be obtained due to their low inhibitory activity against AChE. (b) Possible interactions of 4, 4b, and 6 with AChE (PDB 1C2B^61^) visualized by docking studies. Nine docked models of 4, 4b, and 6 against AChE with binding energies ranging from −10.8 to −8.7 kcal mol^−1^ were obtained. The representative models with the highest binding affinity towards AChE are shown in the figure. Hydrogen bonds between the flavonoids and AChE are indicated with dashed lines (2.2–3.5 Å). N, O, H, and C (from Ser203) atoms are depicted in blue, red, white, and green, respectively.

Inhibiting the activity of AChE can maintain a proper level of ACh under dysregulated cholinergic conditions.^10,11^ Thus, the inhibitory capability of flavonoids against AChE was investigated by a previously reported fluorescent AChE assay with slight modifications.^60^ As summarized in Fig. 3a, the IC_50_ values of flavonoids were in a micromolar range (IC_50_ = 1.40–52.54 μM). It should be noted that the IC_50_ values of 1 and 1a could not be obtained due to their negligible inhibitory activity against AChE under our experimental conditions. 4 and 4b that contain three hydroxyl groups on the A ring among our molecules exhibited relatively low IC_50_ values. The inclusion of three hydroxyl groups on the A ring is critical for controlling the catalytic activity of AChE, as previously reported,^49^ whereas the incorporation of a chloro substituent, with three hydroxyl groups, and methoxy moieties instead of hydroxyl groups on the A ring can decrease such inhibitory activity. Flavonoids 2, 3, and 1b over 1 illustrates the importance of the position of hydroxyl groups on the A ring and the isoflavone variation in directing the flavonoids' ability to inhibit the activity of AChE.

Docking studies were further conducted to visualize potential interactions between the flavonoids and AChE employing an X-ray crystal structure of electrophorus electricus AChE (PDB 1C2B^61^). As displayed in Fig. 3b and S4,† the flavonoids can interact with neighboring amino acid residues that lie in the active site of AChE *via* hydrogen bonding. For example, hydroxyl groups on the A ring could serve as a hydrogen-bond donor to interact with the backbone amide group between Gly121 and Gly122 (for 1 and 2), the backbone carbonyl moiety of Gly120–Gly121 (for 3 and 4b), and the hydroxyl group of Ser203 (for 4b) in AChE. In addition, hydrogen bonds mediated by the central O donor atom and the 4-oxo functionality on the C ring with Tyr337/Tyr341 (for 1, 2, and 1a), Gly122/Tyr124 (for 3), Tyr124/Ser203 (for 4), Tyr124 (for 5), Tyr124/Tyr337 (for 1b), Tyr124 (for 4a), and Tyr337 (for 4b) were also monitored. Anchoring the flavonoids into the cavity of the active site of AChE could prevent the substrate binding and, ultimately, inhibit the activity of AChE. In particular, hydrogen bonds between Ser203 at the catalytic triad and the 4-oxo functionality on the C ring of 4 or the hydroxyl group on the A ring of 4b were indicated within 3.1 Å and 2.3 Å, respectively. The Ser203 residue is responsible for initiating the hydrolysis of ACh through hydrogen bonding;^62^ thus, the intermolecular interaction with Ser203 could dominantly restrict ACh from binding into the active site of AChE. Overall, these observations highlight the pertinent role of some structural features, such as hydroxyl groups on the A ring and the C ring that contains two O donor atoms, in controlling the catalytic activity of AChE. It should be noted that the π–π stacking between the flavonoids' chromone moiety (A and C rings) and amino acid residues at the anionic subsite of AChE could afford the stability for their positioning at the cavity of AChE's active site,^46^ but our docking studies did not visualize these π–π stacking interactions.

### Effects on Aβ aggregation in the absence and presence of metal ions

**Fig. 4:**
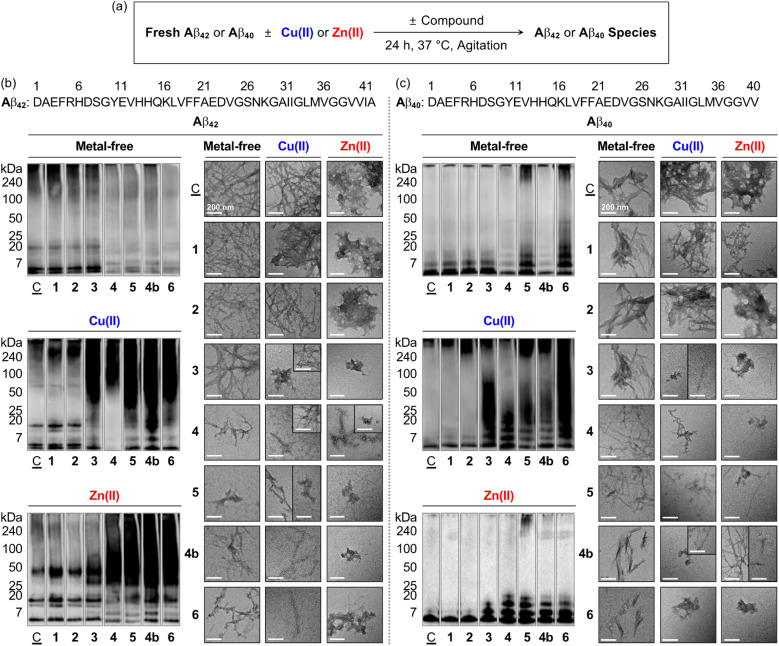
Effects of 1–6 and 4b on the formation of metal-free Aβ and metal–Aβ aggregates. (a) Scheme of the inhibition experiments. MW distributions and morphologies of the resultant (b) Aβ_42_ and (c) Aβ_40_ species were analyzed by gel/western blot with an anti-Aβ antibody (6E10) and TEM, respectively. Lanes: (C̲) Aβ ± Cu(ii) or Zn(ii); (1) C̲ + 1; (2) C̲ + 2; (3) C̲ + 3; (4) C̲ + 4; (5) C̲ + 5; (4b); C̲ + 4b; (6) C̲ + 6. The original gel images are shown in Fig. S9a.† Conditions: [Aβ] = 25 μM; [M(II)] = 25 μM; [flavonoid] = 50 μM (1% v/v DMSO); 20 mM HEPES, pH 7.4, 150 mM NaCl; 37 °C; 24 h; constant agitation. Scale bars = 200 nm.

To verify whether a series of nine flavonoids can modulate the aggregation of Aβ with and without metal ions, the molecular weight (MW) distribution and morphology of the resultant Aβ species were determined by gel electrophoresis with western blotting (gel/western blot) using an anti-Aβ antibody (6E10) and transmission electron microscopy (TEM), respectively. Two types of experiments were carried out employing two major Aβ isoforms, *i.e.*, Aβ_42_ and Aβ_40_:^25^ (i) inhibition studies to identify the effect of 1–5, 1a, 1b, 4a, and 4b on the formation of Aβ aggregates; (ii) disaggregation studies to evaluate if the flavonoids with reactivities towards Aβ aggregation in inhibition studies can disassemble preformed Aβ aggregates or modulate their further aggregation, as illustrated in Fig. 4 and S5–S9.† Moreover, the solutions containing the flavonoids and Cu(ii) were probed by electronic absorption (Abs) spectroscopy to monitor their metal binding. As depicted in Fig. S10,† when 1–5, 1b, and 4b were incubated with 1 equiv. of Cu(ii), optical shifts corresponding to Cu(ii) binding to the O donor atoms, *e.g.*, the hydroxyl groups at the C5–C7 positions and the 4-oxo functionality on the A and C rings of the flavonoids, respectively (Fig. 1c). As expected, no Abs change of 1a and 4a composed of methoxy groups on the A ring was detected by addition of Cu(ii).

As presented in Fig. 4b, S5a, and S9a,† the inhibition experiments with metal-free Aβ_42_ showed the notably varied MW distribution upon treatment of 4, 5, and 4b over 1–3, 1a, 1b, and 4a. Flavonoids 4, 5, and 4b discernably reduced the signal intensity of the bands corresponding to Aβ_42_ with the MWs from *ca.* 4–20 kDa and larger than *ca.* 100 kDa, while 1–3, 1a, 1b, and 4a could not change the size distribution of metal-free Aβ_42_. In the case of Cu(ii)–Aβ_42_ incubated with 3 over compound-untreated Cu(ii)–Aβ_42_ or Cu(ii)–Aβ_42_ added with 1, 2, 1a, 1b, and 4a, more intense smearing was monitored in the MW window larger than *ca.* 25 kDa. The sample of Cu(ii)–Aβ_42_ incubated with 4 displayed the enhanced level of dodecameric or larger Aβ_42_ aggregates (*ca.* ≥ 50 kDa), with the decreased intensity of the bands assigned to monomeric, dimeric, trimeric, and tetrameric Aβ_42_. The alteration of the size distribution of Cu(ii)–Aβ_42_ induced by 5 and 4b was similar to that by the addition of 3. Comparable to Cu(ii)–Aβ_42_, the size distribution of Zn(ii)–Aβ_42_ species was modified by 4, 5, and 4b, with the indication of smearing bands at *ca.* 25–240 kDa, while 3 gave rise to increased signal intensity between *ca*. 25–75 kDa. In contrast, a significant change in the MW distribution was not indicated by addition of 1, 2, 1a, 1b, and 4a.

TEM investigations further supported the flavonoids' reactivity towards the aggregation of Aβ_42_. As illustrated in Fig. 4b and S5b,† the resultant metal-free Aβ_42_ aggregates produced with 4, 5, and 4b exhibited noticeably different morphologies from compound-untreated Aβ_42_ aggregates, while little or no morphological alteration was observed from those incubated with 1–3, 1a, 1b, and 4a. Metal-free Aβ_42_ added with 4 generated shorter and thinner fibrils. Upon incubation of 5 and 4b with metal-free Aβ_42_ species, smaller fibrils and less structured aggregates were monitored than those obtained from compound-unadded metal-free Aβ_42_, which was supported by circular dichroism (CD) spectroscopic investigations. When metal-free Aβ_42_ was treated with 5 or 4b for 24 h, a decrease in the β-sheet population [for 5, 66.7 (±15.3)%; for 4b, 35.5 (±5.7)%] and increase in the random-coil feature [for 5, 19.7 (±15.5)%; for 4b, 53.8 (±5.9)%] were monitored, compared to those of the compound-untreated sample [β-sheet, 73.7 (±0.7)%; random coils, 1.5 (±1.5)%] (Fig. S11†). In the case of Cu(ii)–Aβ_42_, 3–5 and 4b could vary the morphology of the resultant aggregates. A mixture of amorphous and small fibrillary aggregates was detected upon incubation of 3 and 5 with Cu(ii)–Aβ_42_, relative to mature fibrils observed upon incubation of Cu(ii)–Aβ_42_ only. The samples of Cu(ii)–Aβ_42_ with 4 and 4b showed chopped and thin fibrils. The resultant aggregates of Zn(ii)–Aβ_42_ were notably different by treatment of 3–5 and 4b, compared to large amorphous species observed in the sample of Zn(ii)–Aβ_42_ only flavonoids 3 and 4b triggered the formation of small amorphous aggregates. The addition of 4 to Zn(ii)–Aβ_42_ led to the generation of chopped fibrils and amorphous assemblies. In the presence of 5, both amorphous and fibrillary Zn(ii)–Aβ_42_ aggregates were monitored. Flavonoids 1, 2, 1a, 1b, and 4a did not indicate any noticeable morphological changes of the resultant Cu(ii)–Aβ_42_ and Zn(ii)–Aβ_42_ aggregates.

Moreover, to investigate the effects of the flavonoids on the oligomerization and fibrilization of metal-free Aβ_42_ and metal–Aβ_42_, we performed the dot blot assay employing anti-oligomer (A11) and anti-fibril (OC) antibodies,^23^ as illustrated in Fig. S12.† It should be noted that the thioflavin-T (ThT) assay utilized for monitoring the formation of β-sheet-rich aggregates cannot be carried out under our experimental conditions since the absorption windows of 4, 5, and 4b interfere with the excitation and emission wavelengths of ThT (Fig. S13†). As expected from our gel/western blot and TEM studies (*vide supra*), A11- and OC-detectable immunofluorescent signals were not significantly varied by treatment of 1, 2, 1a, 1b, and 4a with metal-free Aβ_42_ and metal–Aβ_42_ following incubation, relative to those from compound-untreated samples (Fig. S12†). In contrast, the formation of Aβ oligomers and fibrils were reduced in the samples of metal-free Aβ_42_ treated with 4, 5, and 4b, following 24 h incubation. In the case of Cu(ii)–Aβ_42_ or Zn(ii)–Aβ_42_, 3–5 and 4b showed the gradually decreased signal intensities detected by A11 and OC in a distinctive manner, indicative of the modulatory ability of 3–5 and 4b against the oligomerization and fibrilization of metal–Aβ_42_.

The aggregation of metal-free Aβ_40_ and metal–Aβ_40_ could be modified by flavonoids in a different extent to that observed with metal-free and metal-added Aβ_42_. As illustrated in Fig. 4c, S6, and S9a,† under metal-free conditions, the signal intensity of the bands from *ca.* 7–20 kDa assigned to smaller oligomeric Aβ_40_ species was reduced by 4 and 4b, while 5 increased the band intensity associated with the species larger than *ca.* 7 kDa. When 3–5 and 4b were treated to Cu(ii)–Aβ_40_ and Zn(ii)–Aβ_40_, the resultant Aβ species indicated the diverse MW distribution in the range of *ca.* 7–240 kDa to different extents. As shown in TEM images, shorter and thinner fibrils were generated in the samples of metal-free Aβ_40_ with 4, 5, and 4b. For Cu(ii)–Aβ_40_, 5 formed chopped filamentous Cu(ii)–Aβ_40_ aggregates, and 3, 4, and 4b produced both amorphous and fibrillary aggregates. These morphological changes were similar to those of Zn(ii)–Aβ_40_ incubated with 3–5 and 4b generated a bunch of fibrillary species with chopped filamentous aggregates.

Moreover, the disaggregation experiments exhibited the effects of 1–5 and 4b on preformed Aβ_42_ and Aβ_40_ aggregates in the absence and presence of Cu(ii) and Zn(ii), as summarized in Fig. S7 and S9b.† Flavonoids 4, 5, and 4b could disassemble preformed metal-free Aβ and metal–Aβ aggregates. In the case of 3, the reactivity was shown only in the presence of metal ions. Flavonoids 1 and 2 less noticeably affected preformed metal-free and metal-treated Aβ aggregates. To probe the secondary nucleation of disassembled Aβ aggregates induced by the flavonoids on Aβ monomers, additional disaggregation experiments were conducted by incubating preformed aggregates of metal-free and metal-treated Aβ or their seeds with Aβ monomers. As shown in Fig. S8,†4, 5, and 4b exhibited notable variations in the MW distribution of both metal-free Aβ_42_ and metal–Aβ_42_ species, and 3 displayed the reactivity specifically towards metal–Aβ_42_ species. These findings suggest that the generated Aβ fragments by the flavonoids could suppress secondary nucleation. Collectively, our overall inhibition and disaggregation studies demonstrate that 4, 5, and 4b can modulate the aggregation of Aβ in the absence and presence of metal ions as well as disassemble preformed metal-free Aβ and metal–Aβ aggregates. In contrast, among the flavonoids containing two hydroxyl or two/three methoxy groups on the A ring, 3 exhibits the corresponding reactivities only towards metal-mediated Aβ aggregation pathways. These indications describe the significance of three hydroxyl groups on the A ring in the flavonoid structure to impact the aggregation of metal-free Aβ and metal–Aβ.

### Rational selection, redox potential, synthesis, and cytotoxicity of 6

**Fig. 5:**
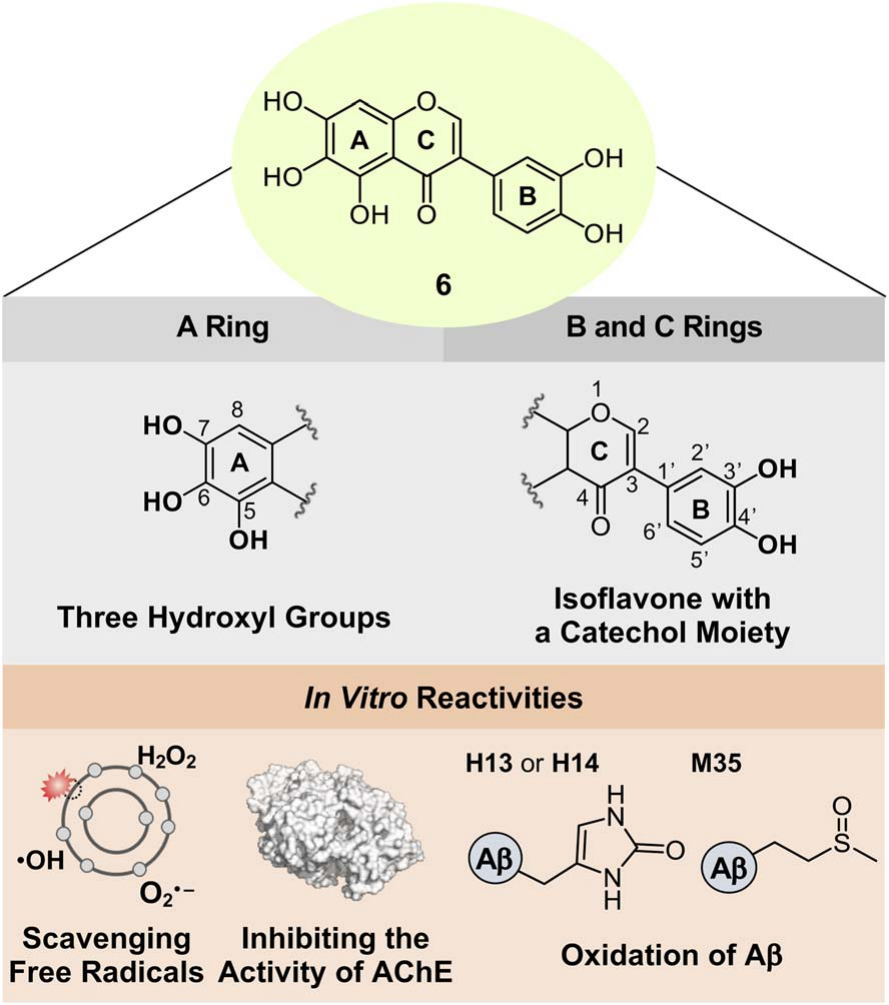
Design rationale of 6 and its reactivities against free radicals, AChE, metal-free Aβ, and metal–Aβ.

Based on the structure–activity relationship observed employing a series of nine flavonoids (Fig. 1c) with the previously reported studies of flavonoids,^46,48,49^ we could determine structural features on all the A, B, and C rings of flavonoids that are critical for controlling multiple pathological factors, *i.e.*, free radicals, AChE, metal-free Aβ, and metal–Aβ. As depicted in Fig. 5, first, three hydroxyl groups at the C5–C7 positions on the A ring are essential for gaining multiple reactivities with our desired pathogenic targets, relative to that of two hydroxyl or two/three methoxy groups. Second, the translocation of the B ring from C2 to C3 on the C ring, which produces an isoflavone framework, can enhance both free radical scavenging and AChE inhibitory activities. Third, a catechol moiety on the B ring in an isoflavone backbone is reported to be important in modulating the reactivities of our targets.^46^ In particular, a catechol group has been widely used for the design of chemical reagents (*e.g.*, small molecules and nanomaterials) as inhibitors against Aβ aggregation.^45,63–65^ Moreover, as illustrated in Fig. 2b, comparing the HOMOs of 4 and 4b indicates that the electronic distribution on all the A, B, and C rings is notable in the isoflavone 4b; thus, the addition of electron-donating groups at the C3′ and C4′ positions on the B ring can impact the electronic property of the overall structure. Collectively, we rationally fashioned the isoflavone 6 (Fig. 5) that contains three hydroxyl groups on the A ring and a catechol moiety on the B ring, as a flavonoid with multiple reactivities against pathological components in AD. As expected, 6 showed the most negative redox potential among all flavonoids, as shown in Fig. 2a. The addition of a catechol moiety on the B ring onto the isoflavone framework of 4b containing three hydroxyl groups on the A ring lowered the calculated redox potential from 1.202 to 1.018 V (*vs.* SHE). The orbital lobes localized between C3′ and C4′ in the HOMO of 6 can rationalize its more elevated HOMO level with the subsequent negative calculated *E*° value, compared to that of 4b, as displayed in Fig. 2a and b.

**Scheme 1:**
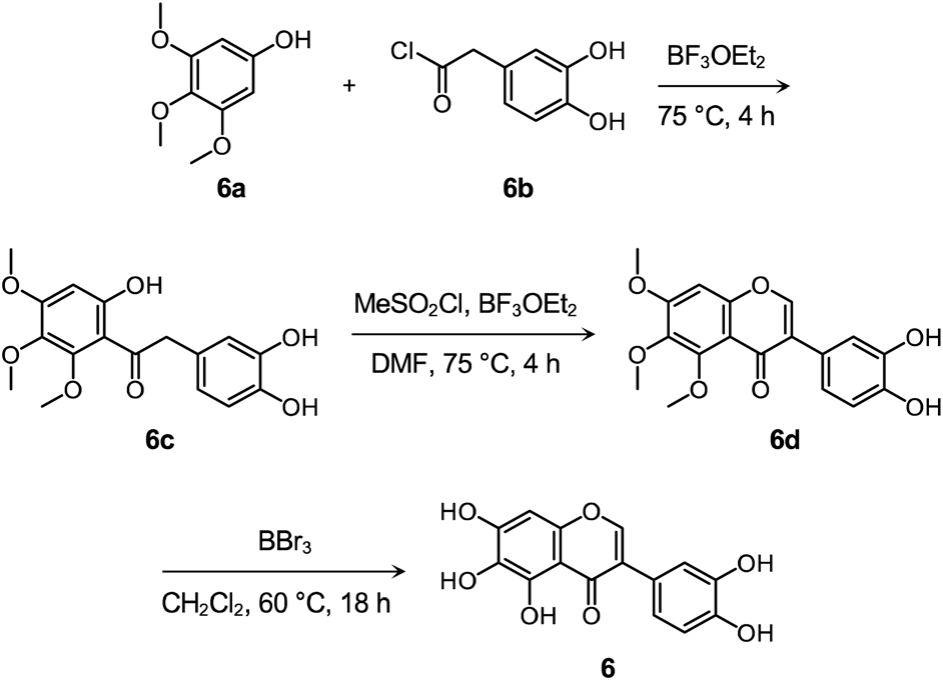
Synthetic routes to 6.

Flavonoid 6 was synthesized following previously reported procedures with modifications.^66–68^ As presented in Scheme 1 and Fig. S14–S16,† the Friedel–Crafts acylation of 6a with 6b afforded 6c in the presence of boron trifluoride ethyl etherate (BF_3_OEt_2_) as both the catalyst and solvent. Flavonoid 6d was prepared by the subsequent cyclization reaction of 6c using methanesulfonyl chloride (MeSO_2_Cl_2_), and the final product 6 was obtained *via* the demethylation reaction of 6d with boron tribromide (BBr_3_). After the preparation, the cytotoxicity of 6, compared to 1–5 and 4b, was determined by 3-(4,5-dimethylthiazol-2-yl)-2,5-diphenyltetrazolium bromide (MTT) assay employing human neuroblastoma 5Y cells. As summarized in Fig. S17,† greater than *ca.* 90% survival at up to 100 μM was observed upon the cells treated with 6 for 24 h. In contrast, the cells upon incubation with 100 μM of 1–5 and 4b indicated *ca*. 30%, 60%, 50%, 70%, 60%, and 70% viability, respectively, suggesting that 6 over 1–5 and 4b has relatively low cytotoxicity. It should be noted that cell viability was calculated, relative to that of the cells containing an equivalent amount of DMSO.

### Reactivities of 6 with free organic radicals, AChE, metal-free Aβ, and metal–Aβ

Flavonoid 6 exhibited the significant scavenging capability towards free organic radicals with the highest TEAC value [2.30 (±0.04)] among our flavonoid series, as shown in Fig. 2a and c. No prooxidative activity of 6 at up to 0.1 mM was observed; however, the cells upon incubation with 0.5 and 1 mM of the flavonoid indicated a increase in the amount of cellular ROS (Fig. S3†). These results imply that 6 serves as an antioxidant or prooxidant depending on experimental conditions. In addition, the most remarkable inhibition against the activity of AChE was observed by 6 with a nanomolar IC_50_ value (Fig. 3a). To visualize the potential interactions of 6 with AChE, docking studies were conducted. As illustrated in Fig. 3b, three and two hydroxyl groups on the A and B rings, respectively, and the central O donor atom on the C ring could have hydrogen bonds with the backbone carbonyl moiety of Gly120–Gly121, the hydroxyl group of Ser203 and Tyr337, and the backbone amide group between Ile294 and Phe295 within the cavity of AChE's active site. Especially, a hydrogen bond between the hydroxyl group at the C5 position on the A ring and Ser203 at the catalytic triad was indicated within 2.2 Å. Moreover, the number of hydrogen bonds between 6 and amino acid residues in the substrate-binding pocket of AChE was increased to four, as expected from the additional catechol functionality on the B ring, compared to that shown in 1–5, 1a, 1b, 4a, and 4b [one (for 4a and 5); two (for 1a, 1b, and 4); three (for 1–3 and 4b)]. These overall interactions can position 6 in the cavity of the active site and sterically halt the substrate ACh binding into the catalytic triad, which leads to its noticeable inhibitory ability against the activity of AChE.

Moving forward, 6 remarkably modified the oligomerization and fibrillization of both metal-free Aβ and metal–Aβ and affected their corresponding preformed Aβ aggregates and seeds, with its Cu(ii)-binding property, as illustrated in Fig. 4 and S7–S12.† In the inhibition experiments (gel/western blots), the intensity of the bands from the sample containing 6 and metal-free Aβ_42_ was reduced at *ca.* 4–20 kDa and above *ca.* 100 kDa, relative to that observed in the compound-free sample (Fig. 4b and S9a†). Furthermore, smearing bands from *ca.* 20–240 kDa were noticeably detected upon incubation of Cu(ii)–Aβ_42_ and Zn(ii)–Aβ_42_ with 6. TEM studies indicated shorter and thinner fibrils by adding 6 with metal-free Aβ_42_ than those of compound-untreated metal-free Aβ_42_. This result was supported by the CD analysis that indicated a change in the secondary structure of metal-free Aβ_42_ in the presence of 6, relative to that of the compound-free sample (Fig. S11†). The morphological changes of Cu(ii)–Aβ_42_ and Zn(ii)–Aβ_42_ aggregates were also prominent upon treatment of 6, exhibiting chopped fibrils and a mixture of amorphous and fibrillary aggregates, respectively (Fig. 4b). These reactivities of 6 were further supported by the dot blot assay, indicating significantly decreased A11- and OC-detectable signal intensities (Fig. S12†). The modulatory abilities of 6 were also observed in the inhibition studies with Aβ_40_ (Fig. 4c and S9a†) as well as the disaggregation experiments employing preformed metal-free and metal-bound Aβ_42_ and Aβ_40_ aggregates (Fig. S7, S8, and S9b†).

### Interaction of 6 towards Aβ

**Fig. 6:**
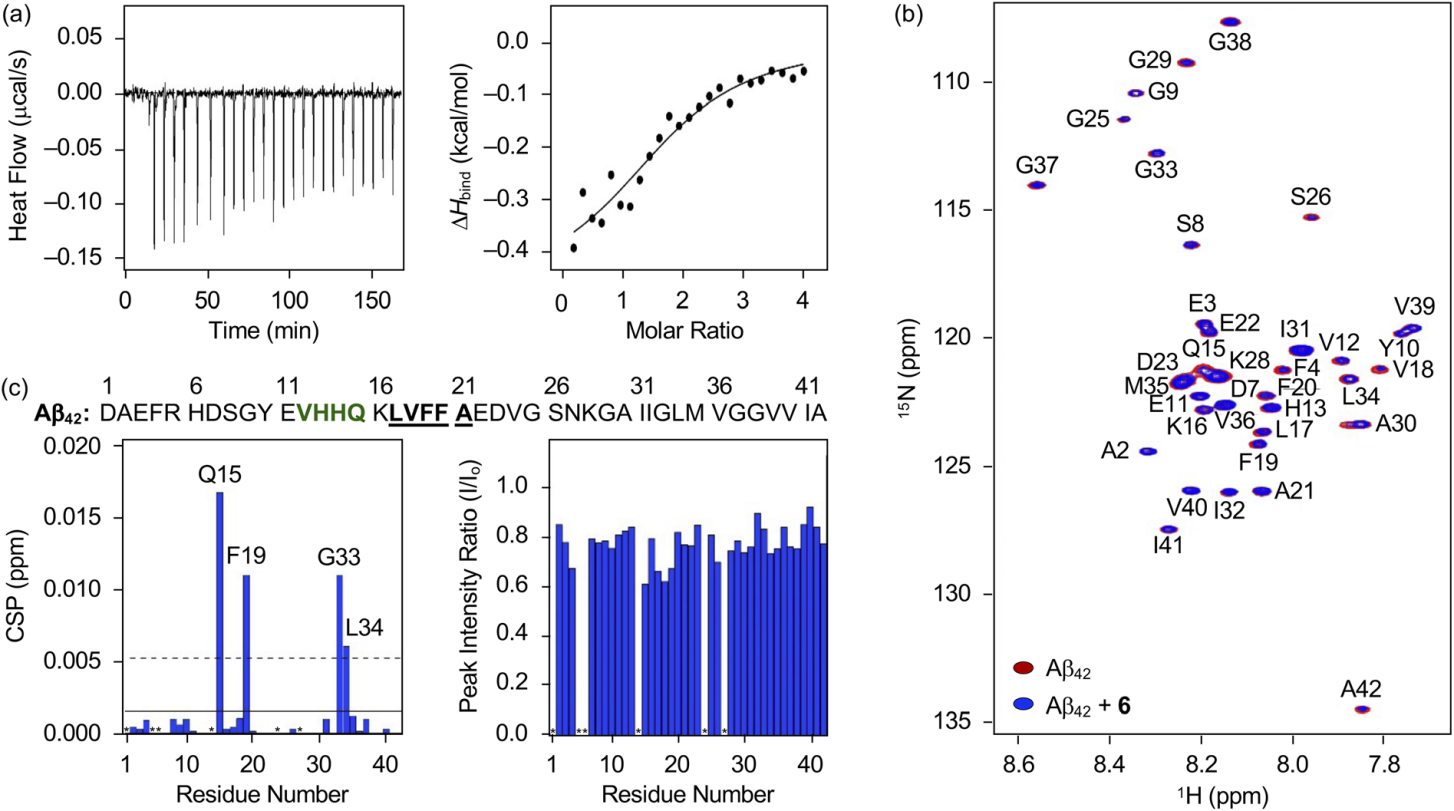
Interaction of 6 with monomeric Aβ_42_. (a) Binding of 6 with Aβ_42_ recorded by ITC. The ITC thermogram (left) and binding isotherm (right) of 6 with Aβ_42_ are depicted. The solid line indicates the best fit of the ITC data to a one-site binding model. Conditions: [Aβ_42_] = 40 μM; [6] = 800 μM (1% v/v DMSO); 20 mM HEPES, pH 7.4; 10 °C. (b) 2D ^1^H–^15^N SOFAST-HMQC NMR (800 MHz) spectra of ^15^N-labeled Aβ_42_ incubated with and without 6. (c) CSPs and peak intensity ratios of amino acid residues in ^15^N-labeled Aβ_42_ upon treatment of 6. Amino acid residues involved in the β-turn motif and the self-recognition site are highlighted in bold/green and bold/underline, respectively. The unresolved peaks (*i.e.*, Asp1, Arg5, His6, His14, Val24, and Asn27) are indicated with asterisks. Two horizontal lines indicate the average chemical shift (solid line) plus one standard deviation (dashed line). Conditions: [^15^N-labeled Aβ_42_] = 33 μM; [6] = 100 μM (1% v/v DMSO); 20 mM HEPES, pH 7.4; 10% v/v D_2_O; 10 °C.

Distinct reactivities of 6 with metal-free Aβ and metal–Aβ were supported by various biophysical approaches. As displayed in Fig. S18,† the chemical transformation of 6 triggered by metal-free Aβ_40_ or metal–Aβ_40_ was detected by Abs spectroscopy. In the absence of Aβ, an increase or decrease in the absorption of the peaks at *ca.* 265, 300, 340, and 410 nm was monitored for 24 h (Fig. S18†), which could be resulted from the oxidation of 6 in the presence of O_2_.^69^ These spectral changes were accelerated upon incubation with Aβ, indicative of its impact on the oxidative modification of 6. Such transformation of 6 was also indicated in the presence of Cu(ii) or Zn(ii) with and without Aβ. To probe the interaction of 6 with Aβ, their binding properties were analyzed by isothermal titration calorimetry (ITC). For the association between 6 and Aβ, we recorded the heat change upon constant titration of the flavonoid into the solution of Aβ, where the dilution heat was also measured to avoid the heat change induced by its addition into the buffered solution. The complex of 6 and Aβ_42_ or Aβ_40_ was formed spontaneously and exothermically [for Aβ_42_, Δ*G* = −6.1 (±0.1) kcal mol^−1^; Δ*H* = −0.5 (±0.1) kcal mol^−1^; *T*Δ*S* = 5.6 (±0.1) kcal mol^−1^; for Aβ_40_, Δ*G* = −6.0 (±0.5) kcal mol^−1^; Δ*H* = −0.6 (±0.5) kcal mol^−1^; *T*Δ*S* = 5.4 (±0.7) kcal mol^−1^], which was driven by both negative enthalpy and positive entropy changes, as summarized in Fig. 6a and S19a.† Binding affinities of 6 towards Aβ_42_ and Aβ_40_ were determined to be *ca.* 19.5 (±0.7) and 20.5 (±16.0) μM, respectively. Thus, 6 can bind to Aβ with a micromolar binding affinity.

The amino acid residues in Aβ affected upon interaction with 6 were further investigated by two-dimensional band selective optimized flip-angle short transient-heteronuclear multiple quantum correlation nuclear magnetic resonance (2D SOFAST-HMQC NMR) spectroscopy employing both ^15^N-labeled Aβ_42_ and Aβ_40_. As presented in Fig. 6b and c, the chemical shift perturbations (CSPs) provoked by adding 6 to uniformly ^15^N-labeled Aβ_42_ were detected. The Gln15, Phe19, Gly33, and Leu34 residues in Aβ_42_ were changed by treatment of 6, which implies that the flavonoid could affect the amino acid residues adjacent to the β-turn motif (Val12–Gln15), self-recognition site (Leu17–Ala21), and C-terminal region (Ile32–Ala42) of Aβ_42_ that are highly linked to its fibrilization.^9,70–73^ Therefore, this interaction between 6 and Aβ_42_ could cause a change in the conformation and aggregation propensity of Aβ_42_. Moreover, the overall peak intensity of ^15^N-labeled Aβ_42_ was decreased by *ca.* 20% upon addition of 6, suggesting the production of NMR-invisible Aβ aggregates.^74^ In the case of Aβ_40_, 2D SOFAST-HMQC NMR spectra obtained from ^15^N-labeled Aβ_40_ with and without 6 manifested the flavonoid's plausible interactions with Glu3, Glu11, Phe20, and Leu34 with the reduced overall peak intensity (*ca*. 10%), as depicted Fig. S19b and c.† In a similar manner to Aβ_42_, 6 could alter the aggregation of Aβ_40_ by interacting with the aforementioned regions, including the self-recognition (Leu17–Ala21) and C-terminal (Ile32–Val40) sites.^9,70–73^

### Aβ oxidation by 6

**Fig. 7:**
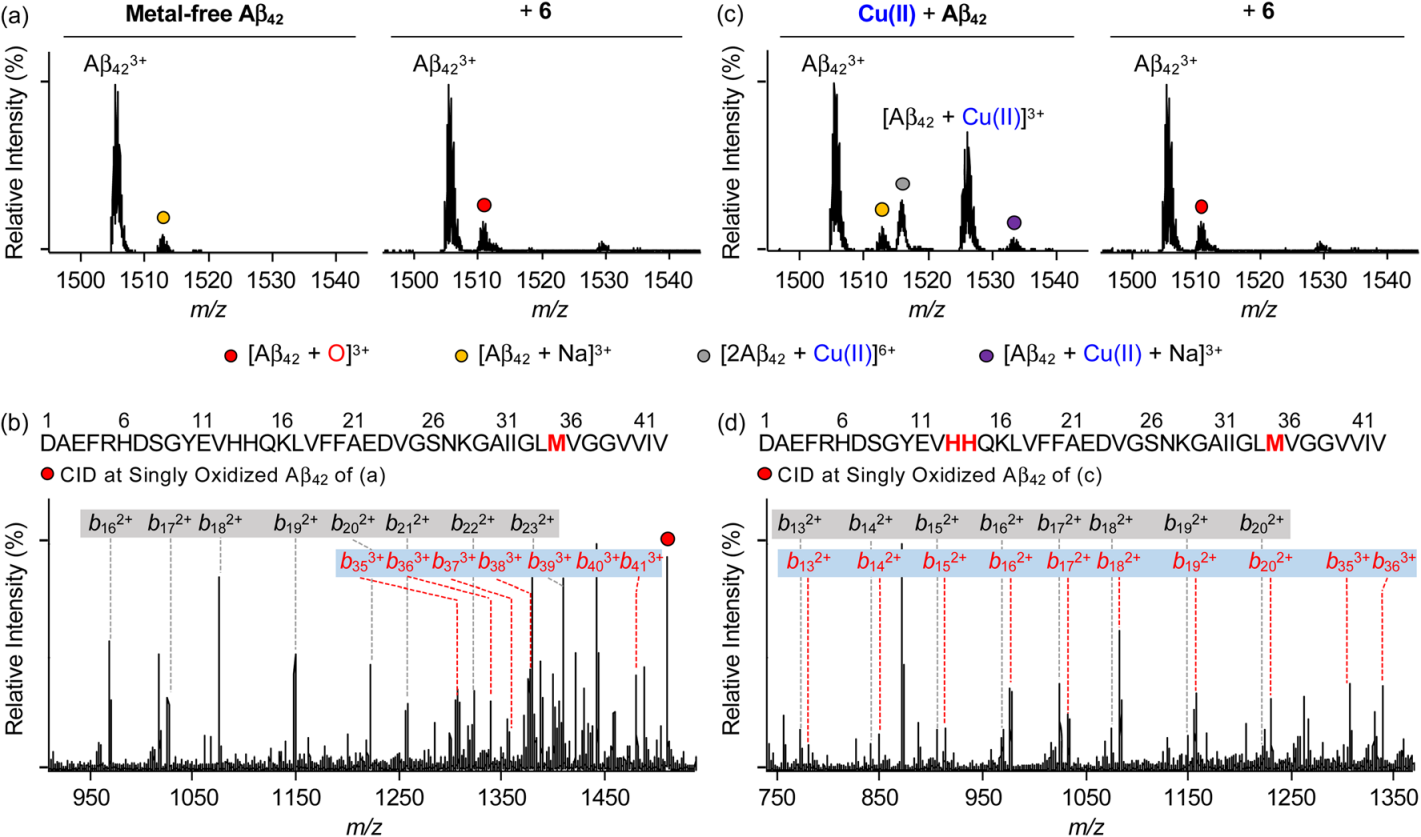
Analysis of metal-free and Cu(ii)-treated Aβ_42_ upon addition of 6 by ESI-MS and ESI-MS^2^. ESI-MS spectra of +3-charged Aβ_42_ and ESI-MS^2^ analyses of the singly oxidized Aβ_42_ (1511 *m*/*z*) were obtained upon incubation with 6 in the (a and b) absence and (c and d) presence of Cu(ii). The peaks highlighted as a red circle correspond to the singly oxidized Aβ_42_. Detailed analysis of tandem spectra is summarized in Fig. S21.† Conditions: [Aβ_42_] = 100 μM; [Cu(ii)] = 100 μM; [6] = 200 μM (1% v/v DMSO); 20 mM ammonium acetate, pH 7.4; 37 °C; 3 h; constant agitation. The samples were diluted by 10-fold with H_2_O before injection into the mass spectrometer.

To identify the modifications of Aβ in both the absence and presence of Cu(ii) upon treatment of 6, we carried out the studies employing electrospray ionization mass spectrometry (ESI-MS). In the sample containing metal-free Aβ_42_ and 6, the addition of +16 Da into the +3-charged Aβ_42_ monomer, assigned to be the oxidized monomeric Aβ_42_, was detected (Fig. 7a), while such oxidation was not observed under anaerobic conditions (Fig. S20†). To determine the oxidized amino acid residues in Aβ_42_, the *b* fragments generated by collision-induced dissociation were analyzed by tandem MS (ESI-MS^2^). As illustrated in Fig. 7b and S21a,† the Met35 residue was measured to be the oxidation site in the singly oxidized Aβ_42_. The previous studies suggested that the His, Tyr, and Met residues in Aβ are susceptible to be oxidized and such oxidation could impact the aggregation profile of Aβ.^75–78^ In particular, the oxidation of the Met residue to Met sulfoxide or sulfone can increase its polarity leading to the destabilization of β-strand structures and the decrease in hydrophobic contacts onto the C-terminal region that are associated with amyloid aggregation.^76,78^ Similar to metal-free Aβ_42_, the oxidation of Aβ_40_ at the Met35 residue was monitored by incubation with 6 (Fig. S22a, b and S23a†).

Aβ was oxidatively modified by incubation of Cu(ii)–Aβ with 6 in the presence of O_2_ in a distinct manner from metal-free Aβ treated with 6 (Fig. 7 and S20–S23†). As shown in Fig. 7c and S22c,† the intensity of the peaks assigned to Cu(ii)–Aβ_42_ and Cu(ii)–Aβ_40_ was suppressed. When collisional energy was selectively applied to the singly oxidized Aβ produced by reacting Cu(ii)–Aβ with 6, the analysis of *b* fragments indicated the His13, His14, and Met35 residues as plausible oxidation sites, as illustrated in Fig. 7d, S21b, S22d, and S23b.† In the case of His residues, their imidazole ring can involve in several intermolecular interactions, such as hydrogen bonding and π–π stacking, as the hydrogen-bond donor/acceptor and an aromatic π-motif.^79^ In addition, depending on pH, protonated His at both ε- and δ-nitrogen can mediate in the cation–π interactions with aromatic amino acid residues (*e.g.*, Tyr and Phe) as well as the formation of an intramolecular salt bridge between the carboxylate group of Glu22 and the protonated His13 or His14.^79–81^ Furthermore, His residues (*i.e.*, His6, His13, and His14) are included in metal coordination;^5,82^ thus, the transformation of His to 2-oxo-His could disrupt intermolecular and intramolecular interactions within Aβ and its metal binding.^9,81,83^ It should be noted that Cu(ii) chelation by 6 can impact metal-binding properties of Aβ.^45^ Therefore, oxidative modifications onto His13, His14, and Met35 by 6 could modify the aggregation of Cu(ii)–Aβ. Together, our overall biophysical results and observations demonstrate the direct interactions of 6 with metal-free and metal-treated Aβ species, with the consequent modulatory effects on their aggregation.

## Conclusions

AD is a complex condition, with multiple pathological factors contributing to its progression. This study presents a novel approach to developing flavonoids that can simultaneously target and regulate these factors. We conducted a series of experiments to identify key structural features for flavonoids to effectively control pathological targets and established the relationship between structure and activity. A series of nine flavonoids (1–5, 1a, 1b, 4a, and 4b) was rationally selected by adjusting the number and position of functional groups on the A ring and the position of the B ring, and their regulatory abilities against multiple pathogenic targets, including free radicals, AChE, metal-free Aβ, and metal–Aβ, were evaluated to establish the structure–activity relationship. Our investigations revealed three hydroxyl groups on the A ring as a critical structure feature to noticeably quench free organic radicals, inhibit the activity of AChE, and modulate the aggregation of metal-free Aβ and metal–Aβ. According to the structure–activity relationships obtained from this work (for the A ring) and the previous studies (for the B and C rings),^46^ we strategically fashioned the isoflavone 6 possessing three hydroxyl groups on the A ring and a catechol moiety on the B ring. Multiple reactivities of 6 against our desired targets are summarized: (i) scavenging free organic radicals exhibiting the highest TEAC value among our series of flavonoids; (ii) inhibiting the catalytic activity of AChE with a nanomolar IC_50_ value; (iii) modifying the formation of metal-free Aβ and metal–Aβ aggregates; (iv) disassembling preformed Aβ aggregates and altering their further aggregation with and without metal ions. Moreover, our computational and biophysical studies illuminated mechanistic details of 6's versatile reactivities towards free organic radicals, AChE, metal-free Aβ, and metal–Aβ. A relatively low redox potential and structure-based interactions with the cleft at the active site of AChE offer significant free radical scavenging and AChE inhibitory capabilities of 6, respectively. Oxidative modifications of metal-free and metal-added Aβ through direct interactions of 6 lead to altering their aggregation profiles. Our overall findings highlight a multidisciplinary strategic approach for designing multi-target-directed flavonoids as chemical reagents for AD and serve as a roadmap for designing small molecules as chemical tools or therapeutic candidates in neurodegenerative disorders.

## Data availability

All experimental details and data supporting the findings of this study are available within the paper and its ESI.† The data are also available from the corresponding authors upon reasonable request.

## Author contributions

M. H. L. and S. P. designed the research. S. P. performed biochemical assays (TEAC assay, cellular ROS assay, AChE assay, dot assay, and gel/western blot), spectroscopic measurements (absorbance and CD), ESI-MS, DFT calculation, and docking studies with data analysis. M. K. carried out TEM measurements, the dot assay, and cell studies. Y. L. and Y.-H. L. collected and analyzed ITC and 2D ^1^H–^15^N HMQC NMR data. M. H. contributed to DFT calculation. A. M. and G. N. synthesized 5 and 6, respectively. J. K. conducted the expression and purification of ^15^N-labeled recombinant Aβ_42_. S. P., M. K., M. H., and M. H. L. wrote the manuscript with input from all authors.

## Conflicts of interest

There are no conflicts to declare.

## Supplementary Material

SC-014-D3SC00752A-s001
