# Supplementary material for: Designing multi-target-directed flavonoids: a strategic approach to Alzheimer's disease
Source: Chem Sci. 2023 Aug 3;14(35):9293–305. doi: 10.1039/d3sc00752a (PMC10498667; doi:10.1039/d3sc00752a)
Supplement: SC-014-D3SC00752A-s001 [file SC-014-D3SC00752A-s001.pdf]

Electronic Supplementary Material (ESI) for Chemical Science.
This journal is © The Royal Society of Chemistry 2023

Supplementary Information

for

Designing multi-target-directed flavonoids: A strategic approach to Alzheimer's disease

Seongmin Park,[‡a] Mingeun Kim,[‡a] Yuxi Lin,[b] Mannkyu Hong,[ac] Geewoo Nam,[a] Adam Mieczkowski,[d] József Kardos,[e] Young-Ho Lee*[bfghi] and Mi Hee Lim*[a]

[a]Department of Chemistry, Korea Advanced Institute of Science and Technology (KAIST), Daejeon 34141, Republic of Korea

[b]Research Center for Bioconvergence Analysis, Korea Basic Science Institute (KBSI), Ochang, Chungbuk 28119, Republic of Korea

[c]Center for Catalytic Hydrocarbon Functionalizations, Institute for Basic Science (IBS), Daejeon 34141, Republic of Korea

[d]Institute of Biochemistry and Biophysics, Polish Academy of Sciences, Pawińskiego 5a, 02-106 Warsaw, Poland

[e]Department of Biochemistry, Institute of Biology, Eötvös Loránd University, Budapest 1117, Hungary

[f]Bio-Analytical Science, University of Science and Technology (UST), Daejeon 34113, Republic of Korea

[g]Graduate School of Analytical Science and Technology, Chungnam National University, Daejeon 34134, Republic of Korea

[h]Department of Systems Biotechnology, Chung-Ang University (CAU), Gyeonggi 17546, Republic of Korea

[i]Frontier Research Institute for Interdisciplinary Sciences (FRIS), Tohoku University, Sendai, Miyagi 980-8578, Japan

[‡]These authors contributed equally to this work.

*To whom correspondence should be addressed: miheelim@kaist.ac.kr and mr0505@kbsi.re.kr

Table of Contents

Experimental Section

Materials and Methods	S4
Calculation of Redox Potentials	S5
Trolox Equivalent Antioxidant Capacity (TEAC) Assay	S5
Cellular Reactive Oxygen Species (ROS) Detection Assay	S6
Acetylcholinesterase (AChE) Activity Assay	S6
Docking Studies	S7
Amyloid-β (Aβ) Aggregation Experiments	S7
Gel Electrophoresis with Western Blotting (Gel/Western Blot)	S8
Transmission Electron Microscopy (TEM)	S8
Cu(II)-binding Studies	S8
Circular Dichroism (CD) Spectroscopy	S8
Dot Blot Assay	S9
Electronic Absorption (Abs) Spectroscopy	S9
Synthesis of **6**	S9
Cell Viability Studies	S10
Isothermal Titration Calorimetry (ITC)	S11
Expression and Purification of ^{15}N-labeled Recombinant $A\beta_{42}$	S11
Two-Dimensional $^{1}H-^{15}N$ Band Selective Optimized Flip-angle Short Transient-Heteronuclear Multiple Quantum Correlation Nuclear Magnetic Resonance (2D $^{1}H-^{15}N$ SOFAST-HMQC NMR) Experiments	S12
Electrospray Ionization–Mass Spectrometry (ESI–MS)	S12

**Fig. S1**	^{1}H (400 MHz) and ^{13}C (100 MHz) NMR spectra of **5** in DMSO-d_6	S13
**Fig. S2**	^{1}H (400 MHz) and ^{13}C (100 MHz) NMR spectra of **4b** in DMSO-d_6	S14
**Fig. S3**	Prooxidant activity of **2–6** and **4b** in SH-SY5Y (5Y) cells	S15
**Fig. S4**	Possible interactions of flavonoids against AChE	S16
**Fig. S5**	Effects of **1**, **1a**, **1b**, **4**, **4a**, and **4b** on the formation of metal-free $A\beta_{42}$ and metal–$A\beta_{42}$ aggregates	S17
**Fig. S6**	Influence of **1**, **1a**, **1b**, **4**, **4a**, and **4b** on the formation of metal-free $A\beta_{40}$ and metal–$A\beta_{40}$ aggregates	S18
**Fig. S7**	Impact of **1–6** and **4b** on preformed metal-free Aβ and metal–Aβ aggregates	S19

Fig. S8 Effects of **1**–**6** and **4b** on preformed metal-free and metal-added Aβ_{42} aggregates and seeds in the presence of freshly prepared Aβ_{42} S20

Fig. S9 Original gel images of Figs. 4 and S7 S21

Fig. S10 Interaction of the flavonoids with Cu(II) monitored by Abs spectroscopy S22

Fig. S11 Change in the secondary structure of metal-free Aβ_{42} upon incubation with **5**, **4b**, or **6** S23

Fig. S12 Formation of metal-free Aβ_{42} and metal–Aβ_{42} oligomers and fibrils upon treatment of flavonoids monitored by the dot blot assay S24

Fig. S13 Absorbance spectra of **4**–**6** and **4b** S26

Fig. S14 ^{1}H (400 MHz) and ^{13}C (100 MHz) NMR spectra of **6c** in DMSO-d_6 S27

Fig. S15 ^{1}H (400 MHz) and ^{13}C (100 MHz) NMR spectra of **6d** in CD_3OD S28

Fig. S16 ^{1}H (400 MHz) and ^{13}C (100 MHz) NMR spectra of **6** in CD_3OD S29

Fig. S17 Cytotoxicity of **1**–**6** and **4b** in 5Y cells S30

Fig. S18 Chemical transformations of **6** S31

Fig. S19 Interaction of **6** with monomeric Aβ_{40} S32

Fig. S20 Interactions of **6** with metal-free and Cu(II)-added Aβ_{42} in the absence of O_2 S33

Fig. S21 Detailed analysis of ESI–MS^2 spectra shown in Fig. 7 S34

Fig. S22 Interactions of **6** with metal-free and Cu(II)-added Aβ_{40} S35

Fig. S23 Detailed analysis of ESI–MS^2 spectra presented in Fig. S22 S36

References S37

Appendix A. Cartesian coordinates of the optimized geometries S39

Appendix B. Vibrational frequencies of the optimized structures S47

Experimental Section

Materials and Methods. All chemical reagents were purchased from commercial suppliers and used as received unless otherwise stated. Chrysin (**1**) was obtained from TCI Chemicals (Tokyo, Japan). 6,7-Dihydroxyflavone (**2**) and 5,7-dihydroxyisoflavone (**1b**) were purchased from Carbosynth (Compton, UK). 5,6-Dihydroxyflavone (**3**) and baicalein (**4**) were gained from Alfa Aesar (Ward Hill, MA, USA). 5,7-Dimethoxyflavone (**1a**) and 5,6,7-Trimethoxyflavone (**4a**) were purchased from Sigma-Aldrich (St. Louis, MO, USA) and AA Blocks LLC (San Diego, CA, USA), respectively. 8-Chlorobaicalein (**5**) and 5,6,7-trihydroxyisoflavone (**4b**) were prepared following previously reported procedures.[1,2] $A\beta_{40}$ (DAEFRHDSGYEVHHQKLVFFAEDVGSNKGAIIGLMVG-GVV) and $A\beta_{42}$ (DAEFRHDSGYEVHHQKLVFFAEDVGSNKGAIIGLMVGGVVIA) were obtained from Peptide Institute, Inc. (Osaka, Japan). HEPES [2-(4-(2-hydroxyethyl)piperazin-1-yl)ethanesulfonic acid] was purchased from Sigma-Aldrich. The buffered solution was prepared in doubly distilled water [ddH_2O; a Milli-Q Direct 16 system (18.2 MΩ·cm; Merck KGaA)]. Trace metal contamination was removed from all solutions used for Aβ experiments by treating with Chelex (Sigma-Aldrich) overnight. Cellular ROS detection assay and AChE assay kits were purchased from Abcam (Cambridge, UK) and Invitrogen (Carlsbad, CA, USA), respectively. ^{1}H and ^{13}C NMR spectra of **5**, **4b**, and 3-(3,4-dihydroxyphenyl)-5,6,7-trihydroxy-4*H*-chromen-4-one (**6**) were recorded on a Bruker AV400 NMR spectrometer (Bruker BioSpin, Rheinstetten, Germany; Department of Chemistry, KAIST, Daejeon, Republic of Korea). High-resolution mass spectrometric analysis of **5**, **4b**, and **6** was carried out by micrOTOF-QII [Bruker Daltonics, Bremen, Germany; KAIST Analysis Center for Research Advancement (KARA), Daejeon, Republic of Korea]. Absorbance and fluorescence values for biological assays were measured on a Molecular Devices SpectraMax M5e microplate reader (Sunnyvale, CA, USA). Images gained through gel/Western blot were visualized by a ChemiDoc MP imaging system (Bio-Rad, Hercules, CA, USA). Morphologies of Aβ aggregates produced from the aggregation experiments were taken on a Tecnai F20 and G2 F30 S–Twin transmission electron microscope (FEI Company, Eindhoven, Netherlands; KARA). The change in the secondary structure of peptides was analyzed by a JASCO-815 150-L CD spectropolarimeter (Jasco Inc., Tokyo, Japan). Optical spectra were recorded on an Agilent 8453 UV−Vis spectrophotometer (Santa Clara, CA, USA). ITC measurements were performed by a VP–ITC instrument (Malvern Panalytical Ltd., Malvern, UK; KBSI, Ochang, Republic of Korea). 2D SOFAST-HMQC NMR spectra of uniformly ^{15}N-labeled $A\beta_{40}$ (rPeptide, Georgia, GA, USA) and $A\beta_{42}$ with and without flavonoids were collected by a Bruker AVANCE II-800 MHz NMR spectrometer equipped with a cryoprobe (Bruker BioSpin; KBSI). Mass spectrometric analysis of the interactions of **6** with $A\beta_{40}$ and $A\beta_{42}$ in the absence and

presence of Cu(II) was conducted by an Agilent 6530 Accurate-Mass and Waters Xevo G2-XS quadrupole time-of-flight liquid chromatography/mass spectrometry.

Calculation of Redox Potentials. All calculations were performed based on the density functional theory (DFT)[3] with the Jaguar 9.1 suite[4] at B3LYP[5–7] level of theory. The optimization of flavonoids' structures was carried out with the 6-31G** basis set.[8–10] Following the geometry optimization, electronic energies of the optimized flavonoid structures were recalculated with a high quality triple-ζ basis set cc-pVTZ(-f).[11] All atoms in the molecules were treated with the LACV3P** basis set, one of the classes in LACVP basis functions where the exponents were decontracted to match with the triple-ζ functions of the main group elements. Vibrational frequencies for the optimized structures were calculated at the same level of theory as the geometry optimization procedure. Vibrational entropy correction along with the zero-point vibrational energies was considered for proper thermodynamic approximations. Based on the optimized gas phase geometries, solvation correction energies were deduced. Self-consistent reaction field (SCRF)[12–14] approximations were considered to calculate the linearized Poisson-Boltzmann equations with the dielectric constant ε. The solvation energy used in the system was treated with EtOH (ε = 24.5). The Gibbs free energies in solution phase were computed as the following equations:

$$G(sol) = G(gas) + G(solv) \quad \text{(eq 1)}$$

$$G(gas) = H(gas) - TS(gas) \quad \text{(eq 2)}$$

$$H(gas) = E(SCF) + ZPE \quad \text{(eq 3)}$$

G(Sol) represents the Gibbs free energy with solvation correction G(solv) from the gas phase free energy G(gas); H(gas) is the enthalpy of the molecule in gas phase; T is the temperature (298.15 K); S(gas) is the entropy of the molecule in the gas phase; E(SCF) is the self-consistent field converged electronic energy; ZPE represents the vibrational zero-point energy. To calculate the redox potential, the free energy of the one-electron oxidized form of the flavonoid was deduced by the free energy of its neutral form.

TEAC Assay. The free radical scavenging capacity of flavonoids, relative to that of the vitamin E analog, Trolox, known as an antioxidant,[15] was determined by the TEAC assay based on the decolorization of ABTS [2,2'-azino-bis(3-ethylbenzothiazoline-6-sulfonic acid)diammonium salt] cation radicals. The TEAC assay was conducted in ethanol (EtOH) following previously reported

methods.[16,17] Blue $ABTS^{+•}$ cation radicals were generated by dissolving ABTS (7 mM) with potassium persulfate (2.5 mM) in H_2O (5 mL) and incubating the solution for 16 h at room temperature in the dark, and then the solution was diluted with EtOH to make the absorbance value of *ca*. 0.7 at 734 nm. The resultant $ABTS^{+•}$ solution (200 μL) was added into a clear 96-well plate. Various concentrations of flavonoids were added into a 96-well plate and incubated at 25 °C for 1, 3, and 6 min. The final concentrations of flavonoids were as follows: Trolox (0.24, 0.48, 0.97, 1.9, 3.9, 7.7, 16, 31, 62, 125, and 248 μM); **2** (0.10, 0.19, 0.39, 0.77, 1.6, 3.1, 6.2, 12, 25, 50, and 99 μM); **3** and **6** (0.39, 0.77, 1.6, 3.1, 6.2, 12, 25, 50, 99, 149, and 198 μM); **4** and **5** (0.077, 0.15, 0.31, 0.62, 1.2, 2.5, 5.0, 10, 15, 20, and 25 μM); **1a** and **1b** (0.03, 0.06, 0.12, 0.23, 0.47, 0.93, 1.9, 3.7, 7.4, 15, and 30 μM); **4a** (0.19, 0.39, 0.77, 1.6, 3.1, 6.2, 12, 19, 25, 37, and 50 μM); **4b** (0.019, 0.039, 0.077, 0.15, 0.31, 0.62, 1.2, 2.5, 5.0, 9.9, and 20 μM). Percent inhibition was calculated based on the measured absorbance at 734 nm [% inhibition = 100 x $(A_0 – A)/A_0$; A_0 = absorbance of the control well without flavonoids; A = absorbance of the wells treated with flavonoids] and plotted as a function of flavonoids' concentration. The TEAC values of each time point were calculated as the ratio between the slope of flavonoids and the slope of Trolox. All measurements were carried out in triplicate.

Cellular ROS Detection Assay. The prooxidant activity of flavonoids in living cells was measured by a cellular ROS detection assay kit (Abcam) following the manufacturer's protocol. The human neuroblastoma 5Y cells were maintained in media containing 50% v/v Dulbecco's Modified Eagle Medium and 50% v/v Nutrient Mixture F12 (phenol red-free DMEM/F12; GIBCO, Grand Island, NY, USA) supplemented with 10% v/v fetal bovine serum (GIBCO) and 100 U/mL penicillin-streptomycin (GIBCO). The cells were grown and maintained at 37 °C in a humidified atmosphere with 5% CO_2. Cells were seeded in a 96-well plate (50,000 cells/100 μL). After 24 h incubation, a ROS-staining working solution (100 μL) was added to each well and the plate was incubated for 1 h at 37 °C. Cells were then treated with the samples {[flavonoid] = 0.01, 0.1, 0.5, and 1 mM (1% v/v DMSO)}. The cellular ROS levels upon the treatment of samples were measured based on the fluorescence intensity ($\lambda_{ex}/\lambda_{em}$ = 520/605 nm) of the wells that was detected after 2 h incubation and calculated, relative to that of the cells containing an equivalent amount of DMSO. All experiments were performed in triplicate.

AChE Activity Assay. The AChE inhibitory activity of flavonoids was measured by a fluorometric AChE assay kit (Invitrogen) following the manufacturer's protocol with slight modifications.

Electric eel acetylcholinesterase (*ee*AChE) was dissolved and diluted with a working solution. The *ee*AChE solution (50 μL) was added into a 96-well plate. Various concentrations of flavonoids were then introduced into the 96-well plate. The final concentrations of flavonoids were as follow: **1**, **3**, **4**, **5**, **1a**, **1b**, **4a**, **4b**, and **6** (0.010, 0.50, 5.0, 50, and 250 μM); **2** (0.32, 1.6, 8, 40, and 200 μM). After shaking, the mixture of *ee*AChE and flavonoids was pre-incubated for 15 min. A reaction mixture containing acetylcholine, an AChE probe, and AbRed™ was added into the 96-well plate to initiate the reaction. The AChE activity was calculated based on the fluorescence intensity ($\lambda_{ex}/\lambda_{em}$ = 571/585 nm) of the wells that was detected after 15 min incubation. Data were normalized to the control (no inhibitor). All experiments were performed in triplicate.

Docking Studies. Flexible ligand docking studies using AutoDock Vina 1.5.6 software[18] for flavonoids were conducted against *ee*AChE (PDB 1C2B[19]). The MMFF94 energy minimization in ChemBio3D Ultra 11.0 was used to optimize the structure of flavonoids for docking studies. The structural files of flavonoids and the proteins were prepared in Auto Dock Tools,[20] imported into PyRx, and used to run AutoDock Vina.[18] The exhaustiveness for the docking runs was set at 1,024. Docked models of flavonoids with the proteins were visualized using Pymol 2.0.7.

Aβ Aggregation Experiments. Aβ was dissolved in ammonium hydroxide [1% w/w NH_4OH (aq)] and the resulting solution was aliquoted, lyophilized overnight, and stored at –80 °C. A stock solution of Aβ was then prepared by dissolving the lyophilized peptide with NH_4OH (1% w/w, aq; 10 μL) and diluting with ddH_2O. All Aβ samples were prepared following previously reported procedures.[21,22] The concentration of the peptide solution was determined by measuring its absorbance at 280 nm (ε = 1,450 $M^{-1}cm^{-1}$ for $A\beta_{40}$; ε = 1,490 $M^{-1}cm^{-1}$ for $A\beta_{42}$). The buffered solution (20 mM HEPES, pH 7.4, 150 mM NaCl) was used for preparing Aβ samples. For the inhibition studies, flavonoids (final concentration, 50 μM; 1% v/v DMSO) were added to the samples of Aβ (25 μM) in the absence and presence of $CuCl_2$ or $ZnCl_2$ (25 μM) followed by incubation for 24 h at 37 °C with constant agitation. For the disaggregation studies, Aβ (25 μM) was incubated with and without $CuCl_2$ or $ZnCl_2$ (25 μM) for 24 h at 37 °C with constant agitation to generate preformed Aβ aggregates. The resultant Aβ aggregates were then treated with flavonoids (50 μM) and incubated for additional 24 h with constant agitation. For the additional disaggregation experiments, preformed $A\beta_{42}$ aggregates (25 μM) or $A\beta_{42}$ seeds (10% v/v) with or without metal ions (25 μM for $CuCl_2$ and $ZnCl_2$) were incubated with flavonoids (50 μM) for 24 h with constant agitation. The resultant Aβ aggregates were then treated with freshly prepared $A\beta_{42}$

(25 μM) and incubated for additional 24 h with constant agitation.

Gel/Western Blot. The resultant Aβ species from the aggregation experiments were analyzed by gel/Western blot using an anti-Aβ antibody (6E10, Covance, Princeton, NJ, USA).[21,22] Each sample (10 μL) was separated on a 10–20% tricine gel (Invitrogen). Following separation, the proteins were transferred onto nitrocellulose membranes and blocked with bovine serum albumin (BSA, 3% w/v; Sigma-Aldrich) in Tris-buffered saline (TBS) containing 0.1% v/v Tween-20 (Sigma-Aldrich) (TBS-T) for 4 h at room temperature or overnight at 4 °C. The membranes were incubated with 6E10 (1:2,000) in the solution of BSA (2% w/v in TBS-T) for 4 h at room temperature. After washing with TBS-T (3x, 10 min), a horseradish peroxidase-conjugated goat anti-mouse secondary antibody (1:5,000 in 2% w/v BSA in TBS-T; Cayman Chemical Company, Ann Arbor, MI, USA) was added for 2 h at room temperature. A homemade ECL kit[21–23] was used to visualize gel/Western blots on a ChemiDoc MP Imaging System (Bio-Rad).

TEM. Samples for TEM were prepared following previously reported methods.[22,24] Glow-discharged grids (Formvar/Carbon 300-mesh; Electron Microscopy Sciences, Hatfield, PA, USA) were treated with Aβ samples (25 μM; 7.5 μL) for 2 min at room temperature. Excess sample was removed using filter paper followed by washing with ddH_2O (3x). Each grid incubated with uranyl acetate (1% w/v ddH_2O; 7.5 μL) for 2 min was blotted off and dried overnight at room temperature. Images for each sample were taken on a TEM (200 kV; 29,000x magnification; 300 kV; 29,500x magnification; KARA). The location of samples on the grids were randomly picked for taking more than 20 images per each grid.

Cu(II)-binding Studies. The interaction of flavonoids with Cu(II) was detected by Abs spectroscopy in 20 mM HEPES, pH 7.4, 150 mM NaCl. The solutions of flavonoids were titrated with 1 equiv of $CuCl_2$ at room temperature. The mixture solution was allowed to equilibrate for 5 min after the addition of $CuCl_2$ at room temperature before the spectra were recorded.

CD Spectroscopy. Metal-free $A\beta_{42}$ (100 μM) was incubated with and without **5**, **4b**, and **6** (200 μM) for 24 h in 20 mM HEPES, pH 7.4, 150 mM NaF at 37 °C with constant agitation (250 rpm). CD spectra were collected in the range from 195 nm to 255 nm. The digital integration time, the bandwidth, and the scanning speed were 4 s, 2 nm, and 20 nm/min, respectively. Each spectrum was smoothed by Fourier transforms. The change in the secondary structure of metal-free $A\beta_{42}$

treated with a flavonoid was analyzed following the previously reported BeStSel method.[25] The measurements were conducted in triplicate.

Dot Blot Assay. The samples containing Aβ_{42} (100 μM) with or without $CuCl_2$ or $ZnCl_2$ (100 μM) in the absence and presence of flavonoids (200 μM; 1% v/v DMSO) were prepared in 20 mM HEPES, pH 7.4, 150 mM NaCl and incubated for various time points (0, 1, 2, 3, 4, 6, 12, and 24 h). The resulting solution (2.5 μL) was spotted on a nitrocellulose membrane and blocked with a BSA solution (3% w/v) in 0.01% TBS-T at room temperature for 12 h. The membrane was incubated with a primary antibody [*i.e.*, 6E10[26] for Aβ species (1:2,000), A11[26] for Aβ oligomers (1:1,000; Invitrogen, Carlsbad, CA, USA), or OC[26] for Aβ fibrils (1:2,000; Merch Millipore, Billerica, MA, USA)] in the solution of BSA (2% w/v in TBS-T) for 4 h at room temperature. After washing with 0.01% TBS-T (3x, 7 min), the horseradish peroxidase-conjugated goat anti-mouse (1:5,000; for 6E10) or goat anti-rabbit (1:50,000; for A11 and OC) secondary antibody in the solution of BSA (2% w/v in TBS-T) was added to the membrane and incubated for 1 h at room temperature. A homemade ECL kit[21–23] was used to visualize the results on a ChemiDoc MP Imaging System. The intensity observed from A11- or OC-detectable Aβ species was quantified, relative to that detected from 6E10, by the ImageJ software.

Abs Spectroscopy. Flavonoid (50 μM; 1% v/v DMSO) was added into the buffered solution (20 mM HEPES, pH 7.4, 150 mM NaCl) with or without Aβ_{40} (25 μM) in the absence and presence of $CuCl_2$ or $ZnCl_2$ (25 μM). Abs spectra of the samples were collected for 24 h at 37 °C.

Synthesis of 6.

6c [3-(3,4-Dihydroxyphenyl)-5,6,7-trimethoxy-4*H*-chromen-4-one]. 3,4,5-Trimethoxyphenol (**6a**; 1.2 g, 6.3 mmol) and 3,4-dihydroxyphenylacetic acid (**6b**; 1.1 g, 6.3 mmol) were dissolved in boron trifluoride diethyl etherate ($BF_3{\cdot}OEt_2$, 10 mL) and refluxed at 75 °C. After 4 h, the reaction mixture was extracted in a solution of ethyl acetate (EtOAc) and saturated sodium acetate (NaOAc; 0.12 g/mL). The organic layer was collected, washed with brine, dried with sodium sulfate ($NaSO_4$), filtered, and concentrated. The crude product was purified by column chromatography (SiO_2; EtOAc:hexanes = 1:1) to yield **6c** [R_f = 0.3; yellow powder; 650 mg, 2.0 mmol (yield = 31%)] that was used for the next step without further purification. ^{1}H NMR [400 MHz, DMSO-d_6, δ (ppm)]: 6.58 (1H, d, J = 8.0 Hz), 6.54 (1H, dd, J = 8.0, 2.1 Hz), 6.40 (1H, d, J = 2.1 Hz), 6.29 (1H, s), 4.00 (2H, s), 3.77 (3H, s), 3.65 (3H, s), 3.62 (3H, s). ^{13}C NMR [100 MHz, DMSO-

d$_6$, δ (ppm)]: 202.9, 158.3, 158.0, 153.4, 144.9, 143.9, 134.4, 125.8, 120.5, 117.1, 114.5, 110.0, 96.2, 92.8, 61.3, 60.6, 56.0, 55.6, 48.6. HRMS: *m/z* Calcd. for $C_{17}H_{18}O_7$ [M – H]$^-$: 333.1053; found: 333.2388.

6d [3-(3,4-Dihydroxyphenyl)-5,6,7-trimethoxy-4*H*-chromen-4-one]. 6c (650 mg, 2.0 mmol) was dissolved in dry *N*,*N*-dimethylformamide (DMF, 8 mL), and $BF_3{\cdot}OEt_2$ (1.4 mL, 20 mmol) was added dropwise and heated to 50 °C for 1 h followed by adding methanesulfonyl chloride ($MeSO_2Cl$; 231 μL, 2.0 mmol) dropwise. The reaction mixture was refluxed at 100 °C for 2 h, cooled down to room temperature, and extracted with EtOAc containing saturated NaOAc (0.12 g/mL). The organic layer was collected, washed with brine, dried with $NaSO_4$, filtered, and concentrated. The crude product was purified by column chromatography (SiO_2; EtOAc:hexanes = 3:1). **7d** was obtained by washing the solid with CH_3OH [R_f = 0.4; yellow powder; 74 mg, 0.22 mmol (yield = 11%)]. ^{1}H NMR [400 MHz, CD_3OD, δ (ppm)]: 8.05 (1H, s), 7.03 (1H, d, *J* = 8.1 Hz), 6.97 (1H, dd, *J* = 8.1, 2.2 Hz), 6.83 (1H, d, *J* = 2.2 Hz), 3.99 (3H, s), 3.92 (3H, s), 3.87 (3H, s). ^{13}C NMR [100 MHz, CD_3OD, δ (ppm)]: 176.0, 158.4, 155.0, 152.4, 151.7, 145.3, 144.7, 140.4, 125.5, 123.4, 120.5, 116.3, 114.8, 112.7, 96.3, 61.1, 60.4, 55.6. HRMS: *m/z* Calcd. for $C_{18}H_{16}O_7Na$ [M + Na]$^+$: 367.0794; found: 367.0786.

6 [3-(3,4-Dihydroxyphenyl)-5,6,7-trihydroxy-4*H*-chromen-4-one]. 6d (74 mg, 0.22 mmol) was dissolved in dry dichloromethane (CH_2Cl_2) and stirred in an ice bath. A solution of boron tribromide (BBr_3) in CH_2Cl_2 (1 M, 250 μL) was added into the solution of **6d** dropwise and then the ice-bath was removed to stir the reaction mixture at 60 °C for 18 h followed by refluxing at 100 °C for 2 h. After cooling down to room temperature, the mixture was extracted with EtOAc and H_2O. The organic layer was collected, washed with brine, dried with $NaSO_4$, filtered, and concentrated. The crude was purified by column chromatography (SiO_2; CH_3OH:CH_2Cl_2 = 1:30). **6** was obtained by recrystallization using a mixture of acetone, hexanes, and CH_2Cl_2 [R_f = 0.2; white powder; 36 mg, 0.12 mmol (yield = 55%)]. ^{1}H NMR [400 MHz, CD_3OD, δ (ppm)]: 8.04 (1H, s), 7.02 (1H, d, *J* = 2.2 Hz), 6.86 (1H, dd, *J* = 2.2, 8.1 Hz), 6.83 (1H, d, *J* = 8.1 Hz), 6.46 (1H, s). ^{13}C NMR [100 MHz, CD_3OD, δ (ppm)]: 181.0, 153.5, 153.2, 151.0, 147.0, 145.3, 144.8, 129.1, 122.7, 122.6, 120.3, 116.1, 114.9, 105.1, 93.2. HRMS: *m/z* Calcd. for $C_{15}H_{10}O_7Na$ [M + Na]$^+$: 325.0324; found: 325.0317.

Cell Viability Studies. Human neuroblastoma 5Y cells were maintained in media containing 50% v/v minimum essential medium (MEM; GIBCO) and 50% v/v F12 (GIBCO) and supplemented with 10% v/v fetal bovine serum (Sigma-Aldrich) and 100 U/mL penicillin with 100 mg/mL streptomycin (GIBCO). The cells were grown and maintained at 37 °C in a humidified atmosphere with 5% CO_2.

Cell viability was determined by the MTT assay [MTT = 3-(4,5-dimethylthiazol-2-yl)-2,5-diphenyltetrazolium bromide]. Cells were seeded in a 96-well plate (15,000 cells/100 μL) and treated with the samples {[flavonoid] = 5, 10, 25, 50, and 100 μM (1% v/v DMSO)}. After 24 h incubation, 5Y cells were washed with phosphate buffered saline (PBS; GIBCO). MTT [5 mg/mL in PBS (pH 7.4, GIBCO); 25 μL] was added to each well and the plate was incubated for 4 h at 37 °C. The formazan produced by cells was solubilized using an acidic solution of DMF (pH 4.5, 50% v/v, aq) and sodium dodecyl sulfate (SDS; 20% w/v) overnight at room temperature in the dark. Absorbance was measured at 600 nm by a microplate reader (Molecular Devices). Cell viability, relative to that of the cells containing an equivalent amount of DMSO, was calculated. The measurements were conducted in triplicate.

ITC. The solutions of Aβ and **6** were subjected to degassing under vacuum for 3 min prior to loading into the instrument. The solution of **6** [800 μM; 20 mM HEPES, pH 7.4 (1% v/v DMSO, 1% w/w NH_4OH)] in the syringe was titrated into the solution containing Aβ [40 μM; 20 mM HEPES, pH 7.4 (1% v/v DMSO, 1% w/w NH_4OH)] in the cell at 10 °C with 25 injections at a constant interval of 360 s (2 μL for the first injection and 11 μL for the following injections). The cell was continuously stirred at 264 rpm. The initial delay and the reference power were 60 s and 10 μcal/s, respectively. Dilution heat of **6** only was also measured under the same experimental conditions. The ITC thermogram and binding isotherm were shown after the subtraction of the dilution heat. The binding isotherm after baseline correction was fitted to the one-set-of-sites binding model using the MicroCal Origin software (version 7.0).

Expression and Purification of ^{15}N-labeled Recombinant $A\beta_{42}$. ^{15}N-labeled recombinant $A\beta_{42}$ was expressed in *E. coli* and purified as previously reported.[27] Briefly, the DNA sequence coding the human $A\beta_{42}$ peptide was artificially constructed using codons preferred by *E. coli* with an extra Met residue at the *N*-terminal of the peptide. The DNA construct was inserted into pAED4 vector[28] and the *E. coli* BL21 (DE3) pLysS strain (Novagen, Inc., Madison, WI, USA) was utilized in the protein expression. Transformed cells were incubated in LB media containing ampicillin and chloramphenicol until the cultures reach 3.5 McFarland turbidity. Cells were then harvested by centrifugation and the cell pellets were resuspended in M9 minimal media containing isotopically labeled 1 g/L ^{15}N ammonium chloride, and induced by addition of 1 mM IPTG for 4 h. The peptide formed inclusion bodies in the bacterial cells. The purified inclusion bodies were dissolved in 20 mM NaOH and the peptide was further purified by repeated cycles of amyloid growth at low pH

and monomerization in hexafluoroisopropanol (HFIP) combined with centrifugation steps.

2D $^{1}H-^{15}N$ SOFAST-HMQC NMR Experiments. To prepare a stock solution of ^{15}N-labeled Aβ_{40} uniformly ^{15}N-labeled Aβ_{40} was dissolved in NH_4OH (1% w/w, aq) and the resulting solution was lyophilized for 48 h and stored at –80 °C. To remove possible preformed aggregates, the lyophilized peptide was then redissolved in diluted NaOH (pH 10) and passed through a filter (0.22 μm). On the other hand, the stock solution of ^{15}N-labeled Aβ_{42} based on the previous report.[27] Briefly, uniformly ^{15}N-labeled Aβ_{42} was solubilized in diluted NaOH (pH 10) at a concentration of ~200 μM. The undissolved precipitates of Aβ_{42} were removed by centrifugation at 40,000 rpm for 1 h. The solution of ^{15}N-labeled Aβ (33 μM) was prepared with and without **6** (100 μM; 1% v/v DMSO) in 20 mM HEPES, pH 7.4 (10% v/v D_2O) for NMR experiments. All spectra were acquired with 180 scans at 10 °C. Dimensions of the spectra were 1,024 (^{1}H) × 128 (^{15}N) points. Experimental data were processed with NMRpipe[29] and Sparky 3.115.[30] The ^{1}H and ^{15}N assignments of ^{15}N-labeled Aβ were determined based on the previous results.[21,31] The chemical shift perturbation was calculated following the equation (eq 4).[32] Note that $\Delta\delta_H$ and $\Delta\delta_N$ represent the alterations in the chemical shifts of ^{1}H and ^{15}N of ^{15}N-labeled Aβ, respectively.

$$\Delta\delta_{NH} = \sqrt{(\Delta\delta_H)^2 + \left(\frac{\Delta\delta_N}{6.5}\right)^2} \qquad \text{(eq 4)}$$

ESI−MS. Aβ (100 μM) was incubated with **6** (200 μM; 1% v/v DMSO) in the absence and presence of $CuCl_2$ (100 μM) in 20 mM ammonium acetate, pH 7.4 at 37 °C with constant agitation. The incubated samples were diluted by 10-fold with ddH_2O and then injected into the mass spectrometer. The capillary voltage, nozzle voltage, and gas temperature were set to 5.8 kV, 2 kV, and 300 °C, respectively. ESI–MS^2 analyses were additionally conducted on the singly oxidized Aβ produced by treating **6** with metal-free and Cu(II)-bound Aβ. ESI parameters and experimental conditions were the same as above. Collision-induced dissociation was carried out by applying the collision energy in the trap at 65 V. The isolation width was set as narrow (*ca*. 1.3 *m/z*), and more than 200 spectra were obtained for each sample and averaged for the analysis. For the experiments under anaerobic conditions, all samples were prepared following the same procedure described above for the aerobic samples in a N_2-filled glove box.

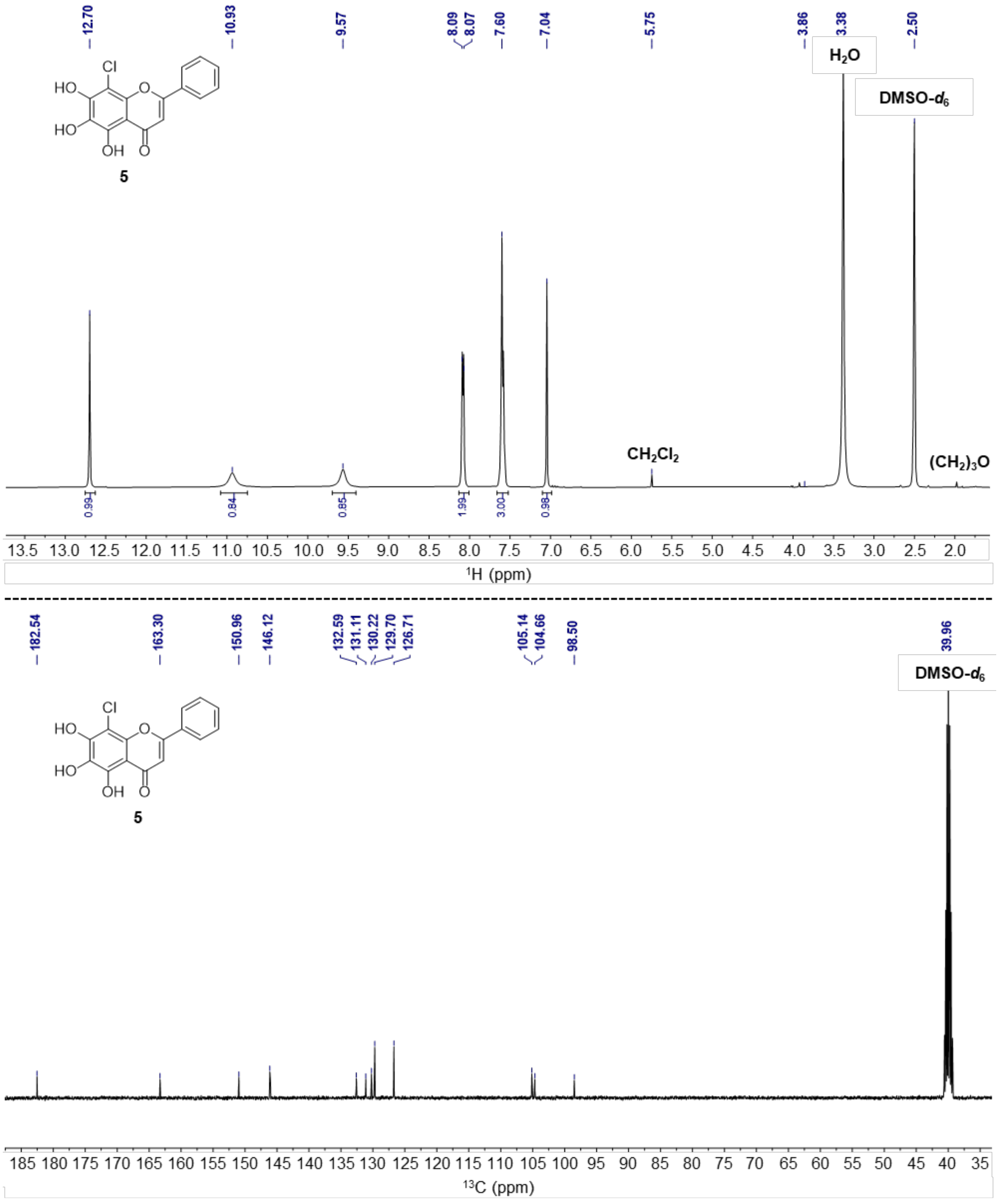

Fig. S1 ^{1}H (400 MHz) and ^{13}C (100 MHz) NMR spectra of **5** in DMSO-d_6.

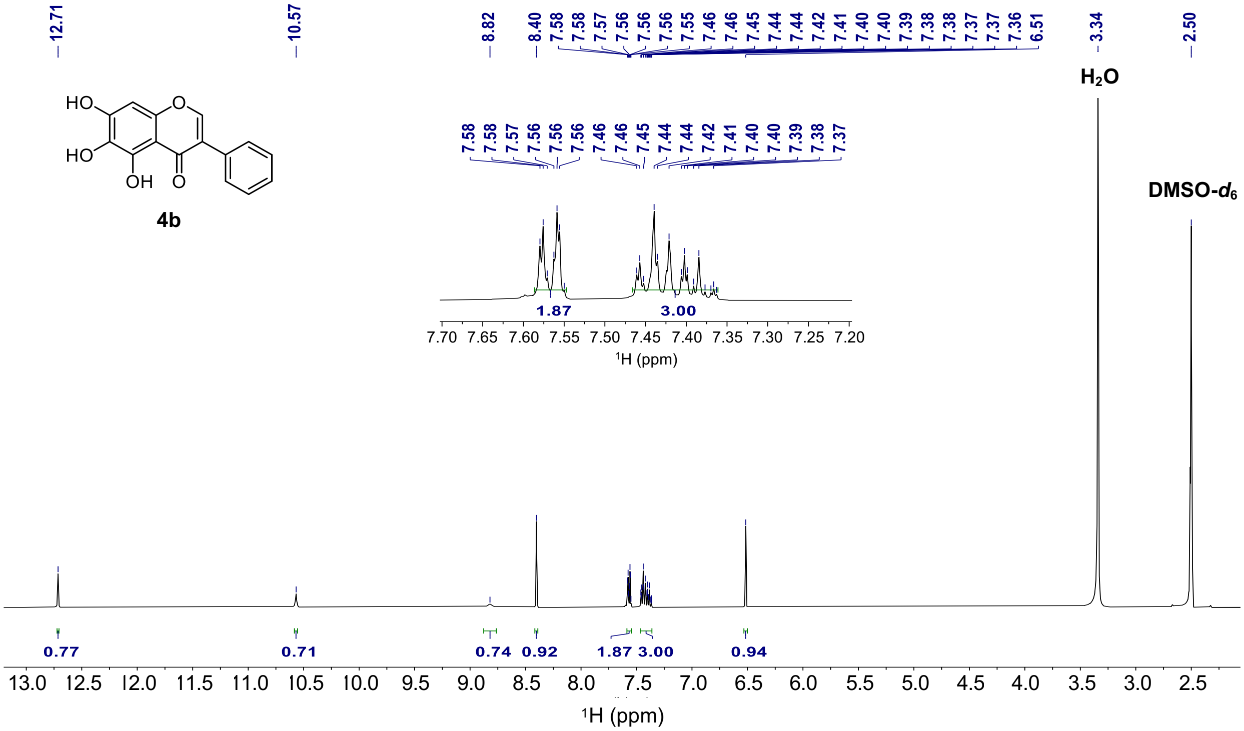

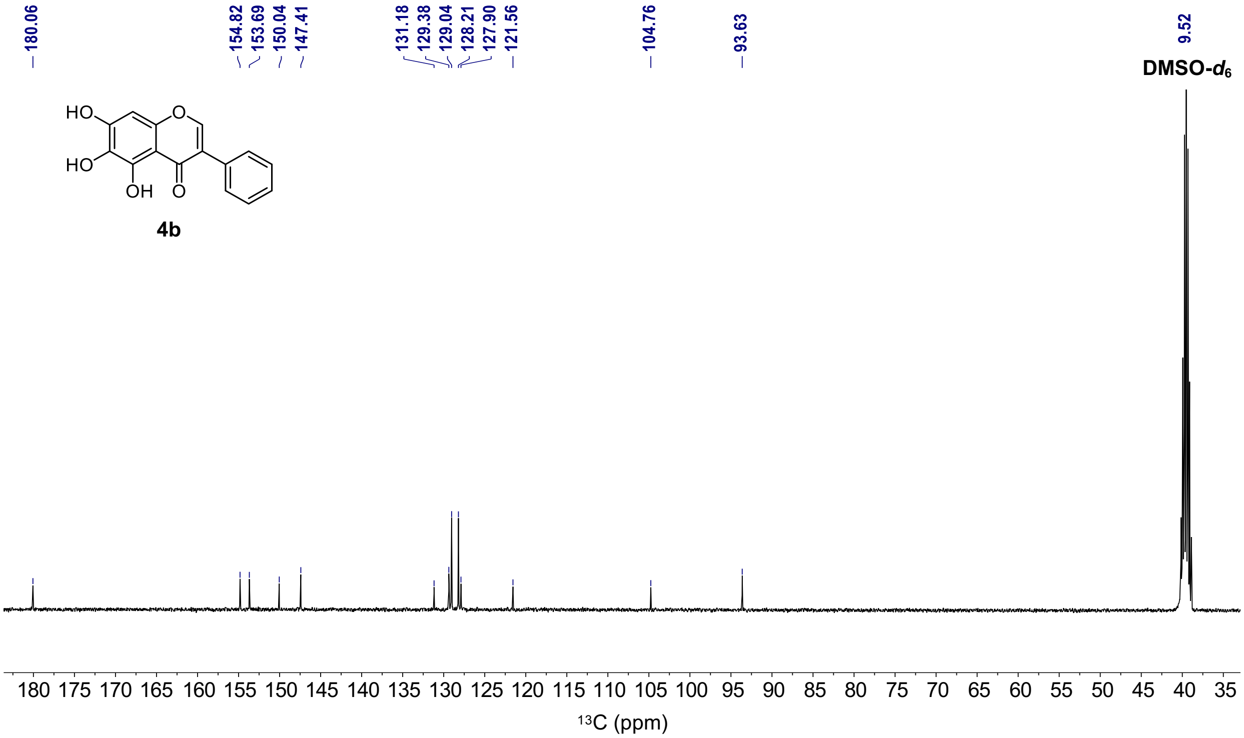

Fig. S2 ^{1}H (400 MHz) and ^{13}C (100 MHz) NMR spectra of **4b** in DMSO-d_6.

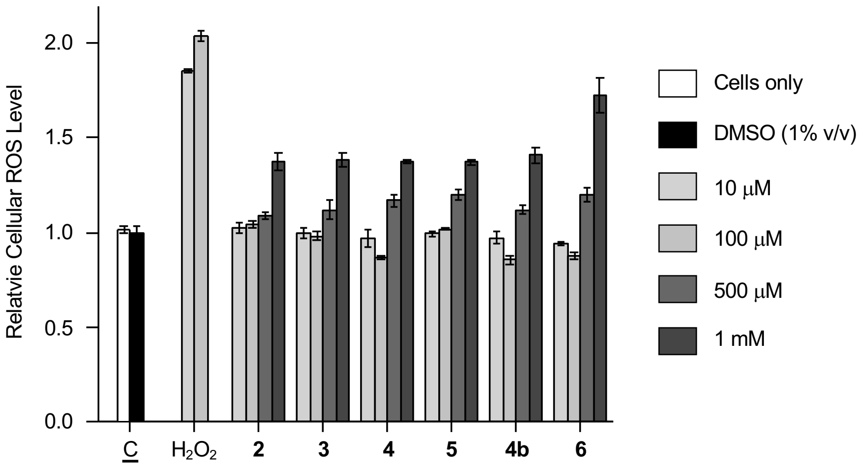

Fig. S3 Prooxidant activity of **2**–**6** and **4b** in 5Y cells. The total concentration of ROS in the cells incubated with various concentrations of the flavonoids was measured by a cellular ROS detection assay. Conditions: [flavonoid] = 0.01, 0.1, 0.5, and 1 mM (1 % v/v DMSO); [H_2O_2] = 10 and 100 μM.

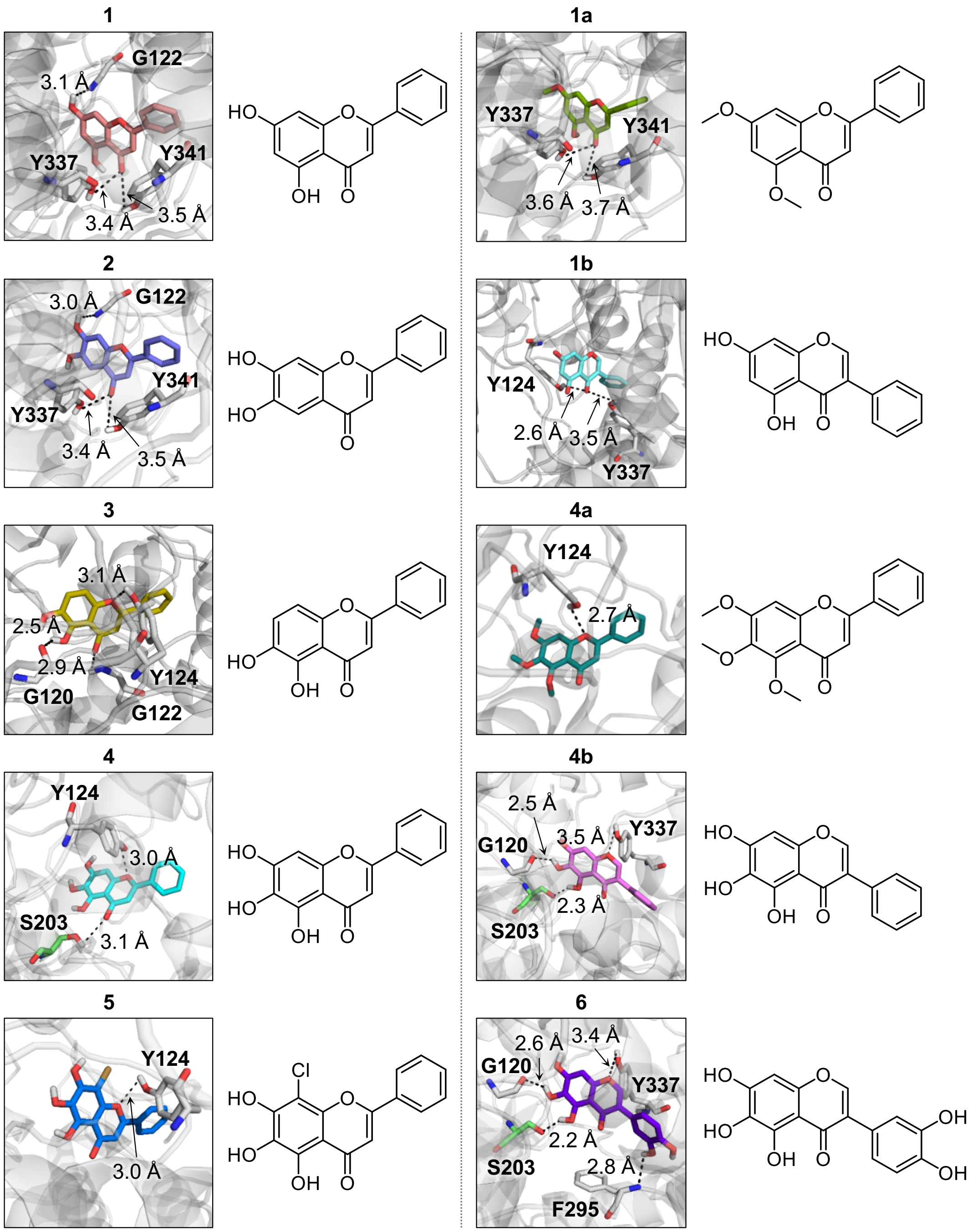

Fig. S4 Possible interactions of flavonoids against AChE. Nine docked models of **1–5**, **1a**, **1b**, **4a**, **4b**, and **6** against AChE with binding energies ranging from –10.8 to –8.5 kcal/mol were obtained. The representative docked models with the highest binding affinity towards AChE are visualized in the figure. Hydrogen bonds between flavonoids and AChE are indicated with dashed lines (2.2−3.7 Å). N, O, H, Cl (from **5**), and C (from Ser203) atoms are depicted in blue, red, white, brown, and green, respectively.

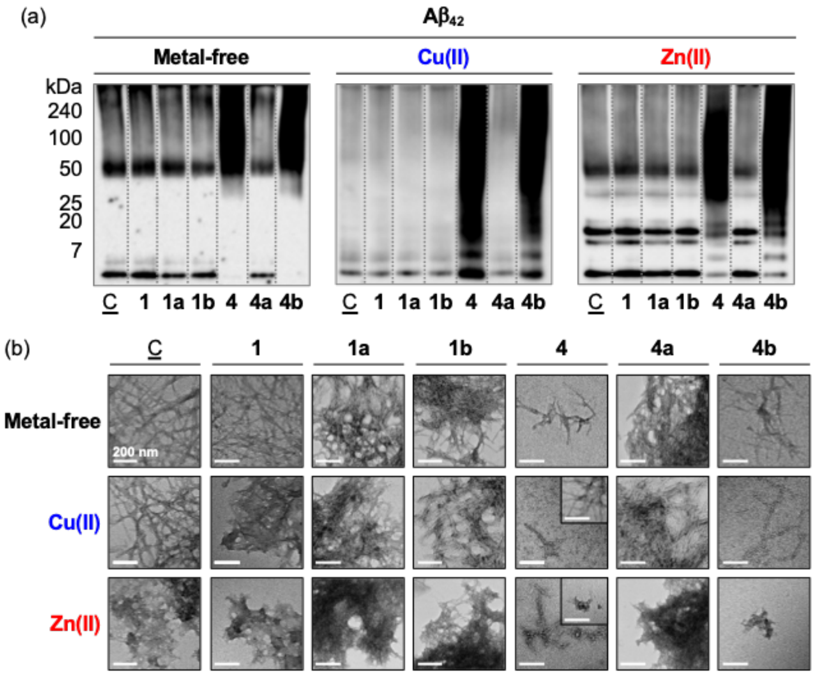

Fig. S5 Effects of **1**, **1a**, **1b**, **4**, **4a**, and **4b** on the formation of metal-free Aβ_{42} and metal–Aβ_{42} aggregates. Analyses of the (a) MW distribution and (b) morphology of the resultant Aβ_{42} species were carried out by gel/Western blot with an anti-Aβ antibody (6E10) and TEM, respectively. Lanes: (C) Aβ_{42} ± Cu(II) or Zn(II); (**1**) C + **1**; (**1a**) C + **1a**; (**1b**) C + **1b**; (**4**) C + **4**; (**4a**) C + **4a**; (**4b**) C + **4b**. Conditions: [Aβ_{42}] = 25 μM; [M(II)] = 25 μM; [flavonoid] = 50 μM (1% v/v DMSO); 20 mM HEPES, pH 7.4, 150 mM NaCl; 37 °C; 24 h; constant agitation. Scale bars = 200 nm.

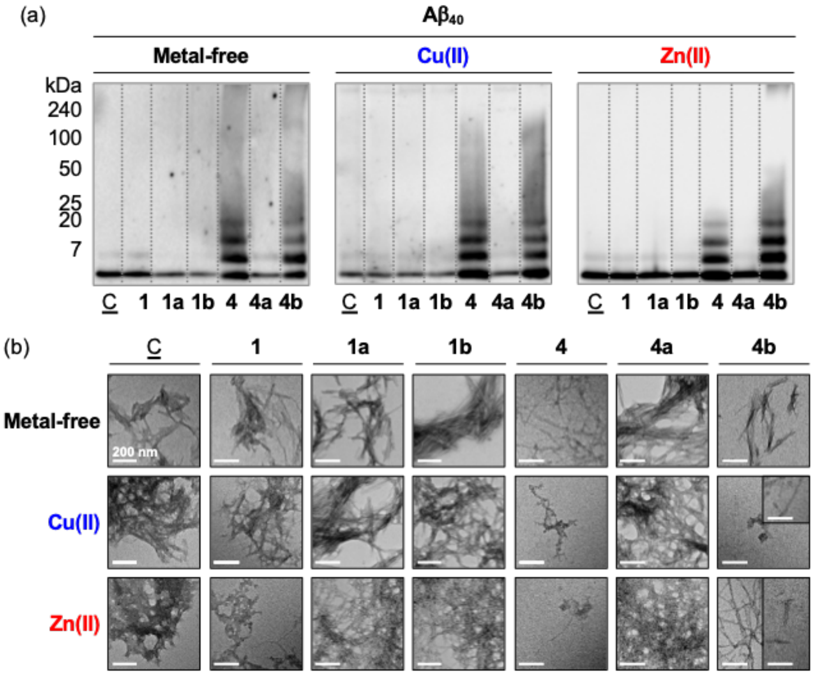

Fig. S6 Influence of **1**, **1a**, **1b**, **4**, **4a**, and **4b** on the formation of metal-free Aβ$_{40}$ and metal–Aβ$_{40}$ aggregates. Analyses of the (a) MW distribution and (b) morphology of the resultant Aβ$_{40}$ species were performed by gel/Western blot with an anti-Aβ antibody (6E10) and TEM, respectively. Lanes: (C) Aβ$_{40}$ ± Cu(II) or Zn(II); (**1**) C + **1**; (**1a**) C + **1a**; (**1b**) C + **1b**; (**4**) C + **4**; (**4a**) C + **4a**; (**4b**) C + **4b**. Conditions: [Aβ$_{40}$] = 25 μM; [M(II)] = 25 μM; [flavonoid] = 50 μM (1% v/v DMSO); 20 mM HEPES, pH 7.4, 150 mM NaCl; 37 °C; 24 h; constant agitation. Scale bars = 200 nm.

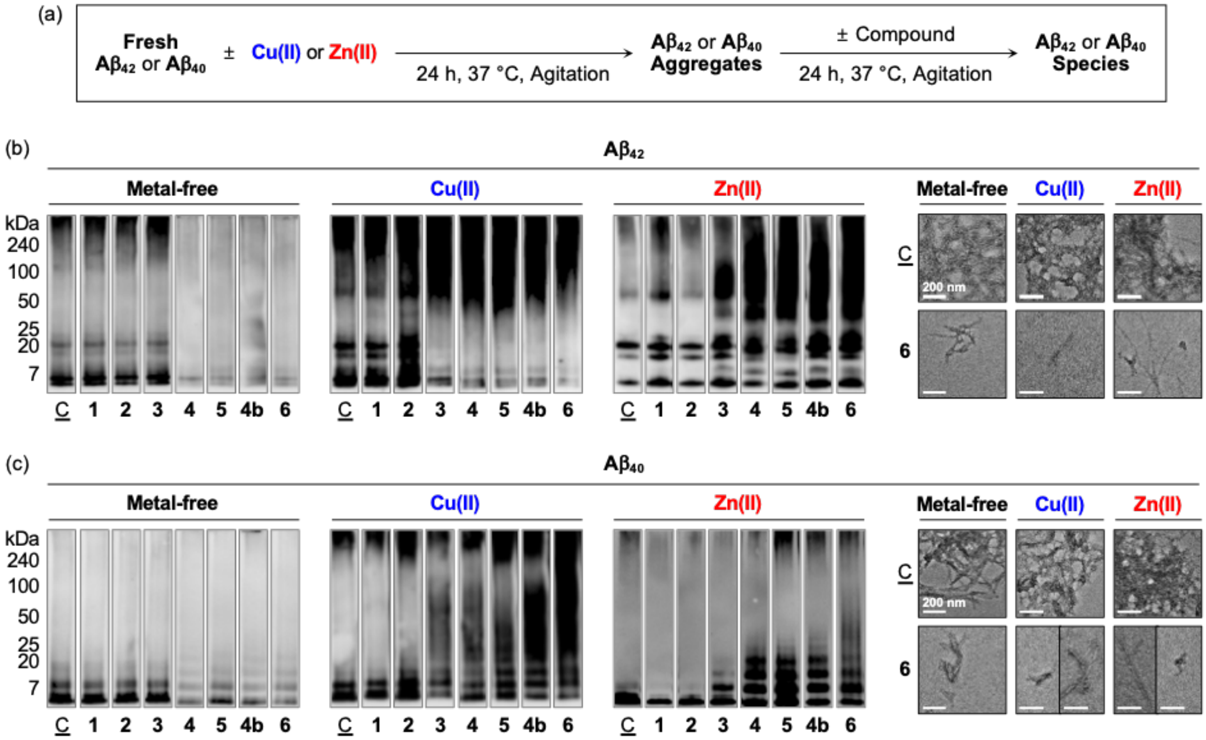

Fig. S7 Impact of **1**–**6** and **4b** on preformed metal-free Aβ and metal–Aβ aggregates. (a) Scheme of the disaggregation experiments. (b and c) Analyses of the MW distribution and morphology of the resultant (b) $Aβ_{42}$ and (c) $Aβ_{40}$ species by gel/Western blot with an anti-Aβ antibody (6E10) and TEM, respectively. Lanes: (C) Aβ ± Cu(II) or Zn(II); (**1**) C + **1**; (**2**) C + **2**; (**3**) C + **3**; (**4**) C + **4**; (**5**) C + **5**; (**4b**) C + **4b**; (**6**) C + **6**. The original gel images are shown in Fig. S9b. Conditions: [Aβ] = 25 μM; [M(II)] = 25 μM; [flavonoid] = 50 μM (1% v/v DMSO); 20 mM HEPES, pH 7.4, 150 mM NaCl; 37 °C; 24 h; constant agitation. Scale bars = 200 nm.

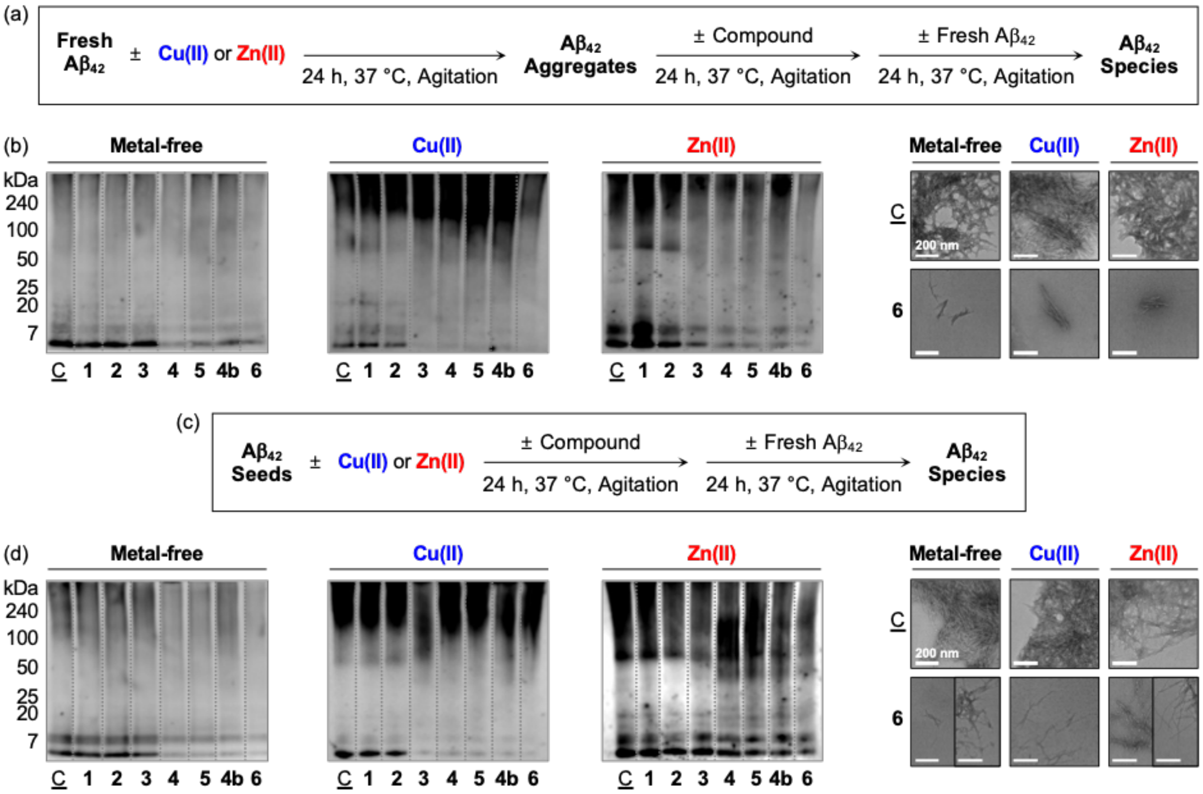

Fig. S8 Effects of **1–6** and **4b** on preformed metal-free and metal-added Aβ_{42} aggregates and seeds in the presence of freshly prepared Aβ_{42}. (a and c) Scheme of the aggregation experiments. (b and d) Analyses of the MW distribution and morphology of the resultant Aβ_{42} species by gel/Western blot with an anti-Aβ antibody (6E10) and TEM, respectively. Lanes: (C) Aβ ± Cu(II) or Zn(II); (**1**) C + **1**; (**2**) C + **2**; (**3**) C + **3**; (**4**) C + **4**; (**5**) C + **5**; (**4b**) C + **4b**; (**6**) C + **6**. Conditions: [Aβ_{42}] = 25 μM; Aβ_{42} seeds (10% v/v); [M(II)] = 25 μM; [flavonoid] = 50 μM (1% v/v DMSO); 20 mM HEPES, pH 7.4, 150 mM NaCl; 37 °C; 24 h; constant agitation. Scale bars = 200 nm.

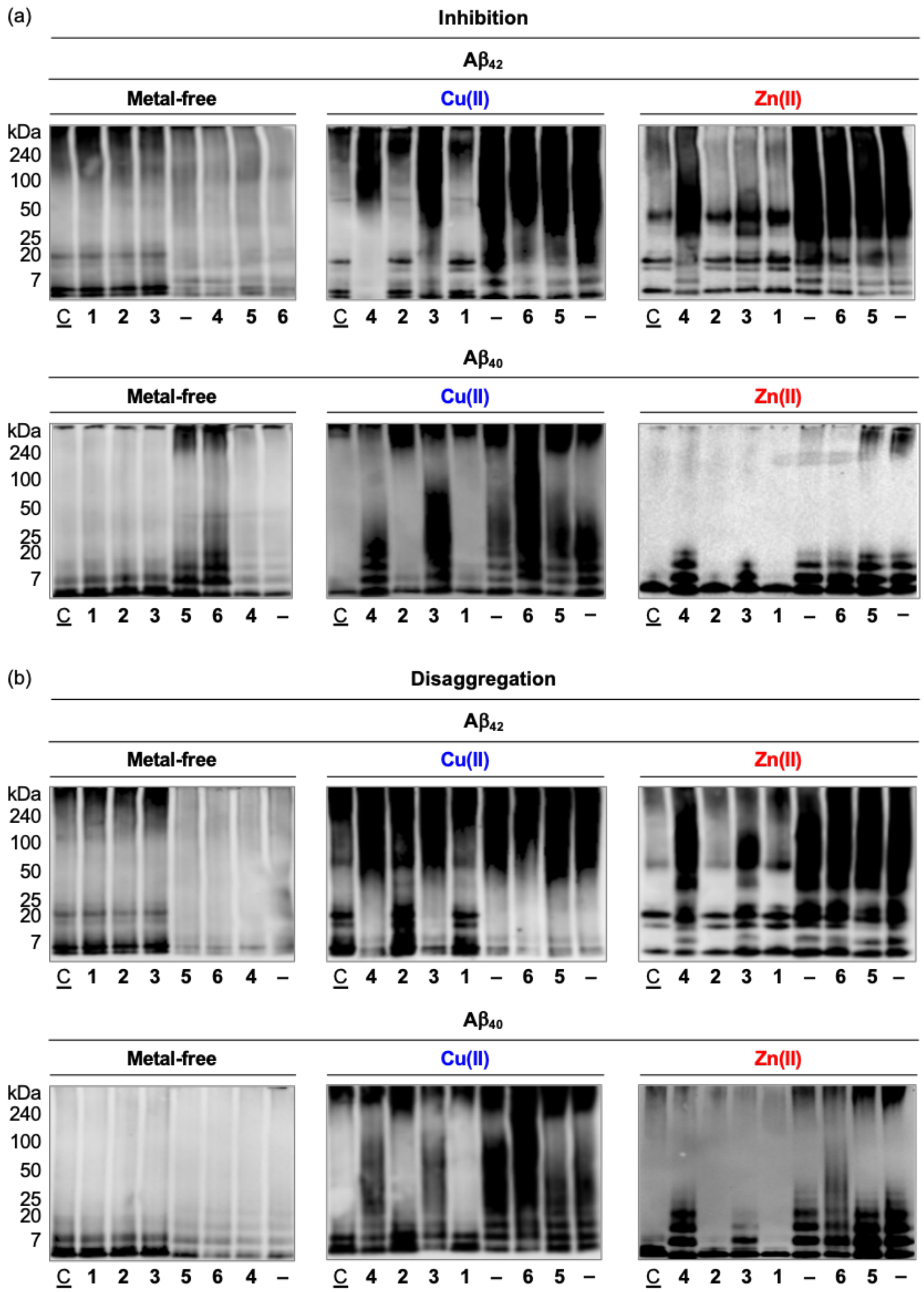

Fig. S9 Original gel images of Figs. 4 and S7.

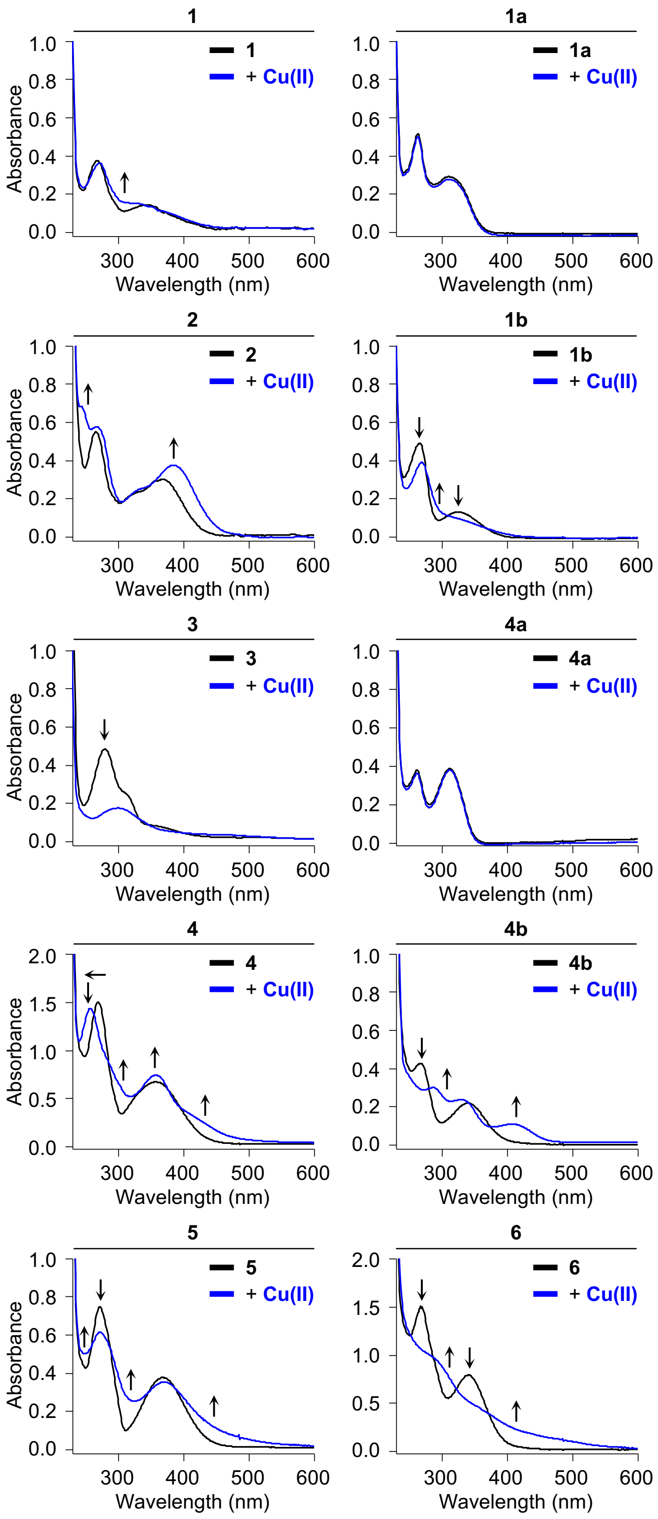

Fig. S10 Interaction of the flavonoids with Cu(II) monitored by Abs spectroscopy. Conditions: [flavonoid] = 50 μM; [Cu(II)] = 50 μM; 20 mM HEPES, pH 7.4, 150 mM NaCl; room temperature.

(a)

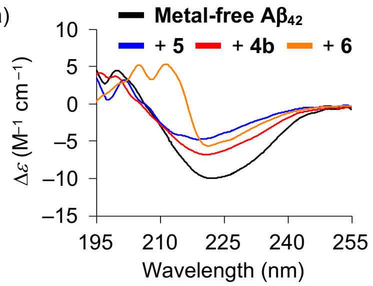

(b)

	**Metal-free Aβ_{42}**	**+ 5**	**+ 4b**	**+ 6**
α-Helix (%)	19.2 (± 2.1)	1.2 (± 1.2)	0.4 (± 0.4)	1.5 (± 1.5)
β-Sheet (%)	73.7 (± 0.7)	66.7 (± 15.3)	35.5 (± 5.7)	46.7 (± 2.6)
Turn (%)	5.7 (± 1.0)	12.5 (± 0.7)	10.3 (± 1.8)	38.1 (± 7.6)
Ramdom Coils (%)	1.5 (± 1.5)	19.7 (± 15.5)	53.8 (± 5.9)	13.6 (± 5.6)

Fig. S11 Change in the secondary structure of metal-free Aβ_{42} upon incubation with **5**, **4b**, or **6**. (a) CD spectra of the samples containing metal-free Aβ_{42} in the absence and presence of **5**, **4b**, or **6**. (b) Structural composition of the samples from each CD spectrum analyzed by BeStSel.[25] Conditions: [Aβ_{42}] = 100 μM; [flavonoid] = 200 μM (1% v/v DMSO); 20 mM HEPES, pH 7.4, 150 mM NaF; 37 °C; 24 h; constant agitation.

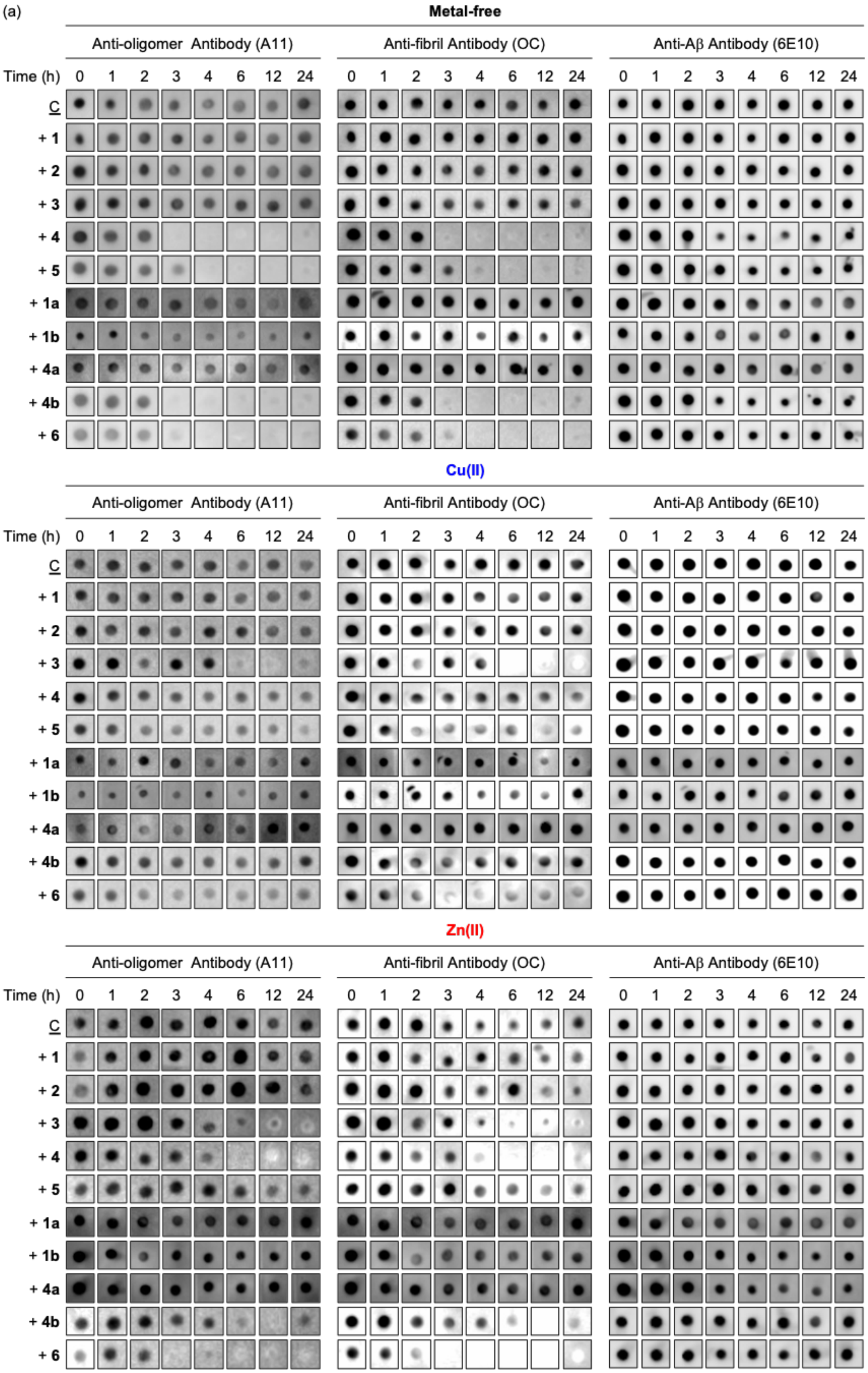
(a)
Metal-free
Cu(II)
Zn(II)
Anti-oligomer Antibody (A11)
Anti-fibril Antibody (OC)
Anti-Aβ Antibody (6E10)
Time (h)
0 1 2 3 4 6 12 24
C
+ 1
+ 2
+ 3
+ 4
+ 5
+ 1a
+ 1b
+ 4a
+ 4b
+ 6

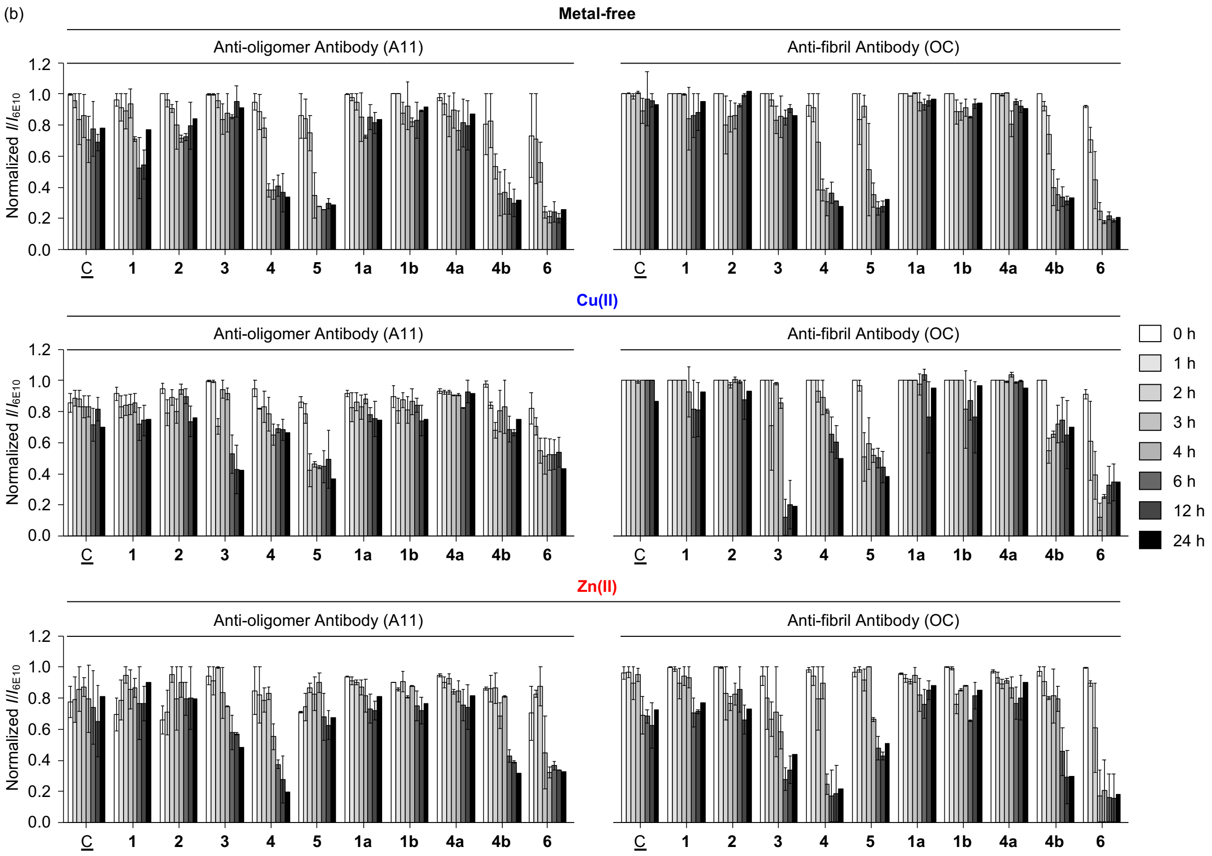

Fig. S12 Formation of metal-free Aβ_{42} and metal–Aβ_{42} oligomers and fibrils upon treatment of flavonoids monitored by the dot blot assay. (a) Dot blots of metal-free Aβ_{42} and metal–Aβ_{42} species produced by incubation with flavonoids detected by anti-oligomer (A11), anti-fibril (OC), and anti-Aβ (6E10) antibodies. Note that the anti-fibril antibody, OC, may monitor both monomeric and aggregated forms of Aβ.[26] (b) Quantification of A11- and OC-detectable Aβ species. The value of I / I_{6E10} indicates the intensity of A11- or OC-detectable Aβ species quantified, relative to that detected from 6E10. Conditions: [Aβ_{42}] = 100 μM; [M(II)] = 100 μM; [flavonoid] = 200 μM (1% v/v DMSO); 20 mM HEPES, pH 7.4, 150 mM NaCl; 37 °C; 0, 1, 2, 3, 4, 6. 12, and 24 h; constant agitation.

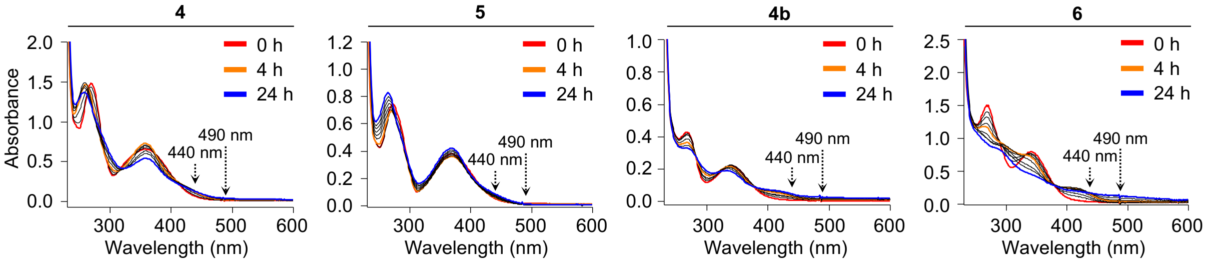

Fig. S13 Absorbance spectra of **4–6** and **4b**. The compounds' Abs was shown to interfere with the excitation and emission wavelengths of ThT ($\lambda_{em}/\lambda_{ex}$ = 440/490 nm). Conditions: [flavonoid] = 50 μM (1% v/v DMSO); 20 mM HEPES, pH 7.4, 150 mM NaCl.

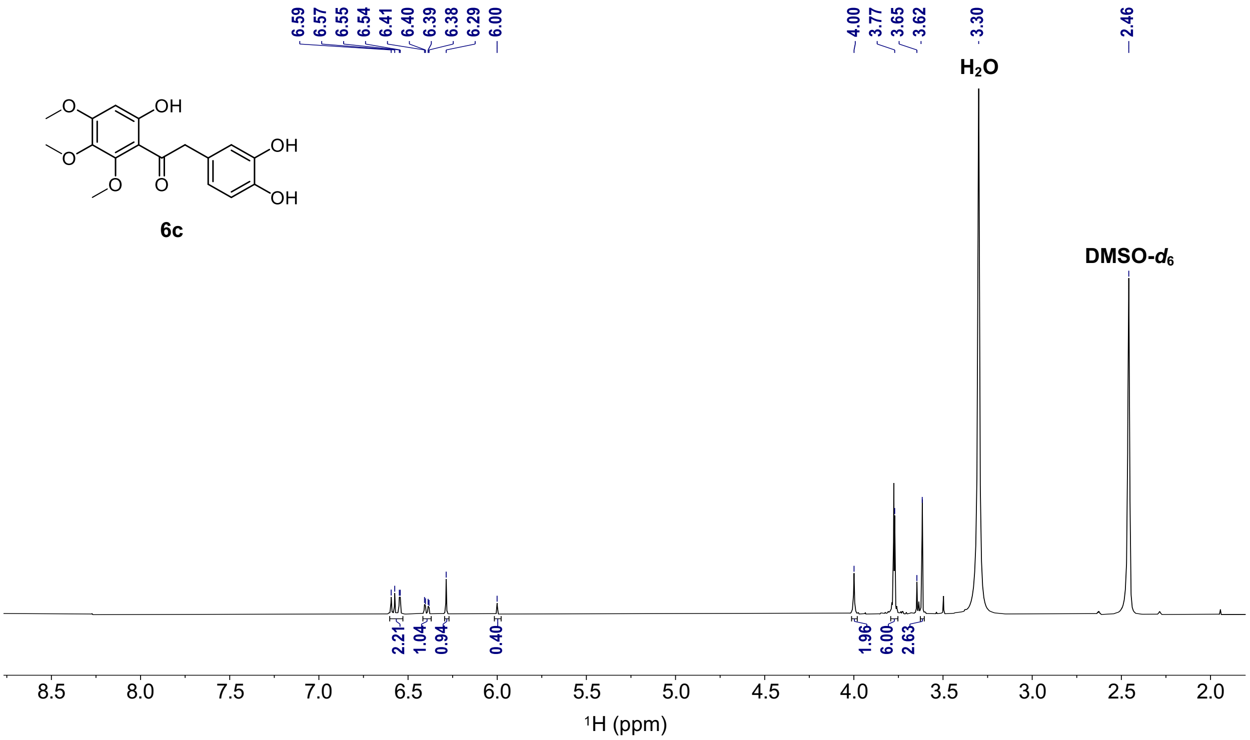

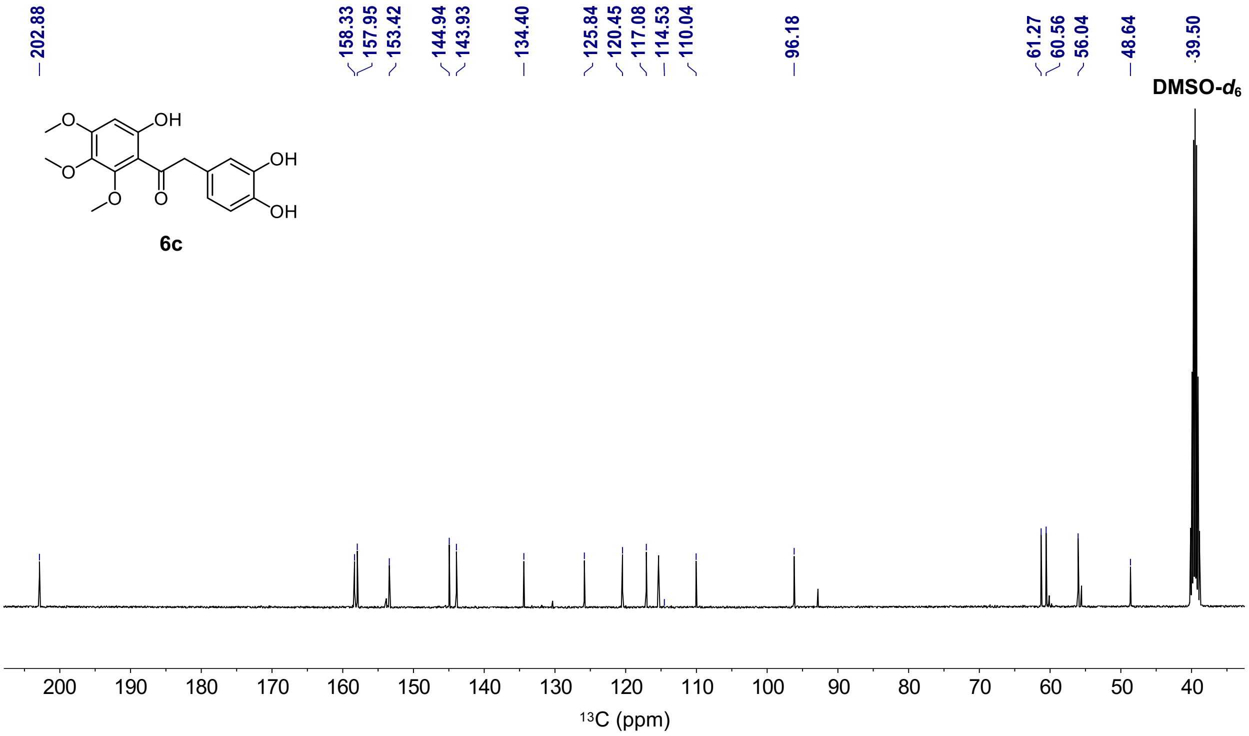

Fig. S14 ^{1}H (400 MHz) and ^{13}C (100 MHz) NMR spectra of **6c** in DMSO-d_6.

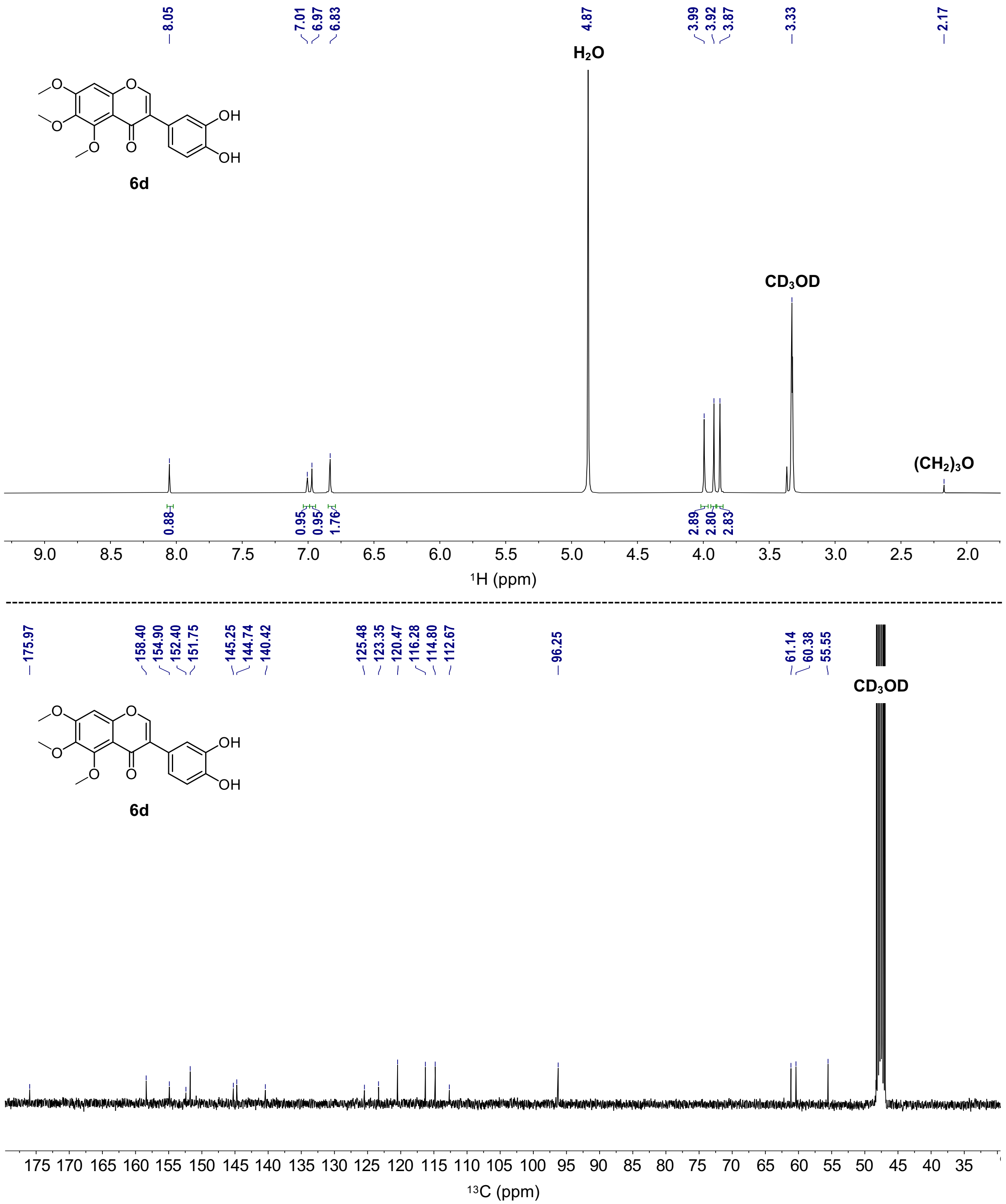

Fig. S15 ^{1}H (400 MHz) and ^{13}C (100 MHz) NMR spectra of **6d** in CD_3OD.

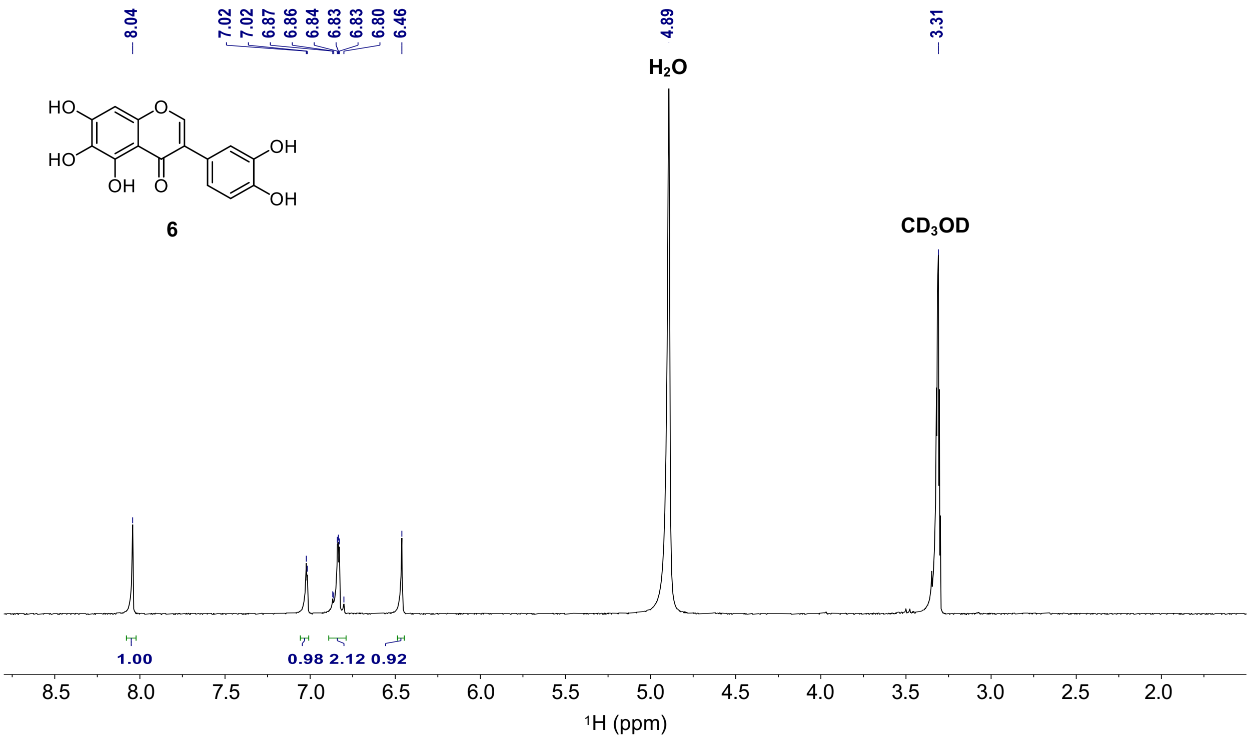

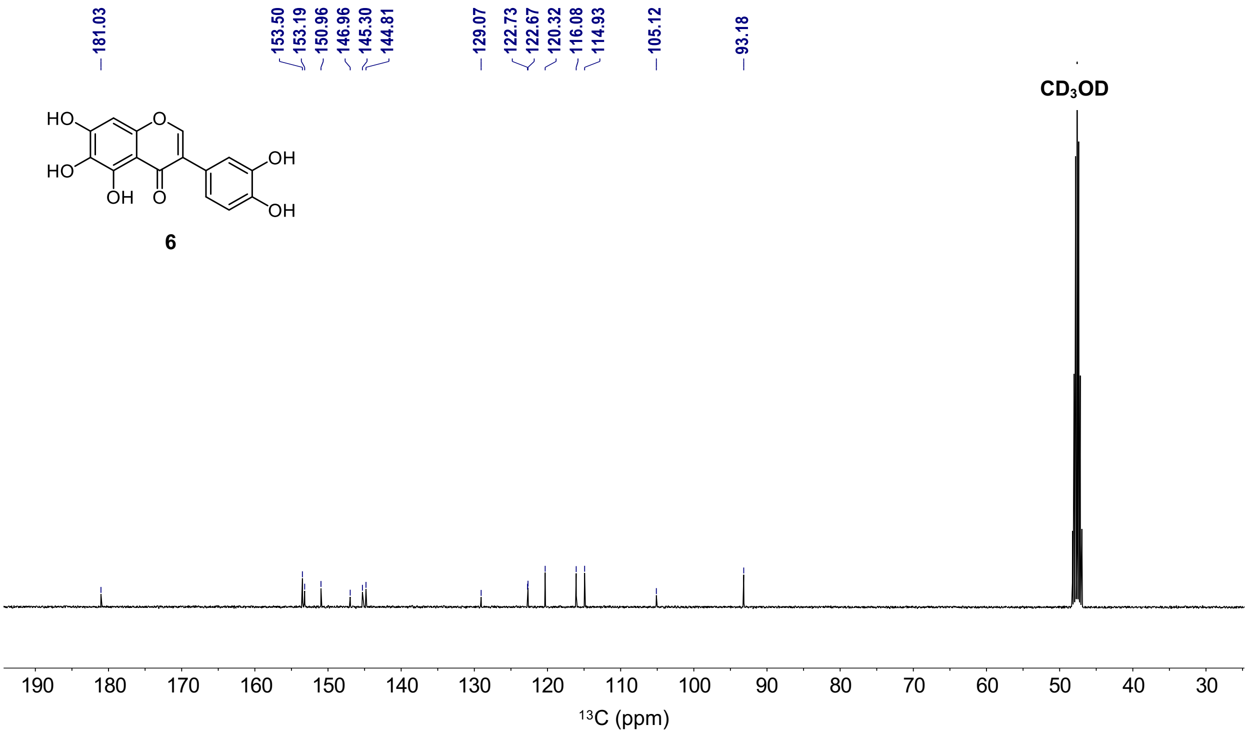

Fig. S16 ^{1}H (400 MHz) and ^{13}C (100 MHz) NMR spectra of **6** in CD_3OD.

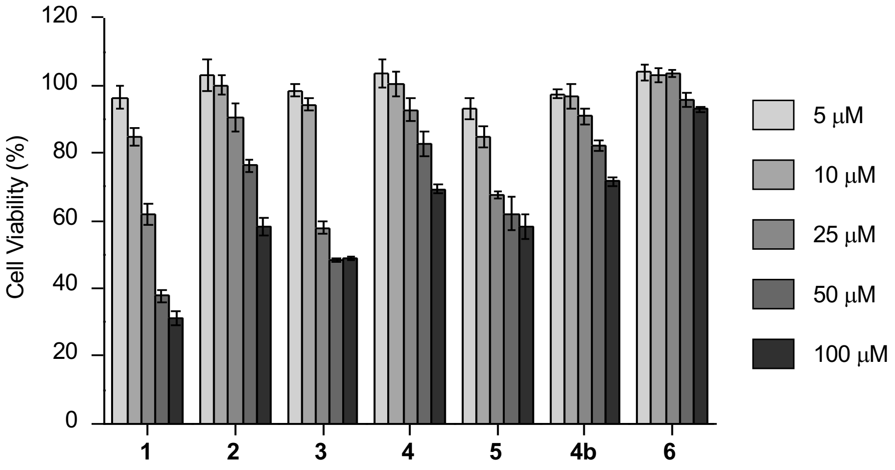

Fig. S17 Cytotoxicity of **1–6** and **4b** in 5Y cells. Viability (%) of cells incubated with various concentrations of **1–6** and **4b** was determined by the MTT assay. Cell survival was calculated in comparison to that of the cells treated with an equivalent amount of DMSO (1 % v/v). Error bars indicate the standard error from three independent experiments. Conditions: [flavonoid] = 5, 10, 25, 50, and 100 μM (1 % v/v DMSO); 37 °C; 24 h.

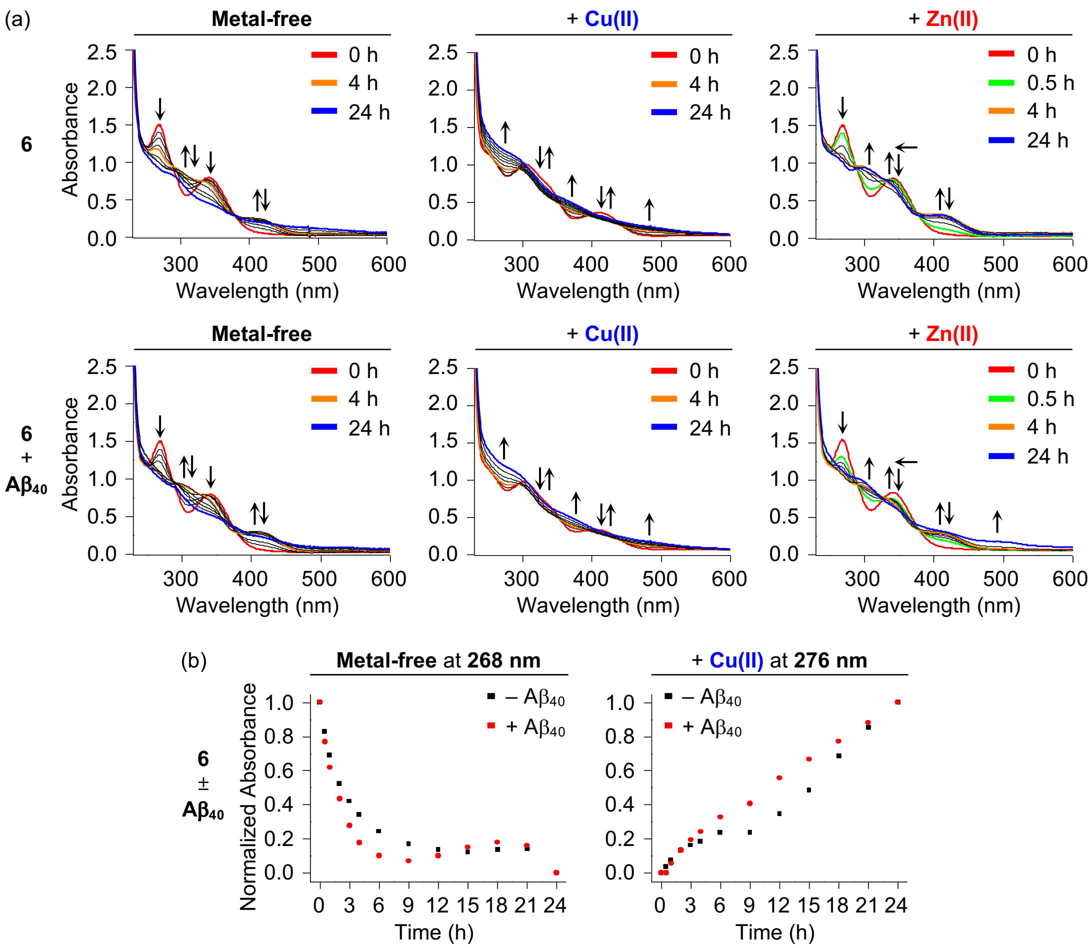

Fig. S18 Chemical transformations of **6**. (a) Optical changes of **6** in the absence and presence of Aβ_{40} with or without metal ions, monitored by Abs spectroscopy. Conditions: [Aβ_{40}] = 25 μM; [M(II)] = 25 μM; [**6**] = 50 μM; 20 mM HEPES, pH 7.4, 150 mM NaCl; 37 °C; 0–24 h; no agitation. (b) Change in the normalized absorbance of **6** with or without Aβ_{40} at 268 nm and 276 nm in the absence and presence of Cu(II), respectively, for 24 h.

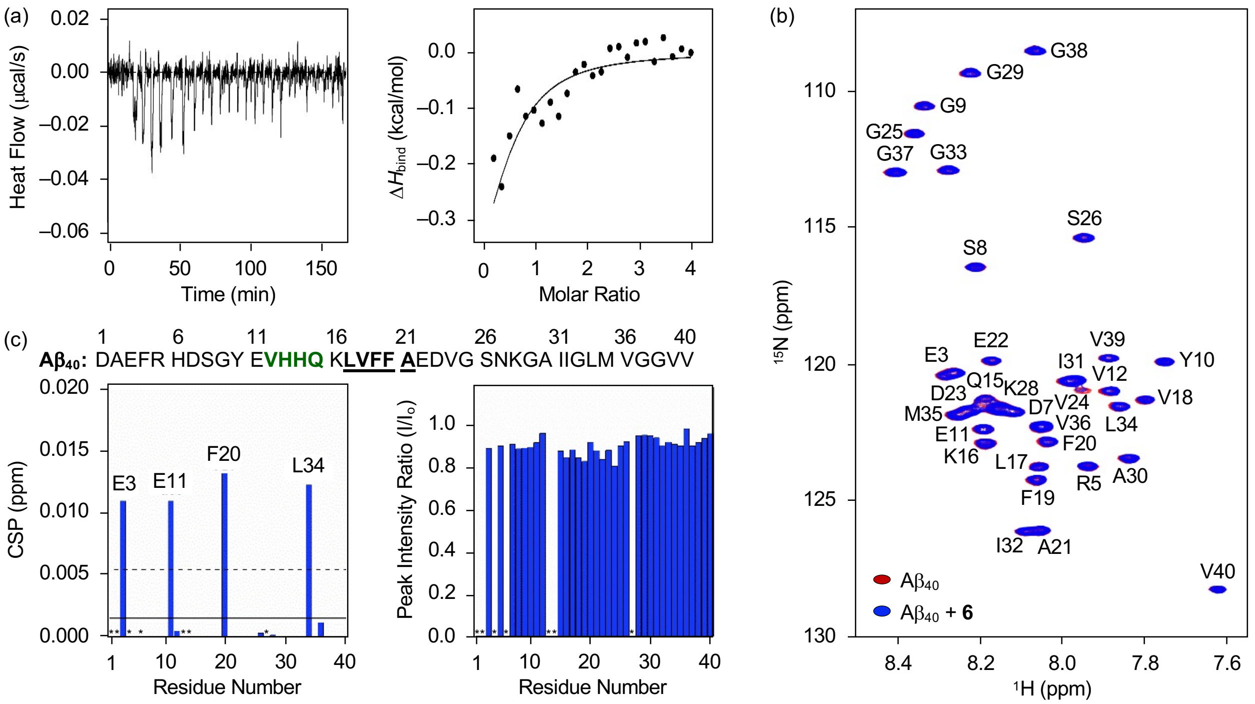

Fig. S19 Interaction of **6** with monomeric Aβ$_{40}$. (a) Binding of **6** with Aβ$_{40}$ recorded by ITC. The ITC thermogram (left) and binding isotherm (right) of **6** with Aβ$_{40}$ monomer are shown. The solid line indicates the best fit of the ITC data to a one-set-of-sites binding model. Conditions: [Aβ$_{40}$] = 40 μM; [**6**] = 800 μM (1% v/v DMSO); 20 mM HEPES, pH 7.4; 10 °C. (b) 2D ^{1}H–^{15}N SOFAST-HMQC NMR (800 MHz) spectra of ^{15}N-labeled Aβ$_{40}$ with and without **6**. (c) CSPs and peak intensity ratios of amino acid residues in ^{15}N-labeled Aβ$_{40}$ upon addition of **6**. Amino acid residues involved in the β-turn motif and the self-recognition site are highlighted in bold/green and bold/underline, respectively. The unresolved peaks (*i.e.*, Asp1, Ala2, Phe4, His6, His13, His14, and Asn27) are indicated with asterisks. Two horizontal lines indicate the average chemical shift (solid line) plus one standard deviation (dashed line). Conditions: [^{15}N-labeled Aβ$_{40}$] = 33 μM; [**6**] = 100 μM (1% v/v DMSO); 20 mM HEPES, pH 7.4; 10% v/v D_2O; 10 °C.

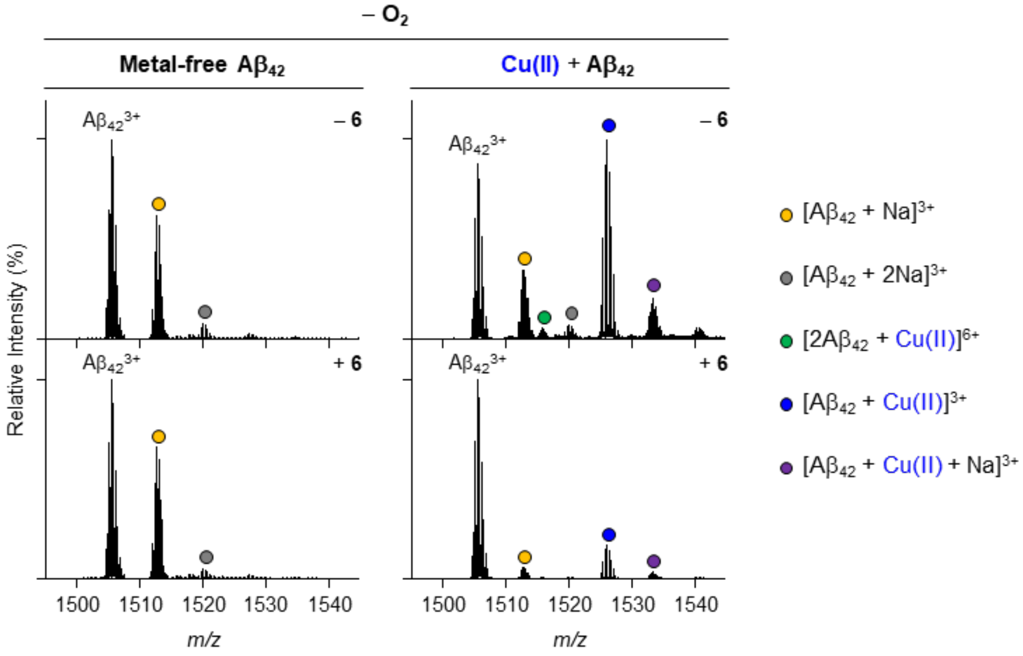

Fig. S20 Interactions of **6** with metal-free and Cu(II)-added Aβ_{42} in the absence of O_2. Conditions: [Aβ_{42}] = 100 μM; [Cu(II)] = 100 μM; [**6**] = 200 μM (1% v/v DMSO); 20 mM ammonium acetate, pH 7.4; 37 °C; 3 h; no agitation. The samples were prepared in a N_2-filled glove box and diluted by 10-fold with ddH_2O before injection into the mass spectrometer.

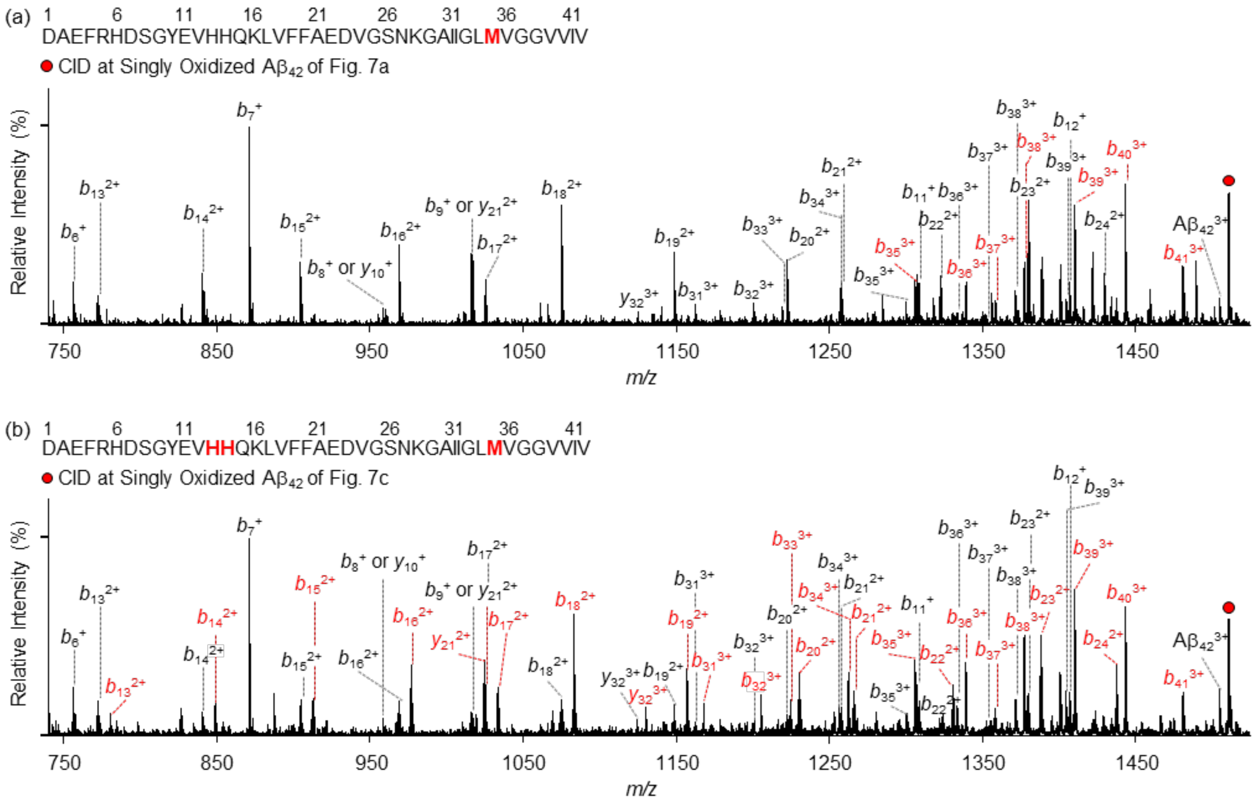

Fig. S21 Detailed analysis of ESI–MS^2 spectra shown in Fig. 7.

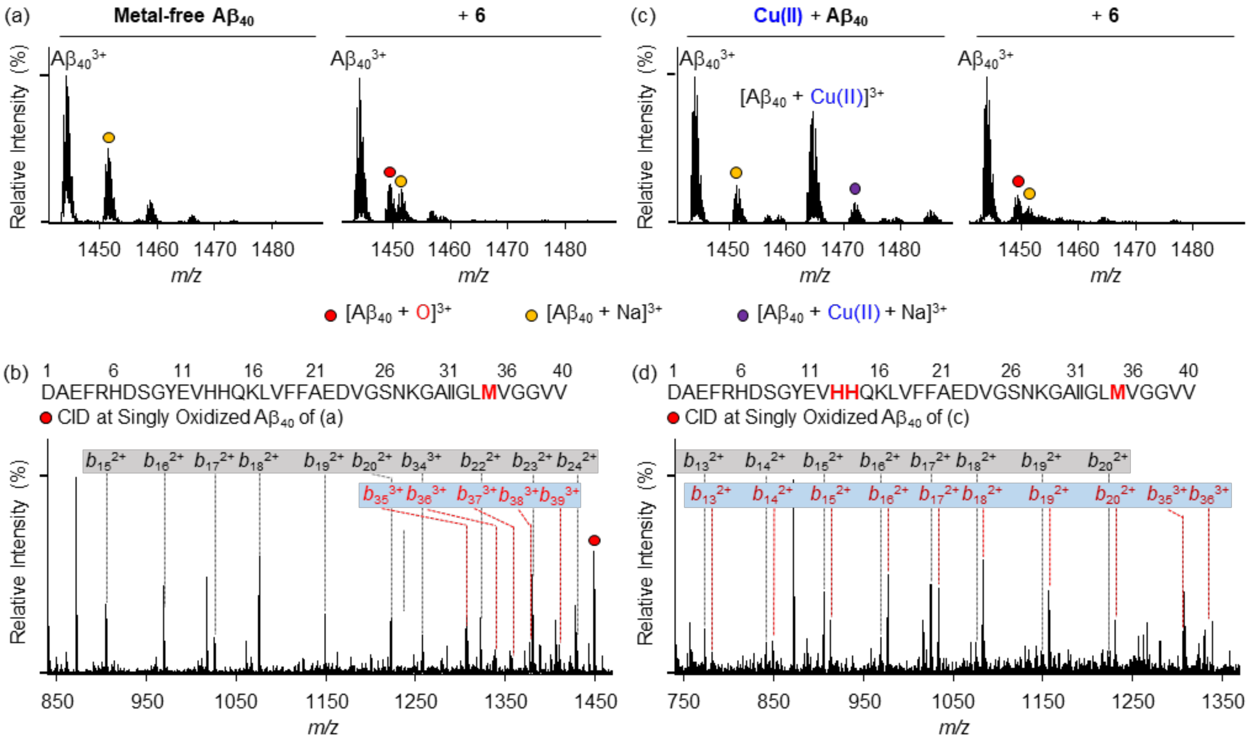

Fig. S22 Interactions of **6** with metal-free and Cu(II)-added Aβ_{40}. ESI–MS spectra of +3-charged Aβ_{40} and ESI–MS^2 analyses of the singly oxidized Aβ_{40} (1,449 *m*/*z*) were obtained upon treated of **6** in the (a and b) absence and (c and d) presence of Cu(II). The peaks highlighted as a red circle correspond to the singly oxidized Aβ_{40}. Detailed analysis of tandem spectra is shown in Fig. S23. Conditions: [Aβ_{40}] = 100 μM; [Cu(II)] = 100 μM; [**6**] = 200 μM (1% v/v DMSO); 20 mM ammonium acetate, pH 7.4; 37 °C; 3 h; constant agitation. The samples were diluted by 10-fold with ddH_2O before injection into the mass spectrometer.

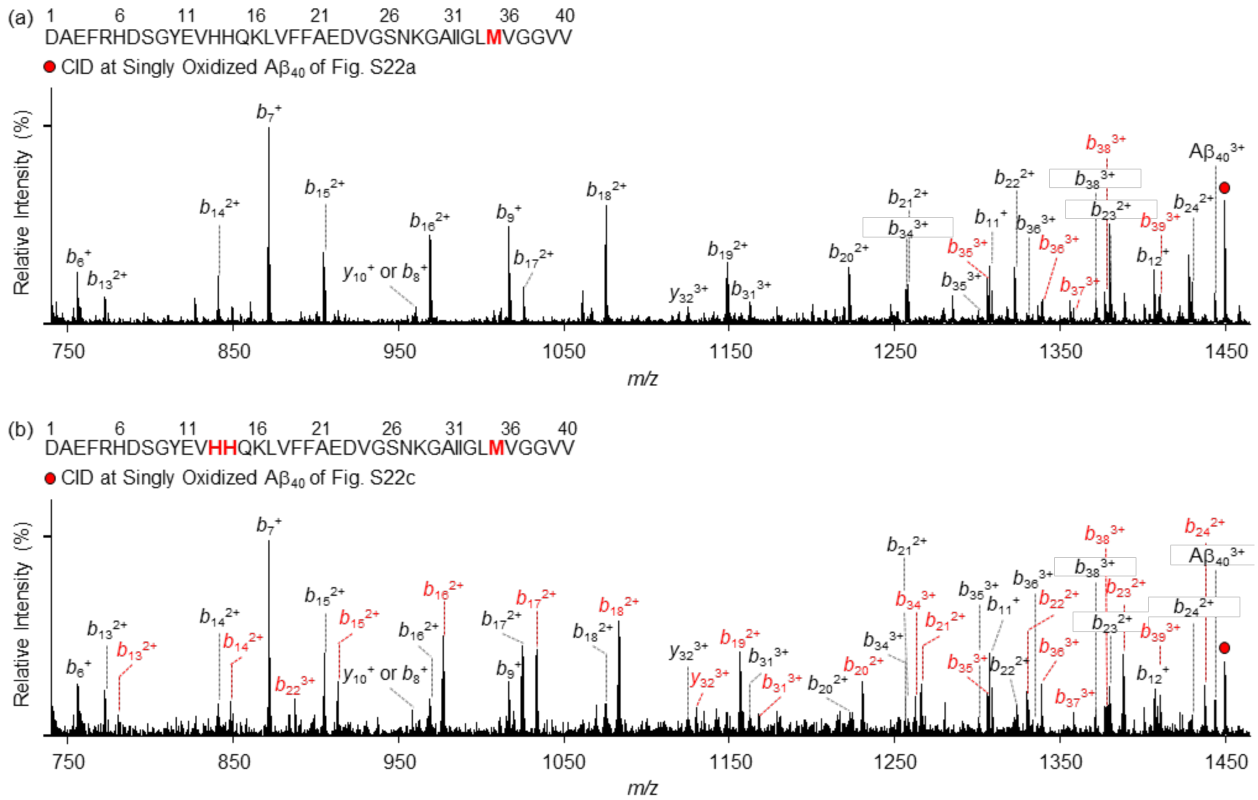

Fig. S23 Detailed analysis of ESI–MS^2 spectra presented in Fig. S22.

References

1. E. Marzec, M. Świtalska, M. Winiewska-Szajewska, J. Wójcik, J. Wietrzyk, A. M. Maciejewska, J. Poznański and A. Mieczkowski, *IUBMB Life*, 2020, **72**, 1250.
2. H. Gao and J. Kawabata, *Bioorg. Med. Chem.*, 2005, **13**, 1661.
3. R. G. Parr and Y. Weitao, *Density-Functional Theory of Atoms and Molecules*, Oxford University Press, New York, 1995.
4. A. D. Bochevarov, E. Harder, T. F. Hughes, J. R. Greenwood, D. A. Braden, D. M. Philipp, D. Rinaldo, M. D. Halls, J. Zhang and R. A. Friesner, *Int. J. Quantum Chem.,* 2013, **113**, 2110.
5. C. Lee, W. Yang and R. G. Parr, *Phys. Rev. B,* 1988, **37**, 785.
6. S. Grimme, J. Antony, S. Ehrlich and H. Krieg, *J. Chem. Phys.,* 2010, **132**, 154104.
7. A. D. Becke, *Phys. Rev. A,* 1988, **38**, 3098.
8. P. J. Hay and W. R. Wadt, *J. Chem. Phys.,* 1985, **82**, 299.
9. P. J. Hay and W. R. Wadt, *J. Chem. Phys.,* 1985, **82**, 270.
10. W. R. Wadt and P. J. Hay, *J. Chem. Phys.,* 1985, **82**, 284.
11. T. H. Dunning Jr., *J. Chem. Phys.,* 1989, **90**, 1007.
12. S. R. Edinger, C. Cortis, P. S. Shenkin and R. A. Friesner, *J. Phys. Chem. B*, 1997, **101**, 1190.
13. M. Friedrichs, R. Zhou, S. R. Edinger and R. A. Friesner, *J. Phys. Chem. B*, 1999, **103**, 3057.
14. B. Marten, K. Kim, C. Cortis, R. A. Friesner, R. B. Murphy, M. N. Ringnalda, D. Sitkoff and B. Honig, *J. Phys. Chem.*, 1996, **100**, 11775.
15. L. R. C. Barclay, S. J. Locke, J. M. MacNeil, J. VanKessel, G. W. Burton and K. U. Ingold, *J. Am. Chem. Soc.,* 1984, **106**, 2479.
16. H. J. Lee, K. J. Korshavn, Y. Nam, J. Kang, T. J. Paul, R. A. Kerr, I. S. Youn, M. Ozbil, K. S. Kim, B. T. Ruotolo, R. Prabhakar, A. Ramamoorthy and M. H. Lim, *Chem. Eur. J.,* 2017, **23**, 2706.
17. Y. Ji, H. J. Lee, M. Kim, G. Nam, S. J. C. Lee, J. Cho, C.-M. Park and M. H. Lim, *Inorg. Chem.,* 2017, **56**, 6695.
18. O. Trott and A. J. Olson, *J. Comput. Chem.,* 2010, **31**, 455.
19. Y. Bourne, J. Grassi, P. E. Bougis and P. Marchot, *J. Biol. Chem.*, 1999, **274**, 30370.
20. G. M. Morris, R. Huey, W. Lindstrom, M. F. Sanner, R. K. Belew, D. S. Goodsell and A. J. Olson, *J. Comput. Chem.,* 2009, **30**, 2785.
21. M. W. Beck, J. S. Derrick, J. M. Suh, M. Kim, K. J. Korshavn, R. A. Kerr, W. J. Cho, S. D. Larsen, B. T. Ruotolo, A. Ramamoorthy and M. H. Lim, *ChemMedChem*, 2017, **12**, 1828.

22. Y. Yi, J. Han, M. H. Park, N. Park, E. Nam, H. K. Jin, J.-s. Bae and M. H. Lim, *Chem. Commun.,* 2019, **55**, 5847.
23. D. D. Mruk and C. Y. Cheng, *Spermatogenesis*, 2011, **1**, 121.
24. E. H. Nielsen, M. Nybo and S. E. Svehag, *Methods Enzymol.*, 1999, **309**, 491.
25. A. Micsonai, F. Wien, L. Kernya, Y.-H. Lee, Y. Goto, M. Réfrégiers and J. Kardos, *Proc. Natl. Acad. Sci. U. S. A.*, 2015, **112**, E3095.
26. A. Hatami, R. Albay, 3rd, S. Monjazeb, S. Milton and C. Glabe, *J. Biol. Chem.*, 2014, **289**, 32131.
27. T. Ikenoue, Y.-H. Lee, J. Kardos, M. Saiki, H. Yagi, Y. Kawata and Y. Goto, *Angew. Chem. Int. Ed.*, 2014, **53**, 7799.
28. D. S. Doering and P. Matsudaira, *Biochemistry*, 1996, **35**, 12677.
29. F. Delaglio, S. Grzesiek, G. W. Vuister, G. Zhu, J. Pfeifer and A. Bax, *J. Biomol. NMR*, 1995, **6**, 277.
30. Sparky 3, https://www.cgl.ucsf.edu/home/sparky/, (accessed August 2020).
31. K. J. Korshavn, M. Jang, Y. J. Kwak, A. Kochi, S. Vertuani, A. Bhunia, S. Manfredini, A. Ramamoorthy and M. H. Lim, *Sci. Rep.*, 2015, **5**, 17842.
32. F. A. Mulder, D. Schipper, R. Bott and R. Boelens, *J. Mol. Biol.*, 1999, **292**, 111.

Appendix A. Cartesian coordinates of the optimized geometries.

==============================

1

==============================

C	-2.195320368	0.547892809	-4.678889751
C	-1.945337534	1.905872226	-4.480629921
C	-1.693511486	2.384855509	-3.188538551
C	-1.683222175	1.535597444	-2.076026917
C	-1.934610486	0.188077137	-2.293468952
C	-2.193931818	-0.341626465	-3.568579197
C	-2.454141140	-1.760766387	-3.736310005
C	-2.433100939	-2.538046360	-2.512343645
C	-2.160746098	-1.964832544	-1.309964061
O	-1.915832758	-0.629345596	-1.191856980
H	-1.950446844	2.571601152	-5.338489532
H	-1.483989477	1.920775890	-1.084413290
O	-2.688469887	-2.269680738	-4.856754780
O	-2.436536312	0.087731399	-5.910073757
H	-2.586309671	-0.894972444	-5.810859680
C	-2.108186722	-2.653860092	-0.006875373
C	-2.012812138	-4.053714275	0.066558458
C	-2.153409481	-1.915189743	1.187680483
H	-1.948274374	-4.643022060	-0.841504633
H	-2.221145630	-0.834829688	1.143552184
C	-1.979984641	-4.696137905	1.300252199
C	-2.118864536	-2.562927246	2.420063496
H	-2.159806252	-1.978021145	3.334208012
C	-2.034399033	-3.954107285	2.482083797
H	-1.903575182	-5.778603554	1.339214325
H	-2.007266760	-4.457617760	3.443946838
H	-2.653301716	-3.594697475	-2.585030317
O	-1.445618868	3.699834824	-2.950443983
H	-1.476941943	4.186740875	-3.784616232

==============================

$1^{\cdot+}$

==============================

C	-2.088867903	0.537009895	-4.688344002
C	-2.059590578	1.936011553	-4.506514549
C	-2.029988050	2.440476894	-3.214294195
C	-2.029489517	1.572589517	-2.072799206
C	-2.058186769	0.189122260	-2.275722980
C	-2.086154938	-0.354452670	-3.550178289
C	-2.120449781	-1.790661097	-3.751064062
C	-2.120751381	-2.562110662	-2.535015583
C	-2.089860201	-1.963194966	-1.288719773
O	-2.055066586	-0.605011225	-1.175049067
H	-2.061082840	2.579167366	-5.379514217
H	-2.007418394	1.997543693	-1.076066256
O	-2.150436401	-2.281162977	-4.903470993
O	-2.118607044	0.014660744	-5.879664898
H	-2.137730837	-1.019381762	-5.755851746
C	-2.093415260	-2.648307323	-0.004822709
C	-2.011418343	-4.059198856	0.061115388
C	-2.185765982	-1.909674525	1.196259141
H	-1.921608567	-4.654372215	-0.839826584
H	-2.253813982	-0.829253078	1.163496971
C	-2.022579193	-4.704178810	1.289167643
C	-2.199643373	-2.564331532	2.419569016
H	-2.274940252	-1.989901423	3.336898565
C	-2.118554354	-3.961450815	2.471279383
H	-1.954430223	-5.786128044	1.328998923
H	-2.128445387	-4.469493389	3.430545330
H	-2.163991213	-3.638566256	-2.628045321
O	-2.000975609	3.739420414	-2.908965826
H	-2.002430916	4.299263000	-3.700512171

==============================

2

==============================

C	0.622462511	0.134940445	-2.619236469
C	1.590379953	-0.597965598	-1.965734482
C	1.254297018	-1.811640859	-1.314370871
C	-0.056055281	-2.270164013	-1.330247283
C	-1.023457885	-1.513682604	-1.998075366
C	-0.708614886	-0.314842075	-2.645905018
C	-1.760906219	0.450387746	-3.342654228
C	-3.078402519	-0.170408994	-3.275062561
C	-3.307458162	-1.339447021	-2.617964029
O	-2.303535700	-2.009252071	-1.983580112
H	-0.315005362	-3.196826696	-0.832508862
H	-3.885886908	0.340757132	-3.781057119
C	-4.605067730	-2.038134813	-2.494917393
C	-4.677526474	-3.292565107	-1.860386848
C	-5.789749146	-1.472517729	-3.004211664
C	-5.894747257	-3.959446430	-1.744064927
C	-7.002729416	-2.143323898	-2.887074471
C	-7.061889172	-3.390102863	-2.256982803
H	-3.775300741	-3.741771698	-1.460999608
H	-5.771459103	-0.501175642	-3.490076303
H	-5.929401398	-4.928176880	-1.251127720
H	-7.906136513	-1.688895702	-3.286847591
H	-8.010610580	-3.910829306	-2.166208744
O	2.212420702	-2.524987459	-0.675743520

H	3.050980330	-2.048020840	-0.775694311
O	2.918988943	-0.258010536	-1.874071598
H	3.079037428	0.573002934	-2.338590622
O	-1.536374927	1.513650417	-3.927903175
H	0.848944604	1.068464279	-3.127990961

==============================

$2^{\cdot+}$

==============================

C	0.592044294	0.020076586	-4.270007133
C	1.068213701	0.588319659	-3.073802948
C	0.212518021	0.666909814	-1.903742671
C	-1.077314496	0.188651890	-1.952429533
C	-1.530234694	-0.370485067	-3.163858652
C	-0.699781656	-0.458437532	-4.323655128
C	-1.260434389	-1.076542497	-5.558804989
C	-2.629783630	-1.540441036	-5.448711395
C	-3.361150980	-1.402223229	-4.307342052
O	-2.783364534	-0.817823529	-3.185571671
H	-1.743347526	0.226366922	-1.097105861
H	-3.059264183	-2.024870872	-6.315539360
C	-4.745123386	-1.807056427	-4.072577000
C	-5.259819031	-1.898674250	-2.765196085
C	-5.583134174	-2.114586115	-5.162662506
C	-6.574764252	-2.302290916	-2.557710886
C	-6.895956039	-2.514503956	-4.946273327
C	-7.395009041	-2.611922979	-3.644273043
H	-4.627621651	-1.671262264	-1.915340781
H	-5.218945980	-2.021884441	-6.179919243
H	-6.960196495	-2.379672050	-1.546300173
H	-7.534320831	-2.743523121	-5.793347836
H	-8.421830177	-2.922658443	-3.478864908
O	0.824586570	1.215757728	-0.844572723
H	0.255336285	1.272204161	-0.061940074
O	2.300549507	1.053506494	-3.019361734
H	2.493066072	1.429233789	-2.140017748
O	-0.578229249	-1.178788185	-6.577748775
H	1.231590629	-0.038156375	-5.144662380

==============================

3

==============================

C	0.588096857	0.125145242	-2.613962889
C	1.574583173	-0.616995692	-1.947385788
C	1.227159619	-1.809479833	-1.309867144
C	-0.086644784	-2.283349276	-1.320218921
C	-1.056061149	-1.540659070	-1.984007478
C	-0.742232263	-0.336028129	-2.636059046
C	-1.783281207	0.417560220	-3.326676130
C	-3.100900412	-0.174995050	-3.276803493
C	-3.337969780	-1.347811580	-2.619124889
O	-2.345815659	-2.020898581	-1.983771563
H	-0.357822388	-3.208624601	-0.825132132
H	-3.899895668	0.345261246	-3.787518978
C	-4.639975548	-2.037228584	-2.503812075
C	-4.721058846	-3.291826963	-1.870694757
C	-5.818165302	-1.463346601	-3.017751932
C	-5.942207813	-3.951674700	-1.759696364
C	-7.035604954	-2.127334118	-2.905743361
C	-7.103957653	-3.374458790	-2.276783228
H	-3.822756767	-3.746478319	-1.467994094
H	-5.792275906	-0.491754979	-3.502619743
H	-5.984667301	-4.920566082	-1.267899632
H	-7.934904575	-1.667715549	-3.308469772
H	-8.055904388	-3.889992714	-2.189962626
O	2.862016439	-0.174809635	-1.923856258
H	2.884921551	0.658716023	-2.419971704
O	0.962734878	1.273090363	-3.219012737
H	0.132143468	1.644456148	-3.630551338
O	-1.534068704	1.494310975	-3.916187763
H	2.008591890	-2.364567757	-0.801379740

==============================

$3^{\cdot+}$

==============================

C	0.611862659	0.156008393	-2.624111891
C	1.603550553	-0.662337720	-1.930295944
C	1.241073132	-1.871104360	-1.305669785
C	-0.070665039	-2.307382345	-1.339195013
C	-1.056094170	-1.531196117	-2.007763624
C	-0.749662697	-0.331691861	-2.639542580
C	-1.797413230	0.389039636	-3.287223577
C	-3.093851566	-0.146478325	-3.258115530
C	-3.342767477	-1.349253535	-2.616813183
O	-2.320780754	-2.009351015	-2.008181810
H	-0.368499428	-3.235232115	-0.864108026
H	-3.891142368	0.398506045	-3.743202209
C	-4.624386787	-2.027840137	-2.506228209
C	-4.733069420	-3.235256433	-1.779710054
C	-5.776724339	-1.490155339	-3.123831272
C	-5.958885193	-3.879578114	-1.675408363
C	-6.997121811	-2.141964436	-3.014258146
C	-7.092429638	-3.336724043	-2.290551424
H	-3.859820366	-3.662475109	-1.299062848
H	-5.728320122	-0.567767620	-3.694285393

H	-6.032969475	-4.806556702	-1.113817692
H	-7.876039505	-1.720578551	-3.493890285
H	-8.048866272	-3.843328714	-2.207305670
O	2.848662853	-0.230352357	-1.908687234
H	2.885159731	0.620161653	-2.395316839
O	0.961513519	1.232133985	-3.173462152
H	-0.603724480	1.747009158	-3.813434362
O	-1.570292711	1.526239395	-3.897058487
H	2.007881641	-2.448627234	-0.800964952

==============================
4
==============================

C	0.602669537	0.146780714	-2.616189241
C	0.709102273	1.350001335	-3.303334236
C	-0.423658073	1.926909328	-3.901963711
C	-1.673840523	1.307552338	-3.820731878
C	-1.764276147	0.104995295	-3.129240990
C	-0.651864767	-0.497456819	-2.518905878
C	-0.789461672	-1.751638651	-1.800691843
C	-2.130071878	-2.295354605	-1.775855660
C	-3.169748545	-1.666616082	-2.394448042
O	-2.999796152	-0.492229491	-3.059407473
H	-2.540693283	1.759094715	-4.286278725
H	-2.281544447	-3.223363876	-1.242054343
C	-4.571276188	-2.131990433	-2.435993433
C	-5.563089371	-1.337068319	-3.040386438
C	-4.944855690	-3.369594336	-1.878353119
C	-6.886966705	-1.766878605	-3.081815720
C	-6.268942833	-3.793638706	-1.920773387
C	-7.246653557	-2.994826317	-2.522285223
H	-5.290958405	-0.381805688	-3.475524426
H	-4.202197075	-4.011737347	-1.413492799
H	-7.639196873	-1.138418078	-3.552644253
H	-6.537784100	-4.752944469	-1.485073924
H	-8.279418945	-3.329079151	-2.555092812
O	-0.302852869	3.101151705	-4.568114281
H	0.626820624	3.373405457	-4.516903877
O	1.900818110	2.018495083	-3.427741051
H	2.575131893	1.498450875	-2.964343548
O	1.718693614	-0.369020224	-2.058382273
H	1.429903269	-1.220929742	-1.620932102
O	0.188468099	-2.307657003	-1.246036053

==============================
4$^{\bullet+}$
==============================

C	-0.916145682	0.622719884	-1.560446262
C	0.241659448	0.738670588	-0.687192440
C	1.324596524	-0.196883798	-0.761676133
C	1.267228246	-1.231492758	-1.684520245
C	0.146636426	-1.351522326	-2.538045168
C	-0.933501303	-0.470267057	-2.505082607
C	-2.011083841	-0.667855203	-3.408648729
C	-1.948728204	-1.750806808	-4.304675579
C	-0.861930847	-2.601304293	-4.290265083
O	0.158285037	-2.384084940	-3.409362793
H	2.067751408	-1.958789349	-1.766071200
H	-2.751981497	-1.889994025	-5.014681816
C	-0.650772035	-3.758244038	-5.147392273
C	0.603893697	-4.401466846	-5.186732292
C	-1.697803140	-4.243603230	-5.958631516
C	0.802377105	-5.494513035	-6.021019459
C	-1.491067529	-5.339443207	-6.785724163
C	-0.241357014	-5.965473652	-6.822103500
H	1.419816852	-4.036412716	-4.574766159
H	-2.681050301	-3.788026810	-5.925961971
H	1.772464991	-5.979670525	-6.050259113
H	-2.304932833	-5.710529327	-7.399717808
H	-0.083240323	-6.821171761	-7.471094131
O	2.326437712	0.018134575	0.093338057
H	3.027892113	-0.643421471	-0.001655332
O	0.278020561	1.726996303	0.179131106
H	-0.548930764	2.245191574	0.068338536
O	-1.843798518	1.467378497	-1.460493445
H	-2.924168825	0.837018967	-2.740408659
O	-3.046853304	0.136710972	-3.431218624

==============================
5
==============================

C	0.628104389	0.177908540	-2.629182339
C	0.731481910	1.382397413	-3.313208342
C	-0.395710051	1.969961643	-3.911417484
C	-1.648410320	1.343142748	-3.825883865
C	-1.747565508	0.131901264	-3.136994600
C	-0.626573384	-0.462636113	-2.534372568
C	-0.761925459	-1.721628428	-1.817374468
C	-2.098985434	-2.270351887	-1.788818479
C	-3.141932726	-1.642649651	-2.402586222
O	-2.973504305	-0.465551823	-3.065146208
H	-2.246250391	-3.200339317	-1.257002354
C	-4.543437481	-2.102571726	-2.445822239
C	-5.529497147	-1.299819946	-3.050447464
C	-4.921326160	-3.339980125	-1.890702486
C	-6.854821682	-1.724297166	-3.093595505

C	-6.247210026	-3.757730722	-1.935986042
C	-7.220027924	-2.952134848	-2.537053108
H	-5.251906395	-0.345147550	-3.483977318
H	-4.181718349	-3.986161947	-1.426096797
H	-7.603676796	-1.091477275	-3.563940048
H	-6.521532536	-4.716747761	-1.503010750
H	-8.254291534	-3.281520128	-2.571866989
O	-0.268715382	3.141531944	-4.569595337
H	0.663381755	3.406566143	-4.514362812
O	1.920972943	2.052346706	-3.438799620
H	2.598057747	1.532466888	-2.978785515
O	1.745530844	-0.337282360	-2.075725079
H	1.459665298	-1.191162348	-1.639388084
O	0.220699444	-2.272832632	-1.267930865
Cl	-3.043485165	2.075448513	-4.569135666

==============================

$5^{\cdot+}$

==============================

C	0.674593031	0.184547141	-2.620959997
C	0.734985352	1.438263297	-3.343966007
C	-0.406521201	2.022313118	-3.942330837
C	-1.642952323	1.371302366	-3.836285830
C	-1.726868749	0.135884121	-3.127906561
C	-0.618645191	-0.458481252	-2.531528234
C	-0.792208314	-1.689328074	-1.840660453
C	-2.075241327	-2.261088371	-1.793697357
C	-3.140696764	-1.628002882	-2.406999111
O	-2.936715841	-0.448070556	-3.055627108
H	-2.209612846	-3.196491718	-1.270525932
C	-4.521131039	-2.080222845	-2.449497700
C	-5.506659031	-1.306538582	-3.100051641
C	-4.893964767	-3.298280716	-1.840245724
C	-6.824558258	-1.743208647	-3.135287046
C	-6.213454247	-3.725347519	-1.881766558
C	-7.181798935	-2.950282812	-2.528176308
H	-5.236526966	-0.369856954	-3.572273254
H	-4.161283970	-3.916872025	-1.335152268
H	-7.576501846	-1.142730117	-3.636371374
H	-6.490045071	-4.662867546	-1.411173701
H	-8.213087082	-3.287858486	-2.558204174
O	-0.327531725	3.178042173	-4.601721764
H	0.584855378	3.511693478	-4.592206955
O	1.896927357	2.071919680	-3.451641798
H	2.578489065	1.545235634	-2.984344721
O	1.719696760	-0.289916188	-2.104830265
H	1.038486123	-1.761404157	-1.387765169
O	0.212172851	-2.297451496	-1.252923727
Cl	-3.040311813	2.059569836	-4.553271294

==============================

1a

==============================

C	-2.312644005	0.455048651	-4.714796066
C	-1.915329337	1.775379896	-4.547896385
C	-1.486560345	2.253452778	-3.300912142
C	-1.427237391	1.393432021	-2.205726385
C	-1.846564412	0.073974662	-2.382852793
C	-2.317071676	-0.447325796	-3.605090857
C	-2.899969101	-1.807131529	-3.610950708
C	-2.776634455	-2.522351027	-2.342609406
C	-2.223161936	-1.973739982	-1.236723661
O	-1.768387914	-0.689068437	-1.244507790
H	-1.921299815	2.439652205	-5.403500557
H	-1.082791328	1.701880932	-1.228115201
O	-3.477393389	-2.323941946	-4.571205139
C	-2.060397625	-2.618954659	0.079395466
C	-2.139593840	-4.015786171	0.209718332
C	-1.827265143	-1.844458103	1.227841973
H	-2.287975788	-4.634508133	-0.669022799
H	-1.760028124	-0.766632617	1.137717843
C	-2.005861759	-4.617827415	1.457022905
C	-1.692286730	-2.451906919	2.474122763
H	-1.517640710	-1.838805914	3.353486061
C	-1.783326983	-3.838862181	2.594799519
H	-2.066774368	-5.698907375	1.539963484
H	-1.676919222	-4.311103821	3.566976309
H	-3.191012383	-3.521409750	-2.314552069
O	-1.120452881	3.560359240	-3.270166397
C	-0.652396202	4.104322433	-2.043363810
H	-1.418727398	4.048218727	-1.260271192
H	0.255659014	3.594748020	-1.697596312
H	-0.422752410	5.150786400	-2.248398304
O	-2.713431120	0.134153098	-5.971427441
C	-2.124014854	-0.987122655	-6.649303913
H	-1.111745119	-1.218926549	-6.195325851
H	-2.795117617	-1.889013529	-6.559996605
H	-1.993767500	-0.659416020	-7.723827839

==============================

$1a^{\cdot+}$

==============================

C	-2.094798326	0.478520542	-4.723418236
C	-2.069221973	1.873695254	-4.479852676
C	-2.014969349	2.387866735	-3.184062243

C	-1.976806760	1.489006162	-2.100109816
C	-2.063628912	0.106882453	-2.355939388
C	-2.166919470	-0.459671587	-3.633143425
C	-2.556853056	-1.881514549	-3.708691835
C	-2.387562990	-2.622584581	-2.462898016
C	-2.169150114	-2.001855612	-1.256136060
O	-2.061624289	-0.647561014	-1.226365685
H	-2.034947872	2.544543266	-5.330243587
H	-1.897139430	1.817898989	-1.072372675
O	-3.056089163	-2.430068493	-4.691856861
C	-2.044965029	-2.634999275	0.051167645
C	-2.182804108	-4.036670208	0.183784887
C	-1.781484723	-1.857954979	1.201522827
H	-2.388358593	-4.660129547	-0.680976927
H	-1.673302531	-0.782156944	1.116713405
C	-2.058018446	-4.637405872	1.428584695
C	-1.660223961	-2.467214346	2.443068981
H	-1.458111882	-1.862026572	3.322665215
C	-1.796764135	-3.856932878	2.561411381
H	-2.165041924	-5.714766502	1.518648982
H	-1.699738383	-4.329511166	3.533776999
H	-2.528733492	-3.693589687	-2.521112919
O	-1.969002128	3.718284369	-3.072343826
C	-1.891567111	4.323544025	-1.772957444
H	-2.766710520	4.063628197	-1.168366551
H	-0.970500588	4.029758930	-1.258500218
H	-1.880080223	5.396936417	-1.954856753
O	-2.004163265	0.195879266	-6.011131287
C	-1.526178956	-1.049942493	-6.562301636
H	-0.660769641	-1.400591254	-5.994849205
H	-2.319462538	-1.797682166	-6.524058819
H	-1.242964983	-0.802629828	-7.584340572

==============================

1b

==============================

C	-2.480766058	0.613946915	-4.647583008
C	-2.376141071	1.993073702	-4.471693993
C	-1.962442636	2.511109829	-3.237765312
C	-1.645621896	1.677145362	-2.159873247
C	-1.756384134	0.308014154	-2.353281975
C	-2.166033506	-0.266937405	-3.570830584
C	-2.268043756	-1.706892848	-3.728973627
C	-1.942332983	-2.500556946	-2.533987761
C	-1.536136270	-1.836074710	-1.421876431
O	-1.436722040	-0.496250212	-1.290359855
H	-2.619838715	2.645458221	-5.304817200
H	-1.324528575	2.088006735	-1.211559415
O	-2.613417149	-2.225257397	-4.814470768
O	-2.877658606	0.129939020	-5.827625751
H	-2.882532835	-0.863373160	-5.729648590
H	-1.235133529	-2.334332228	-0.507891655
C	-2.046186924	-3.979836702	-2.516292572
C	-2.530434370	-4.641232014	-1.375315666
C	-1.642658234	-4.751212120	-3.619271755
H	-2.888283491	-4.059111118	-0.530533254
H	-1.290422797	-4.255245209	-4.515016079
C	-2.584697485	-6.033002377	-1.324448109
C	-1.703567147	-6.142144680	-3.566813231
H	-2.963927269	-6.525092602	-0.433301866
H	-1.384620190	-6.724167347	-4.426821232
C	-2.169217825	-6.788873672	-2.420637608
H	-2.216671228	-7.873559952	-2.385860205
O	-1.847139359	3.847808123	-3.027121544
H	-2.088540554	4.321131229	-3.834410667

==============================

$1b^{\cdot+}$

==============================

C	-2.531420708	0.620939612	-4.633473873
C	-2.414461374	2.013862371	-4.462475300
C	-1.951954842	2.524911165	-3.254254580
C	-1.594722629	1.659280539	-2.182971716
C	-1.723659635	0.289653480	-2.378655434
C	-2.179139376	-0.278181374	-3.571194172
C	-2.297658443	-1.710005879	-3.742256403
C	-1.937329650	-2.520953417	-2.552114487
C	-1.487287045	-1.837195516	-1.435837984
O	-1.375834703	-0.517207801	-1.331285834
H	-2.692672253	2.659776449	-5.288850784
H	-1.234791994	2.066743851	-1.245676517
O	-2.693007708	-2.205502272	-4.814980030
O	-2.967684031	0.151569366	-5.780236721
H	-2.984883547	-0.850100875	-5.710634708
H	-1.146759152	-2.325332165	-0.531170249
C	-2.029888153	-3.972923517	-2.522244215
C	-2.184925556	-4.659214020	-1.283969998
C	-1.950090647	-4.741671085	-3.716926575
H	-2.307206392	-4.101164341	-0.361954927
H	-1.841161370	-4.238966465	-4.667073727
C	-2.245284319	-6.038984299	-1.243248820
C	-1.996514916	-6.125201702	-3.661903858
H	-2.383023977	-6.548220634	-0.295297354
H	-1.916922092	-6.701648712	-4.577520370
C	-2.143929005	-6.781319141	-2.432454348

H	-2.186501503	-7.865536690	-2.397542238
O	-1.806595325	3.830054045	-3.001214504
H	-2.062258720	4.371582985	-3.762959242

==============================

4a

==============================

C	-2.495850086	0.297856897	-4.760318279
C	-2.307902575	1.678763986	-4.645618916
C	-1.983904481	2.252497911	-3.389361620
C	-1.829905033	1.440767169	-2.271367311
C	-2.024593592	0.064094625	-2.407431602
C	-2.350916147	-0.554208577	-3.625016212
C	-2.573813915	-2.020016909	-3.640499592
C	-2.451449156	-2.657354832	-2.331622839
C	-2.099647522	-1.983594775	-1.211842537
O	-1.872219920	-0.640320241	-1.239640117
H	-1.582770348	1.834579825	-1.294711351
O	-2.842745066	-2.682682037	-4.644722462
C	-1.937322259	-2.549944162	0.139808148
C	-1.732187510	-3.927209854	0.322433770
C	-1.994108558	-1.717777610	1.269941330
H	-1.652679801	-4.580968857	-0.539474845
H	-2.148181915	-0.652990520	1.139276862
C	-1.604396820	-4.459029675	1.601975560
C	-1.865135431	-2.254219055	2.548737288
H	-1.918260336	-1.598788023	3.413209677
C	-1.672412992	-3.625663519	2.720673084
H	-1.442441940	-5.525718689	1.725559473
H	-1.571118712	-4.042492867	3.718420506
H	-2.660766125	-3.718440533	-2.291923285
O	-1.840896964	3.601713181	-3.384860039
C	-1.542843461	4.244868279	-2.152631283
H	-2.330458641	4.069072247	-1.409882784
H	-0.579970241	3.910849333	-1.746329427
H	-1.490016460	5.310827255	-2.377880812
O	-2.520543098	2.484600306	-5.731614113
C	-1.348118782	3.030050516	-6.338448048
H	-0.792823851	3.667859316	-5.642665386
H	-0.688315511	2.235979795	-6.711399078
H	-1.695089579	3.631780386	-7.181032658
O	-2.901483059	-0.162590951	-5.973726273
C	-1.934099793	-0.852791488	-6.770987511
H	-1.048801184	-0.225397155	-6.937769890
H	-1.652557969	-1.800848842	-6.309566975
H	-2.422539949	-1.044840336	-7.728003502

==============================

4a$^{\cdot+}$

==============================

C	0.627109647	0.031879488	-2.765779495
C	0.734477878	1.219381094	-3.488152981
C	-0.446187049	1.741863847	-4.131551743
C	-1.669070959	1.102519393	-3.960749865
C	-1.752067804	-0.053015776	-3.201103210
C	-0.618750274	-0.654411554	-2.600306988
C	-0.875195980	-1.847124696	-1.842131019
C	-2.192234755	-2.311639309	-1.711583495
C	-3.247276545	-1.678328753	-2.335466623
O	-3.000842333	-0.582167089	-3.074815750
H	-2.553339958	1.519557118	-4.426205635
H	-2.347220421	-3.209704399	-1.131622314
C	-4.649688244	-2.075680017	-2.302589655
C	-5.607669353	-1.364797115	-3.054051876
C	-5.067895889	-3.172072887	-1.520449877
C	-6.943861485	-1.744886160	-3.022393703
C	-6.405571461	-3.544080734	-1.494136930
C	-7.346665859	-2.833325386	-2.244534969
H	-5.301426411	-0.520499825	-3.659715891
H	-4.356997490	-3.734389067	-0.926028252
H	-7.673662186	-1.192310691	-3.604953527
H	-6.716732979	-4.388072968	-0.887408733
H	-8.391368866	-3.127444029	-2.221735716
O	0.034855574	-2.583012342	-1.228237867
O	-0.492258042	2.866062641	-4.848686695
C	0.606388927	3.389833689	-5.627470016
H	1.304355145	3.938300610	-4.996730804
H	1.124196172	2.588901281	-6.159225941
H	0.132407740	4.061932564	-6.342778206
O	1.819733977	2.005288601	-3.612309217
C	3.167617083	1.597989082	-3.318653584
H	3.318640232	1.470800400	-2.246366024
H	3.427994251	0.679725170	-3.852750063
H	3.786037683	2.417463541	-3.685000896
O	1.748230934	-0.407565922	-2.071428537
C	2.359584808	-1.559911489	-2.491187096
H	0.957728267	-2.345228434	-1.477079749
H	2.202628851	-1.875200272	-3.519241095
H	3.282580614	-1.758301497	-1.959475756

==============================

4b

==============================

C	1.648309588	1.924155235	-0.514859915
C	0.751777709	2.967339993	-0.759219289
C	-0.061045669	2.968234539	-1.896378756

C	0.001043435	1.921620131	-2.807910681
C	0.896648765	0.884452820	-2.549793959
C	1.743524432	0.839929819	-1.429088473
C	2.680114746	-0.292583406	-1.236468077
C	2.627453327	-1.350647807	-2.289043665
C	1.730045319	-1.181502581	-3.296433449
O	0.902117729	-0.128797144	-3.468948603
H	-0.622320890	1.888294220	-3.695010900
O	-0.889236867	4.052478313	-2.011872768
H	-1.420359492	3.980616570	-2.814788818
O	0.710242629	3.981720686	0.167233542
H	0.052981205	4.630425930	-0.121673763
O	2.405624151	1.977738738	0.600330770
H	2.170549631	2.797074318	1.065027952
O	3.433721066	-0.336470455	-0.265754282
H	1.567227721	-1.893524885	-4.096336365
C	3.498185635	-2.568498850	-2.262601852
C	3.517677307	-3.478402615	-3.349361897
C	4.330747128	-2.863104582	-1.158927202
C	4.303789139	-4.625092030	-3.323766232
C	5.129148483	-4.007566452	-1.146401286
C	5.119703293	-4.900081635	-2.219077826
H	2.929030180	-3.300231218	-4.251565933
H	4.348384857	-2.179679155	-0.319697022
H	4.286231518	-5.300818920	-4.179534435
H	5.762012482	-4.198095322	-0.279735804
H	5.740228653	-5.791629791	-2.202835798

==============================

4b$^{\cdot+}$

==============================

C	-0.232862949	1.406538963	-4.774988651
C	0.245742068	2.191239119	-3.673032522
C	-0.144292638	1.856201172	-2.341172934
C	-0.981152475	0.785324931	-2.094317913
C	-1.436930180	0.023926299	-3.194169998
C	-1.089431286	0.304910511	-4.517041206
C	-1.584002018	-0.502346992	-5.625422478
C	-2.449637175	-1.636821032	-5.250171185
C	-2.729328156	-1.790806890	-3.917903423
O	-2.249833107	-1.011556745	-2.922144175
H	-1.289440870	0.510971129	-1.091240525
O	0.387922257	2.682588816	-1.416825294
H	0.117122918	2.458000898	-0.515302777
O	1.048008680	3.210325956	-3.925551176
H	1.299078941	3.648699760	-3.091322899
O	0.134481207	1.728150845	-5.985765457
H	-0.312179714	1.055229902	-6.603414536
O	-1.260560513	-0.225468338	-6.797255993
H	-3.387545824	-2.551956177	-3.520433903
C	-2.995789528	-2.578544378	-6.235820293
C	-3.329659462	-2.158828259	-7.543179035
C	-3.222424030	-3.927065611	-5.875404835
C	-3.892395020	-3.056201935	-8.442728996
C	-3.777497292	-4.817916393	-6.782525539
C	-4.120014668	-4.384267807	-8.069379807
H	-3.156814098	-1.133430958	-7.841187000
H	-2.921993017	-4.286599636	-4.895942688
H	-4.156686306	-2.719778538	-9.440007210
H	-3.932600260	-5.853366375	-6.497179985
H	-4.554127693	-5.081836700	-8.778875351

==============================

6

==============================

C	-2.126507282	0.601989031	-4.708448887
C	-2.115934372	1.978735924	-4.523488522
C	-2.093018055	2.523820162	-3.227982044
C	-2.080601692	1.695629120	-2.103459835
C	-2.089633703	0.320151955	-2.304336309
C	-2.112289667	-0.262949318	-3.584489584
C	-2.122800112	-1.703346729	-3.763493061
C	-2.081212282	-2.496136904	-2.527842283
C	-2.094938755	-1.820957661	-1.343991160
O	-2.088918924	-0.481319517	-1.191804051
H	-2.066879511	2.118622541	-1.107107878
O	-2.168886185	-2.211252213	-4.910055637
O	-2.149243355	0.139635846	-5.975359917
H	-2.158519506	-0.856835723	-5.890638351
H	-2.123796940	-2.314830780	-0.380135328
C	-2.035225630	-3.977102757	-2.533066750
C	-1.314281940	-4.678437710	-1.553075433
C	-2.737648487	-4.718838215	-3.502676487
H	-0.722081840	-4.134303570	-0.823851526
H	-3.278165579	-4.202028751	-4.287439823
C	-1.310209513	-6.073299408	-1.518036962
C	-2.724905252	-6.105212212	-3.468658447
H	-0.746842980	-6.613808632	-0.764802456
C	-2.017660141	-6.799302101	-2.471786261
O	-2.085692406	3.868039131	-3.069872618
H	-2.097781658	4.269024849	-3.953572273
O	-2.010292768	-8.160394669	-2.448388577
H	-2.539152145	-8.469491959	-3.200191259
O	-3.382389307	-6.907441616	-4.373646736
H	-3.796572208	-6.357590199	-5.050311089

O	-2.129260063	2.857430220	-5.576642513
H	-2.142997026	2.333509684	-6.392791271

==============================

$6^{\cdot+}$

==============================

C	-2.227174282	0.591237605	-4.709803581
C	-2.170015335	1.984547019	-4.525145054
C	-2.025994062	2.529259443	-3.232281685
C	-1.939639330	1.686829209	-2.118846416
C	-1.994070649	0.311421514	-2.324744225
C	-2.136181593	-0.275873810	-3.591377258
C	-2.199601889	-1.709892035	-3.766739130
C	-2.064286470	-2.514111996	-2.527297497
C	-1.948624849	-1.817075610	-1.342328429
O	-1.913680673	-0.493892968	-1.224406004
H	-1.834000707	2.103016615	-1.124538183
O	-2.344529867	-2.213070869	-4.901741982
O	-2.366115093	0.142951161	-5.956882477
H	-2.393357515	-0.855203450	-5.901386738
H	-1.902377844	-2.285854816	-0.367777348
C	-2.057842016	-3.970853567	-2.524805069
C	-1.481456757	-4.690269470	-1.428758740
C	-2.617203236	-4.712084770	-3.591741800
H	-0.991438210	-4.152709484	-0.625745773
H	-3.034947634	-4.191376686	-4.443635464
C	-1.481178522	-6.065736294	-1.384778976
C	-2.623768330	-6.091571331	-3.551775694
H	-1.032920122	-6.612483501	-0.562942743
C	-2.057865381	-6.793273449	-2.440963507
O	-1.972460270	3.856505632	-3.055270910
H	-2.044802904	4.304683685	-3.913393974
O	-2.063628912	-8.119333267	-2.404462814
H	-2.489494324	-8.469717979	-3.206907511
O	-3.144028664	-6.908358097	-4.495245934
H	-3.495731831	-6.415612698	-5.250398159
O	-2.246359587	2.850774765	-5.556621075
H	-2.344753504	2.362206221	-6.389840603

Appendix B. Vibrational frequencies of the optimized structures.

==============================

1

==============================

24.49	51.72	95.37	106.71	140.05	201.85
233.04	255.42	257.96	270.13	295.82	350.96
367.67	379.61	411.25	427.87	469.73	504.48
517.28	569.29	619.27	621.92	632.84	641.48
641.97	664.70	675.32	703.29	720.29	747.31
755.72	789.06	813.15	821.70	852.58	864.70
895.16	919.52	941.84	981.97	1000.08	1007.90
1016.36	1048.88	1062.23	1115.15	1127.09	1137.83
1179.91	1191.28	1213.84	1216.03	1263.31	1276.42
1302.26	1338.89	1359.78	1370.16	1404.95	1435.48
1475.71	1492.59	1510.46	1538.67	1559.21	1625.79
1638.95	1655.07	1661.77	1677.09	1719.79	3178.85
3185.81	3195.80	3198.84	3206.74	3216.66	3232.09
3244.47	3249.46	3821.29			

==============================

$1^{\cdot+}$

==============================

19.00	53.42	101.80	106.74	138.28	190.09
228.52	247.82	258.11	258.75	277.59	331.09
377.33	397.18	405.67	452.47	479.92	500.39
506.93	564.34	577.99	611.27	618.88	625.52
630.49	635.85	668.30	684.32	712.24	724.95
739.85	793.21	822.55	840.70	849.72	867.15
904.81	956.21	987.09	994.13	1011.20	1026.67
1039.30	1055.10	1093.67	1113.43	1125.52	1146.17
1180.19	1199.00	1220.83	1224.25	1259.31	1271.39
1295.81	1306.54	1354.85	1383.00	1411.03	1435.44
1464.23	1490.05	1512.55	1522.18	1540.59	1557.50
1582.47	1607.96	1625.69	1648.57	1712.25	2462.03
3205.85	3214.95	3221.66	3228.03	3230.26	3238.74
3244.28	3264.77	3790.19			

==============================

2

=============================

34.59	49.29	100.26	104.16	122.46	183.91
220.52	227.68	235.40	260.81	277.62	313.71
337.29	401.39	407.90	412.32	449.27	469.89
480.45	486.01	543.45	572.42	614.91	632.13
632.30	662.65	687.93	689.32	702.56	717.42
757.99	780.71	791.29	849.35	849.90	853.47
870.79	900.98	924.66	941.19	984.01	1008.70
1013.21	1045.85	1062.51	1083.98	1117.59	1158.59
1185.27	1191.30	1217.00	1229.81	1238.22	1261.61
1276.59	1330.60	1340.27	1360.53	1369.29	1402.33
1442.10	1490.78	1511.60	1537.84	1560.54	1621.06
1637.77	1659.80	1665.08	1684.33	1731.62	3175.15
3180.98	3193.82	3197.01	3205.31	3225.22	3235.52
3251.64	3771.52	3841.35			

==============================

$2^{\cdot+}$

==============================

27.42	47.62	89.45	98.68	125.02	176.42
207.81	223.97	253.50	264.70	322.38	329.90
367.77	384.93	405.05	409.76	450.53	456.19
476.35	519.03	559.25	600.85	605.09	628.87
629.45	652.87	678.33	691.70	694.29	697.19
754.58	764.74	787.44	839.54	840.66	849.56
874.96	889.11	933.85	948.74	988.11	996.55
1015.12	1023.08	1058.02	1070.93	1117.93	1137.14
1169.67	1190.26	1201.07	1218.96	1244.71	1259.15
1273.06	1286.51	1349.73	1374.51	1375.16	1404.19
1425.46	1490.72	1511.71	1536.30	1540.03	1545.87
1590.81	1628.26	1650.96	1653.33	1702.57	3201.14
3208.36	3215.88	3223.60	3223.84	3231.73	3232.38
3257.60	3703.30	3796.04			

==============================

3

==============================

33.97	49.08	106.65	118.93	131.65	185.61
211.82	247.57	265.35	274.32	325.32	365.85
388.85	411.16	433.68	450.30	471.81	485.78
497.49	532.37	566.76	617.74	620.74	632.35
665.53	666.26	681.82	701.12	702.69	749.21
780.91	792.51	820.28	843.85	853.91	867.60
912.72	938.10	943.83	963.98	985.68	1010.90
1013.61	1041.72	1058.43	1095.79	1119.28	1152.56
1191.96	1213.13	1220.23	1259.39	1261.09	1280.14
1326.29	1342.96	1358.53	1369.90	1396.20	1436.48
1451.62	1491.64	1516.09	1529.56	1538.92	1615.97
1639.99	1653.83	1659.09	1672.09	1709.42	3176.97
3182.81	3197.99	3199.74	3206.23	3211.55	3223.80
3227.41	3250.57	3764.93			

==============================

$3^{\cdot+}$

==============================

38.92	50.32	106.93	113.72	131.22	197.17
210.78	247.82	259.32	261.21	326.97	344.86
366.49	402.04	450.35	461.13	477.39	483.18

528.74	557.27	611.89	612.11	626.80	665.42
665.87	684.03	692.68	696.44	698.75	746.52
789.48	796.42	833.60	845.28	863.53	881.85
903.62	958.70	971.00	983.97	997.60	1011.08
1030.47	1048.09	1060.38	1100.28	1126.72	1162.59
1201.03	1206.83	1224.33	1254.89	1269.45	1286.23
1318.81	1365.62	1379.40	1387.94	1409.83	1431.26
1484.93	1507.15	1527.58	1537.80	1564.52	1579.73
1598.90	1607.20	1624.94	1649.39	1667.94	3194.96
3199.98	3209.52	3219.08	3225.40	3227.80	3238.46
3268.33	3295.42	3616.01			

==============================

4

==============================

35.13	48.46	100.59	103.16	123.79	168.71
198.51	247.65	250.01	257.10	284.23	290.72
330.92	333.63	355.82	392.91	410.59	425.59
470.09	474.06	484.05	521.05	583.64	600.44
620.41	623.87	632.58	644.81	659.39	667.36
701.20	717.03	724.01	737.56	791.50	818.00
845.32	860.66	867.67	911.77	926.77	943.17
985.03	1010.56	1013.93	1042.14	1060.29	1103.16
1117.60	1123.47	1190.80	1191.88	1217.17	1233.22
1249.83	1269.85	1309.52	1334.12	1365.76	1370.02
1380.60	1424.71	1441.15	1465.95	1491.60	1521.80
1537.58	1558.66	1616.97	1639.72	1655.75	1659.71
1676.34	1720.12	3170.44	3176.21	3182.11	3197.12
3206.02	3223.34	3241.76	3254.02	3769.13	3782.47

==============================

$4^{\cdot+}$

==============================

25.10	52.36	93.75	101.99	117.30	165.05
211.46	223.70	241.09	255.34	271.41	302.06
327.90	347.10	367.85	402.17	416.00	440.76
451.30	466.18	510.93	544.74	588.33	595.72
626.93	629.71	654.37	668.56	691.75	695.04
699.14	707.21	720.04	722.44	791.94	819.28
842.94	849.77	869.71	908.75	930.91	954.26
991.74	1013.11	1026.88	1039.60	1059.57	1105.10
1124.12	1137.64	1191.64	1199.56	1220.24	1232.24
1247.13	1277.21	1306.52	1344.95	1370.35	1380.58
1392.03	1421.02	1441.17	1484.43	1506.48	1529.37
1538.23	1567.88	1584.79	1604.92	1622.61	1631.44
1652.05	1666.53	3203.55	3211.33	3218.20	3222.54
3226.34	3234.92	3265.31	3354.96	3592.99	3798.94

==============================

5

==============================

40.55	42.72	86.42	100.24	103.03	139.45
167.96	191.25	199.22	239.18	249.86	269.65
294.63	323.43	330.92	344.47	358.56	388.12
396.34	410.45	426.69	472.45	476.22	487.88
530.89	595.32	607.86	630.50	640.72	643.14
662.35	671.63	687.09	701.38	736.05	761.99
792.50	831.20	847.13	860.21	868.81	934.88
944.10	946.26	987.60	1013.16	1013.53	1051.03
1062.88	1117.97	1120.07	1143.05	1192.36	1219.47
1240.30	1244.76	1270.46	1313.04	1335.23	1360.37
1371.63	1375.46	1420.41	1429.46	1461.94	1491.72
1513.28	1537.94	1548.94	1617.76	1637.62	1648.58
1658.96	1669.44	1715.78	3164.69	3176.21	3182.46
3195.78	3206.01	3222.18	3253.50	3762.98	3780.35

==============================

$5^{\cdot+}$

==============================

13.60	41.88	85.95	93.36	101.81	134.49
166.52	194.88	211.66	240.25	250.79	267.55
289.89	324.69	325.37	346.41	390.87	399.32
412.36	422.77	459.26	461.96	484.49	519.72
575.99	604.11	625.71	626.48	642.07	651.73
665.26	686.70	691.11	692.80	713.60	770.70
793.44	839.90	856.43	859.15	873.72	933.06
956.00	971.83	993.87	1012.63	1028.41	1053.75
1066.50	1126.03	1132.65	1167.88	1200.73	1222.83
1235.22	1250.97	1280.07	1308.16	1339.95	1352.69
1376.66	1384.72	1419.30	1450.19	1484.07	1504.45
1513.43	1530.69	1539.33	1574.76	1595.46	1606.14
1629.19	1647.73	1662.40	3204.45	3213.11	3219.86
3228.06	3237.29	3274.23	3313.13	3629.92	3761.70

==============================

1a

==============================

30.59	48.94	72.77	84.71	96.36	121.01
168.66	181.06	203.20	221.23	231.45	244.32
275.20	276.04	298.05	340.82	356.11	373.88
412.70	444.67	468.41	492.97	530.61	546.69
561.77	608.51	631.00	632.62	648.21	659.39
665.19	691.79	705.08	715.88	723.92	782.85
791.72	822.76	856.96	872.16	876.70	917.35
940.64	962.08	981.23	988.26	1005.98	1016.29
1054.31	1062.58	1077.21	1114.00	1122.86	1134.46
1177.36	1190.32	1197.88	1213.91	1230.75	1232.11

1246.32 1273.91 1278.54 1285.17 1336.62 1358.57
1368.69 1378.21 1412.74 1465.28 1474.76 1491.56
1503.78 1513.23 1517.03 1517.88 1536.97 1539.67
1550.06 1605.25 1632.92 1661.08 1662.16 1685.71
1728.28 2723.12 2787.92 2836.42 3022.60 3087.05
3155.06 3184.23 3193.74 3204.84 3213.77 3228.23
3235.36 3246.65 3248.61

==============================

1a$^{\cdot+}$

==============================

41.13 47.83 70.73 83.11 98.24 114.29
139.41 151.27 176.24 186.57 208.76 224.70
239.91 266.74 278.02 294.16 311.51 353.22
373.24 400.68 414.74 455.99 488.53 510.88
551.61 591.80 596.64 612.62 623.38 635.79
653.93 668.05 688.28 702.26 722.01 753.53
793.75 836.45 847.97 868.20 876.85 900.48
940.45 956.47 980.94 996.50 1010.08 1026.89
1033.20 1051.18 1069.93 1090.26 1122.48 1132.31
1151.20 1172.47 1187.56 1198.12 1201.21 1213.99
1223.75 1252.61 1256.62 1285.31 1301.18 1336.99
1355.26 1378.98 1388.86 1414.04 1449.54 1477.79
1482.29 1486.63 1491.06 1505.76 1508.78 1512.85
1524.30 1534.61 1578.46 1606.27 1625.10 1646.76
1681.43 3044.68 3066.87 3121.93 3157.71 3187.82
3193.63 3193.86 3197.98 3208.97 3218.82 3223.98
3241.91 3254.23 3259.74

==============================

1b

==============================

38.33 54.55 84.44 114.06 151.65 210.77
232.10 242.50 252.50 285.97 334.98 362.98
384.38 396.54 418.82 421.16 450.25 503.72
520.24 538.66 582.80 601.58 630.41 630.82
638.80 649.88 687.31 695.66 712.25 766.62
797.52 805.70 816.06 824.78 858.65 887.89
893.24 918.47 936.17 976.56 1000.82 1010.17
1016.84 1051.72 1076.32 1084.70 1113.51 1173.79
1188.96 1205.42 1214.66 1232.49 1265.41 1292.89
1333.15 1346.90 1365.37 1366.71 1405.51 1439.53
1471.37 1490.21 1507.97 1539.62 1557.42 1626.27
1636.76 1650.34 1661.63 1676.14 1713.32 3177.14
3185.11 3192.53 3194.93 3199.31 3205.99 3236.77
3238.46 3246.16 3820.79

==============================

1b$^{\cdot+}$

==============================

38.42 54.74 95.16 114.89 151.09 200.72
224.51 247.32 258.24 272.77 337.49 352.64
377.69 397.04 419.64 442.66 463.21 498.35
508.18 524.20 570.52 603.63 607.64 622.17
626.53 630.73 669.96 683.32 683.94 765.74
797.06 802.57 832.61 838.37 857.60 862.51
925.06 931.94 961.31 998.51 1001.92 1006.66
1023.00 1040.78 1063.00 1087.05 1127.19 1182.17
1193.78 1204.07 1228.45 1238.33 1267.97 1307.58
1328.14 1340.96 1371.56 1387.85 1403.62 1433.32
1466.14 1481.59 1504.94 1518.03 1542.54 1565.00
1569.10 1590.29 1632.59 1657.69 1695.68 3080.92
3204.56 3210.72 3218.55 3220.56 3227.29 3245.16
3254.04 3272.12 3796.69

==============================

4a

==============================

30.21 31.16 53.14 69.19 78.10 83.25
98.80 126.96 131.72 140.08 151.66 179.71
190.38 212.08 230.51 233.00 244.07 277.84
289.36 323.14 339.83 345.82 362.52 412.74
442.01 448.84 480.85 497.84 518.83 563.07
606.29 632.44 634.10 647.56 655.54 669.63
705.27 706.67 734.97 753.46 775.73 788.75
825.31 856.59 865.05 875.40 925.10 940.21
974.22 981.23 1005.39 1013.98 1017.87 1055.15
1062.69 1076.31 1114.14 1129.39 1163.34 1171.47
1173.50 1177.88 1189.78 1201.70 1209.48 1213.66
1222.05 1232.58 1249.51 1273.65 1296.47 1333.25
1337.74 1368.30 1381.80 1396.46 1448.50 1472.47
1485.10 1491.43 1492.30 1498.15 1504.01 1506.44
1515.71 1516.91 1524.72 1527.69 1539.33 1604.93
1632.69 1647.09 1662.60 1680.64 1731.37 3023.09
3023.93 3026.73 3089.49 3096.41 3125.21 3144.52
3155.82 3162.35 3184.09 3193.77 3205.11 3214.70
3226.85 3243.90 3245.56

==============================

4a$^{\cdot+}$

==============================

31.81 32.53 55.25 69.28 85.54 106.25
118.57 140.39 158.73 160.53 177.62 192.09
206.52 217.58 228.79 235.00 245.23 274.68
292.88 313.04 340.88 364.43 384.64 408.28
421.53 446.14 457.90 484.08 500.34 555.10
608.50 610.87 629.76 642.23 655.32 660.77

691.79	700.31	708.35	728.46	758.49	788.77
841.57	853.01	876.35	880.24	902.55	944.75
948.10	979.63	987.25	1013.57	1015.33	1020.36
1040.70	1060.76	1120.02	1133.68	1151.54	1157.86
1171.31	1172.48	1196.73	1198.41	1209.13	1214.44
1219.66	1235.71	1260.37	1282.83	1312.07	1339.37
1363.05	1373.41	1374.87	1420.58	1436.87	1448.95
1479.00	1485.55	1491.66	1495.01	1497.85	1499.38
1502.25	1505.53	1523.35	1533.56	1536.82	1548.45
1628.93	1649.21	1655.62	1667.76	1728.22	3050.35
3080.72	3091.92	3130.17	3176.52	3191.52	3192.52
3198.22	3205.52	3205.80	3213.50	3214.11	3221.55
3228.65	3253.41	3258.50			

===============================
4b
===============================

29.25	32.22	72.77	117.53	127.64	145.39
185.40	217.55	238.57	264.39	284.40	288.97
305.40	332.24	338.07	353.21	360.11	395.27
413.56	446.75	462.33	500.55	520.88	543.78
586.52	597.81	602.62	629.11	635.91	646.54
665.37	686.56	710.12	763.45	772.37	783.07
810.15	814.43	868.19	908.41	933.73	944.88
987.79	1009.02	1022.83	1047.54	1068.55	1078.89
1116.86	1128.01	1173.92	1191.91	1206.77	1231.39
1250.36	1263.52	1301.16	1320.26	1337.89	1352.99
1379.35	1400.85	1427.92	1438.96	1488.40	1526.25
1537.95	1543.69	1614.88	1639.46	1656.37	1660.53
1688.33	1727.23	3127.62	3152.66	3156.33	3195.79
3211.90	3236.66	3246.68	3751.00	3810.19	3841.19

===============================
4b$^{\bullet+}$
===============================

38.52	56.09	86.31	112.12	127.71	170.67
201.65	231.70	245.32	269.43	311.47	316.32
333.78	336.86	374.28	397.41	406.45	414.18
442.38	460.89	517.54	535.80	560.93	590.46
605.46	611.39	621.66	641.90	652.56	689.56
692.68	702.09	765.39	780.64	800.28	826.85
831.94	850.04	909.16	949.59	951.95	990.99
991.39	1011.53	1019.74	1049.58	1074.41	1114.34
1122.64	1136.80	1168.59	1195.54	1217.25	1229.20
1272.94	1308.62	1332.24	1342.60	1364.87	1377.49
1392.69	1424.55	1427.48	1469.10	1487.20	1528.33
1530.67	1558.63	1571.00	1595.99	1597.88	1635.32
1658.34	1692.64	2870.83	3193.97	3203.98	3213.98
3222.45	3224.96	3254.00	3263.83	3707.84	3818.78

===============================
6
===============================

32.83	40.59	64.65	108.26	116.63	156.56
185.03	206.68	216.88	226.28	233.52	276.87
290.14	309.28	310.96	331.95	337.33	365.45
374.43	412.68	414.18	441.84	453.74	465.32
467.19	480.74	488.55	530.69	588.33	599.54
602.37	612.49	632.25	636.20	651.75	661.39
691.03	708.59	767.10	780.96	803.91	821.63
830.07	833.27	855.92	860.74	899.52	918.12
940.90	994.12	1081.30	1084.04	1135.60	1150.68
1182.19	1190.51	1211.69	1234.43	1256.82	1277.81
1306.69	1323.98	1342.32	1353.37	1380.98	1382.50
1411.77	1425.25	1447.94	1466.78	1481.56	1518.45
1556.52	1574.81	1625.51	1652.31	1654.88	1669.4
1672.73	1714.61	3170.99	3193.27	3216.29	3219.62
3239.05	3243.96	3763.48	3773.02	3776.53	3840.53

===============================
6$^{\bullet+}$
===============================

31.03	36.62	73.76	109.64	111.18	149.83
184.16	203.73	219.92	230.35	265.85	292.46
303.77	318.50	328.40	353.28	354.36	368.56
401.53	404.80	411.71	445.75	454.95	464.47
475.14	481.96	522.87	576.18	578.55	597.42
601.11	609.88	625.18	630.80	647.47	665.45
687.93	714.58	765.10	779.20	805.05	826.11
831.64	846.43	861.34	888.31	904.83	919.91
970.35	985.52	1070.81	1095.14	1147.64	1166.57
1175.34	1196.42	1203.15	1228.57	1273.39	1279.34
1315.18	1345.99	1366.80	1372.22	1380.33	1401.95
1410.66	1418.06	1429.72	1464.08	1507.32	1511.15
1552.53	1562.17	1566.79	1578.10	1609.20	1640.40
1664.14	1683.44	3172.23	3222.11	3236.26	3243.37
3246.53	3255.38	3725.57	3767.09	3767.62	3813.76
